# Nutritional stress targets LeishIF4E-3 to storage granules that contain RNA and ribosome components in *Leishmania*

**DOI:** 10.1371/journal.pntd.0007237

**Published:** 2019-03-14

**Authors:** Rohit Shrivastava, Matan Drory-Retwitzer, Michal Shapira

**Affiliations:** 1 Department of Life Sciences, Ben-Gurion University of the Negev, Beer-Sheva, Israel; 2 Department of Computer Sciences, Ben-Gurion University of the Negev, Beer-Sheva, Israel; McGill University, CANADA

## Abstract

*Leishmania* parasites lack pathways for *de novo* purine biosynthesis. The depletion of purines induces differentiation into virulent metacyclic forms. *In vitro*, the parasites can survive prolonged periods of purine withdrawal changing their morphology to long and slender cells with an extended flagellum, and decreasing their translation rates. Reduced translation leads to the appearance of discrete granules that contain LeishIF4E-3, one of the six eIF4E paralogs encoded by the *Leishmania* genome. We hypothesize that each is responsible for a different function during the life cycle. LeishIF4E-3 is a weak cap-binding protein paralog, but its involvement in translation under normal conditions cannot be excluded. However, in response to nutritional stress, LeishIF4E-3 concentrates in specific cytoplasmic granules. LeishIF4E-3 granulation can be induced by the independent elimination of purines, amino acids and glucose. As these granules contain mature mRNAs, we propose that these bodies store inactive transcripts until recovery from stress occurs. In attempt to examine the content of the nutritional stress-induced granules, they were concentrated over sucrose gradients and further pulled-down by targeting *in vivo* tagged LeishIF4E-3. Proteomic analysis highlighted granule enrichment with multiple ribosomal proteins, suggesting that ribosome particles are abundant in these foci, as expected in case of translation inhibition. RNA-binding proteins, RNA helicases and metabolic enzymes were also enriched in the granules, whereas no degradation enzymes or P-body markers were detected. The starvation-induced LeishIF4E-3-containing granules, therefore, appear to store stalled ribosomes and ribosomal subunits, along with their associated mRNAs. Following nutritional stress, LeishIF4E-3 becomes phosphorylated at position S75, located in its less-conserved N-terminal extension. The ability of the S75A mutant to form granules was reduced, indicating that cellular signaling regulates LeishIF4E-3 function.

## Author summary

Cells respond to cellular stress by decreasing protein translation, to prevent the formation of partially folded or misfolded new polypeptides whose accumulation can be detrimental to living cells. Under such conditions, the cells benefit from storing inactive mRNAs and stalled ribosomal particles, to maintain their availability once conditions improve; dedicated granules offer a solution for such storage. *Leishmania* parasites are exposed to a variety of stress conditions as a natural part of their life cycle, including the nutritional stress that the parasites experience within the gut of the sandfly. Thus, *Leishmania* and related trypanosomatids serve as a good model system to investigate RNA fate during different stress conditions. Various granules appear in *Leishmania* and related organisms in response to different stress conditions. Here, we investigated how nutritional stress, in particular elimination of purines, induced the formation of granules that harbor a specific cap-binding protein, LeishIF4E-3. The starvation-induced LeishIF4E-3 containing granules consist of a variety of ribosomal proteins, along with RNA-binding proteins and mature mRNAs. We thus propose that *Leishmania* modulates the assembly of LeishIF4E-3-containing granules for transient storage of stalled ribosomal particles and inactive mRNAs. Following renewal of nutrient availability, as occurs during the parasite’s life cycle, the granules disappear. Although their fate is yet unclear, they could be recycled in the cell. Unlike other granules described in trypanosomes, the LeishIF4E-3-containing granules did not contain RNA degradation enzymes, suggesting that their function is mainly for storage until conditions improve.

## Introduction

*Leishmania* are heteroxenous pathogenic parasites that live in the alimentary tract of sandflies as extracellular flagellated promastigotes. Upon transmission to the mammalian host, the parasites enter macrophages and become obligatory intracellular and non-motile amastigotes, residing within phagolysosomes [1]. While cycling between vector and host, *Leishmania* parasites must overcome extreme physiological changes in their host milieu, experiencing temperature and pH switches, which serve as environmental signals to undergo differentiation from one life form to another [2–4]. In addition to the environmental changes, the parasites also experience changes in their nutritional supplies, based on the availability of nutrients from the vector or host [5, 6]. The changes described are known to drive a developmental program of gene expression. Since processes that control gene expression in trypanosomatids are mostly post-transcriptional, translation regulation plays a key role in establishment of the stage-specific expression profiles [7, 8].

Trypanosomatids lack enzymes for the *de novo* biosynthesis of purines and the parasites are entirely dependent on the host for purine supply [9–11]. Furthermore, the lack of purines was shown to induce metacyclogenesis [12]. The parasites are ingested with a blood drop that continues to provide essential nutrients such as purines, until their consumption. Although the sandfly's gut is co-inhabited by a rich repertoire of bacteria, these do not provide the parasites with nutrients, and were even shown to compete for food sources, generating nutrient stress and differentiation into metacyclics [13, 14]. The pathway for metacyclogenesis in *Leishmania* can be triggered *in vitro* by depletion of purines, using adenosine receptor antagonists that inhibit purine uptake. Accordingly, replenishing the adenosine supply reduces the induction of metacyclogenesis [12]. Metacyclic promastigotes are growth-arrested, and equipped with a long flagellum.

Nutrient stress requires the swift regulation of gene expression that includes control of translation [15]. As a consequence of impaired translation cytosolic mRNAs and stalled ribosomes are often stored in dedicated cytoplasmic RNA-protein granules [16]. These cytoplasmic granules are membrane-less aggregates of RNA and proteins [17]. Trypanosomatids harbor a wide repertoire of cytoplasmic granules that include P-bodies, stress granules, heat shock granules, nuclear periphery granules and tRNA halves [18–21]. In higher eukaryotes, P-bodies contribute to normal cellular physiology and are also claimed to be associated with mRNA decay [15, 22]. Accumulation of mRNAs in granules occurs in response to cellular stresses when translation is inhibited [23, 24], and the mRNAs can be dynamically altered between the different granules [25]. Starvation-induced stress granules assemble in *Trypanosoma cruzi* [20] during the nutritional stress that also occurs within the insect vector. Such granules can store mRNAs and translation complexes [26], yet can also lead to mRNA degradation [27].

Cap-dependent translation is the default mechanism for protein synthesis under normal conditions. It is based on assembly of a cap-binding complex onto the 5' cap structure through the cap-binding protein eIF4E, along with its binding partner and scaffold protein eIF4G. The latter serves as a hub for the DEAD-box RNA helicase eIF4A and for other subunits of the initiation complex. The initiation complex scans the 5' UTR until the first AUG is reached, where the large ribosomal subunit is recruited [28]. A variety of cellular stresses can lead to a global arrest of protein synthesis. In yeast and mammals, this can be achieved by inhibition of cap-dependent translation, although specific proteins may continue to be synthesized via cap-independent pathways [28]. While it is yet unclear whether stress-induced differential regulation of gene expression in *Leishmania* follows a similar pattern, we previously showed that the LeishIF4E-4 cap-binding complex in *Leishmania* is inactivated in axenic amastigotes [29]. The trypanosomatid genomes encode for a rather large number of cap-binding complexes that include six paralogs of eIF4E (1–6) and five identified paralogs of eIF4G [29–32]. The different eIF4E paralogs show different patterns of expression and cap-binding activity throughout the parasite’s life cycle, supporting the possibility that each paralog performs a different role under the changing environmental conditions. It is generally accepted that LeishIF4E-4 and LeishIF4G-3 form a canonical eIF4F complex as part of translation initiation, with LeishIF4E-1 seemingly also playing an additional, as yet unresolved role in axenic amastigotes at elevated temperatures [29]. In trypanosomes, the ortholog of LeishIF4E-3 is essential for viability [33], and could be involved in translation initiation. However, its role in *Leishmania* remains unclear due to its relatively weak cap-binding activity [30, 34]. Whereas LeishIF4E-3 interacts with LeishIF4G-4 under normal conditions, this interaction is impaired during nutritional stress, causing LeishIF4E-3 to concentrate within specific cytoplasmic granules [34].

Here, we studied the fate of LeishIF4E-3 during nutritional stress. We show that under such conditions whereby translation slows down, LeishIF4E-3 is phosphorylated and migrates into dedicated granules. We identified the site of its phosphorylation (serine 75) and show that the S75A mutant fails to enter the starvation-induced granules. We further examined the effect of depleting specific nutrients on LeishIF4E-3 phosphorylation and its ability to enter granules in response to the depletion of purines, amino acids and glucose. We found that only purine starvation induced the morphological changes generating cells that resemble the nectomonad stage, prior to metacyclogenesis. To further examine the interactome of the LeishIF4E-3 containing granules induced following nutrient depletion, we collected the heavy fractions enriched for LeishIF4E-3 from sucrose gradients, affinity purified them through tagged LeishIF4E-3 and assessed their content. The LeishIF4E-3 containing granules contain mRNA and a multitude of ribosomal particles, along with other proteins that accompany mRNAs, supporting their suggested function as storage bodies.

## Materials and methods

### Cell culture, growth and starvation conditions

Cutaneous infection-causing *Leishmania amazonensis* (*L*. *amazonensis*, strain MHOM/BR/LTB0016) promastigotes were used in this study. Wild type *L*. *amazonensis* promastigotes were routinely cultured in Medium 199 (pH 7) supplemented with 10% fetal calf serum (FCS, European Grade; Biological Industries), 4 mM L-glutamine, 0.1 mM adenine, 5 μg/ml hemin, 40 mM Hepes, pH 7.5, 100 U/ml penicillin and 100 μg/ml streptomycin and grown at 25°C. All nutritional starvation treatments were performed in Dulbecco’s modified eagle medium (DMEM; Biological Industries). Purine starvation was performed in DMEM supplemented as above, except for adenine and FCS that were not included in the medium. Amino acid starvation was performed in custom-made DMEM lacking essential amino acids (i.e., Arg, His, Trp, Phe, Ser, Tyr, Thr, Val, Leu, Lys, Pro, Met, Cys and Ile) [35] [36] and supplemented as described above, except for the absence of FCS. Glucose starvation was performed in DMEM lacking glucose, supplemented as described above, except for the absence of FCS. Control cells used in the starvation assays were also grown in DMEM supplemented as described above. All experiments were performed with logarithmically growing *L*. *amazonensis* cells at a cell density of 4–6 x 10^6^ cells/ml. For FCS dialysis, 2000 MW cut-off dialysis tubing was used and the FCS was dialyzed against PBS overnight at 4ºC with stirring. All experiments with dialyzed FCS were performed with DMEM supplemented with 10% FCS.

### Plasmids and cloning for transgenic parasites

LeishIF4E-3 was tagged with streptavidin-binding protein (SBP). Accordingly, the gene for LeishIF4E-3 was PCR amplified using LeishIF4E3 primers: Forward- ACTGGATCCATGAACCCGTCTGCCGCTGC, reverse- GCTCTAGAACAGAACGTGTGATCG and was cloned into the BamHI and XbaI sites of the pX H-SBP-H plasmid cassette [H represents the *HSP83* intergenic regions required for processing of the transgene transcript] [29]. The pX H-SBP-H plasmid cassette is designed for cloning of the DNA coding region fused to a C-terminal SBP tag. *L*. *amazonensis* cells were transfected as previously described [37]. A stable cell line expressing the fusion protein was generated using 200 μg/ml G-418 selection.

### Western blot analysis

Wild type *L*. *amazonensis* promastigotes were cultured to a late log phase of growth (4–6 x 10^6^ cells/ml) and starved as described above for different times. The cells were harvested, washed twice in ice cold phosphate buffer saline (PBS) pH 7.4, and once in PRS buffer (35 mM Hepes, pH 7.5, 100 mM KCl, 10 mM MgCl_2_, 1 mM DTT) before being resuspended in PRS+ buffer. PRS+ buffer consisted of 2x cocktail of protease inhibitors (Sigma), 4 mM iodoacetamide (Sigma), 25 mM sodium fluoride, 55 mM β-glycerophosphate and 5 mM sodium orthovanadate. Cells were lysed in 5x sample buffer and heated at 95°C for 5 minutes. Cell lysates were stored at -80°C. Cell extracts (equal protein loads) were resolved on 12% SDS-polyacrylamide (SDS-PAGE) gels and probed using specific antibodies against LeishIF4E-3 or LeishIF4G-4. Protein loads were verified using specific antibodies against LeishIF4A1. All the primary antibodies were used at 1:5000 dilutions.

### Protein dephosphorylation assays

Wild type log phase parasites were exposed to specific starvation conditions along with control non-starved cells for designated periods. The cells were washed twice in ice cold PBS and resuspended in λ-phosphatase buffer (Sigma) supplemented with 2 mM MnCl_2_ (Sigma) and 2x cocktail of protease inhibitors (Sigma). The cells were lysed using 1% Triton X-100 and 10 units of λ-phosphatase were added to the lysates, with or without phosphatase inhibitors (sodium fluoride, β-glycerophosphate and sodium orthovanadate). After incubation at 30°C for 30 min, the reaction was stopped by adding 5x Laemmli sample buffer and heating for 5 mins at 95°C. Protein mixtures were resolved on 12% low bis-acrylamide (30:0.2) containing SDS-PAGE and immunoblotted using antibodies against LeishIF4E-3.

### Identification of phospho-peptides

Cell lysates from wild type *L*. *amazonensis* promastigotes starved for different periods were prepared as described above and resolved on 12% reduced bis-acrylamide SDS-PAGE gels. Protein mixtures were allowed to resolve for an extended duration of 4 h, followed by staining with Coomassie blue. Protein bands between 35 and 48 kDa (the molecular mass of LeishIF4E-3 is ~37 kDa) were sent for phospho-peptide mapping to the Smoler Proteomics Center at the Technion, Israel.

#### Sample preparation and phospho-enrichment

The proteins in the gel were reduced with 3 mM DTT (60ºC for 30 min), modified with 10 mM iodoacetamide in 100 mM ammonium bicarbonate and digested in 10% acetonitrile and 10 mM ammonium bicarbonate with trypsin (Promega) overnight at 37°C. The tryptic peptides were eluted with different gradients of acetonitrile with formic acid in water, dried and re-suspended in 0.1% formic acid. The resulting peptides were resolved by reverse-phase chromatography. 20% of the peptides were analyzed by LC-MS/MS and the remaining 80% of the peptides were enriched for phospho-peptides on titanium dioxide (TiO_2_) beads.

#### Mass spectrometry analysis

Mass spectrometry was performed with a Q-Exactive plus mass spectrometer (Thermo) in a positive mode using repetitively full MS scan followed by High energy Collision Dissociation (HCD) of the 10 most dominant ion selected from the first MS scan. The mass spectrometry data was analyzed with Proteome Discoverer software, version 1.4, using Sequest algorithm, against the *L*. *amazonensis* LeishIF4E-3 derived sequence [38]. Results were filtered with 1% false discovery rate. The position of the phosphorylation in the peptide was validated with the phosphoRS tool. The relative quantities of the phospho-peptides were determined using the peak area calculation based on its extracted ion currents (XICs).

### Confocal microscopy

For confocal microscopy, mid-log phase transgenic LeishIF4G4-GFP expressing *L*. *amazonensis* promastigotes (~1 x 10^7^) were washed with PBS and fixed in 2% paraformaldehyde for 30 min. The cells were washed once with PBS, allowed to adhere to poly-L-lysine-coated slides and permeabilized with 0.1% Triton X-100 for 10 min, followed by blocking with 2% bovine serum albumin (BSA) for 1 h. LeishIF4E-3 was detected using anti-LeishIF4E-3 serum (1:50), followed by incubation with secondary DyLight 550 goat anti-rabbit IgG (1:500, KPL). DNA was stained with DAPI (Sigma). Finally, the cells were washed thrice with PBS and an anti-bleach mounting solution (DABCO) was added prior to the placement of cover slips. The slides were examined in an inverted Zeiss LSM 800 spinning disc confocal microscope with Airyscan at a magnification of x63. A single representative section is presented in all figures.

### Fluorescence *in situ* hybridization

Mid-log phase parasites were grown in complete medium or exposed to specific starvation conditions, for 24 h. The cells (1x10^7^) were then counted, collected by centrifugation, washed once with PBS and fixed using 2% paraformaldehyde. After being pelleted by centrifugation, the cells were resuspended in PBS and allowed to stick to poly-L-lysine-coated slides, permeabilized with 0.1% Triton X-100 and washed in 25 mM ammonium chloride for 10 min. After blocking with 2% BSA for at least 1 hour, the slides were pre-hybridized in hybridization solution (2% BSA, 5x Denhart's solution, 4x SSC, 5% dextran sulphate, 35% deionized formamide, 0.5 mg/ml yeast tRNA and 20 units RNasin (RNase inhibitor). The cells were then hybridized using PCR-amplified digoxigenin (DIG)-labeled DNA probes against *HSP83* and the *HSP83* intergenic region. The probes were synthesized using standard PCR conditions and the following forward and reverse primers for *HSP83*: 5'-AGGTGACGAAGGAGTACGAGG-3', 5'-CCGAACTGCTCGTAGAACTGC-3' and for the *HSP83* intergenic region: 5’-GAGGCACAAAGAGAGGGAAAAC-3’, 5’- GAGGGCGACGGAGATGGAAG-3’ (thus spanning positions 891–1120 of the intergenic region). The DIG-labelled probes (7 μl, following boiling) was mixed with the hybridization solution and applied to cells. Hybridization took place overnight under humid conditions. The slides were then washed once with 4x SSC and 35% formamide in PBS, followed by washes with 4x and 2x SSC and with PBS. The slides were then treated with a standard confocal procedure for staining by antibodies, beginning with blocking, followed by incubation with anti-LeishIF4E-3 antibodies (1:50) and FITC-labeled anti-DIG antibodies (1:20). Slides were then incubated with secondary DyLight 550 goat anti-rabbit IgG (1:500) and DAPI (1 μg/ml), washed in PBS and cover slips were mounted using DABCO. The slides were examined in an inverted Zeiss LSM 800 spinning disc confocal microscope with Airyscan at the magnification of x63.

### Purification of LeishIF4E-3-containing stress granules

Approximately 1 x 10^9^ SBP-tagged LeishIF4E-3-expressing transgenic parasites, grown under normal (control) or under starvation conditions for 12 hours, were incubated with 100 μg/ml cycloheximide (Sigma) for 5 minutes. The cells were washed twice with ice cold PBS containing 100 μg/ml cycloheximide, lysed in lysis buffer (15 mM Tris-HCl, pH 8, 150 mM KCl, 5 mM MgCl_2_, 0.5 mM DTT, 100 μg/ml cycloheximide, 0.5 mg/ml heparin, 4 mM iodoacetamide, 2x protease inhibitors, 25 mM sodium fluoride, 55 mM β-glycerophosphate and 100 units RNase inhibitor) containing 1% Triton X-100 for 5 min. Lysates were clarified by centrifugation at 20,000g at 4°C for 20 min. Cell lysates equivalent to 50 OD_260_ units were layered over a 12 ml 10–40% sucrose gradient prepared in gradient buffer (40 mM Tris-HCl, pH 8, 280 mM KCl, 10 mM MgCl_2_, 1 mM DTT, 200 μg/ml cycloheximide, 1 mg/ml heparin, 8 mM iodoacetamide, 4x protease inhibitors, 50 mM sodium fluoride, 110 mM β-glycerophosphate, 10 units RNasin and varying concentrations of sucrose solution). The gradients were centrifuged for 160 min at 154,000g in a SW 40Ti rotor (Beckman Coulter). Fractions (300 μl) were collected from the top and optical density was measured at 260 nm. Alternate fractions were diluted 1:1 with lysis buffer and 7 μl StrataClean Resin (Agilent Technologies) was added and the samples were mixed over overnight at 4°C in a rotator. The fractions were centrifuged at 1000g for 3 min and the supernatant was discarded. The washed resin was mixed with 5x Laemmli sample buffer and heated at 95°C for 5 minutes. Proteins bound to the beads were resolved by 12% SDS-PAGE and western analysis was performed using antibodies against LeishIF4E-3 or LeishIF4G-4.

The second set of collected fractions was diluted with gradient buffer to reduce sucrose concentration to less than 10% and aliquots were used in a pull-down assay. The aliquots were incubated with streptavidin-Sepharose beads (GE Healthcare) for 2 h on a rotator at 4°C. Following binding, the flow-through fraction was collected and the beads were washed with 0.1% NP-40 containing gradient buffer. Bound LeishIF4E-3-SBP was eluted in gradient buffer using 5 mM biotin. Proteins from the eluted fractions were tricholoroacetic acid (TCA) precipitated and washed with acetone before the addition of 5x Laemmli sample buffer. Aliquots of the eluted protein complexes were resolved by 12% SDS-PAGE and probed using antibodies against LeishIF4E-3 or LeishIF4G-4. The remaining material was resolved by 12% SDS-PAGE and those heavy fractions containing LeishIF4E-3 were subjected to mass spectrometry analysis.

### Mass spectrometry analysis

*Protein preparation from isolated granules*: To characterize the proteins enriched in the starvation-induced LeishIF4E-3 containing granules in *L*. *amazonensis*, cells expressing SBP-tagged LeishIF4E-3 were starved, extracted and pulled-down over streptavidin-Sepharose beads followed by elution with biotin, as described above. A cell line expressing SBP-tagged luciferase was similarly treated and analyzed in parallel. The pulled-down proteins were precipitated using 10% TCA and the pellets were washed with acetone. Protein pellets were dissolved in 5x sample buffer, heated and resolved by 12% SDS-PAGE. Mass spectrometric analysis was performed by the Smoler Proteomics Center at the Technion, Israel.

*Mass Spectrometry*: Proteins in the gel were reduced using 3 mM DTT (60°C for 30 min), followed by modification with 10 mM iodoacetamide in 100 mM ammonium bicarbonate for 30 min at room temperature. This was followed by overnight digestion in 10 mM ammonium bicarbonate in trypsin (Promega) at 37°C. Trypsin-digested peptides were desalted, dried, re-suspended in 0.1% formic acid and resolved by reverse phase chromatography over a 30 min linear gradient with 5% to 35% acetonitrile and 0.1% formic acid in water, a 15 min gradient with 35% to 95% acetonitrile and 0.1% formic acid in water and a 15 min gradient at 95% acetonitrile and 0.1% formic acid in water at a flow rate of 0.15 μl/min. Mass spectrometry was performed using a Q-Exactive plus mass spectrometer (Thermo) in positive mode set to conduct a repetitively full MS scan followed by high energy collision dissociation of the 10 dominant ions selected from the first MS scan. A mass tolerance of 10 ppm for precursor masses and 20 ppm for fragment ions was set.

#### Statistical analysis for enriched proteins

Raw mass spectrometric data were analyzed using the MaxQuant software, version 1.5.2.8 [39]. The data were searched against annotated *L*. *major Friedlin* proteins listed in the TriTrypDB database [40]. Protein identification was set at less than a 1% false discovery rate. The MaxQuant settings selected were a minimum of 1 razor/unique peptide for identification, a minimum peptide length of six amino acids and a maximum of two mis-cleavages. For protein quantification, summed peptide intensities were used. Missing intensities from the analyses were substituted with values close to baseline only if the values were present in the corresponding analyzed sample. The log_2_ of iBAQ intensities [41] were compared between the three SBP-LeishIF4E-3 biological repeats and the three SBP-luciferase repeats on the Perseus software platform [42], using a t test. Adjusted p-values were corrected using permutation-based false discovery rate (FDR) = 0.05 and the number of randomizations = 250. These are marked as Padj [42]. The enrichment threshold was set to a log_2_ fold change > 1.0 and p< 0.05. The annotated proteins were first categorized manually, and the summed relative intensities for each category were shown.

*Gene Ontology (GO)* term enrichment. Enriched proteins were classified by the GO Annotation tool in TriTrypDB, based on cellular components. The threshold for the calculated enrichment of proteins based on their GO terms was set for four fold, with a p<0.01. This threshold eliminated most of the general groups that represented parental GO terms. GO terms for which only a single protein was annotated were filtered out as well. In some cases, GO terms that were included in other functional terms are not shown, leaving only the representative GO term (i.e. accession numbers of ribosomal subunits are all included in the ribosome GO term).

### Translation assay

Translation was measured using the SUnSET (Surface SEnsing of Translation) method [43]. Wild type mid-log phase parasites were subjected to specific starvation treatments, while control cells were maintained under normal growth conditions. The cells were treated with 1 μg/ml puromycin (Sigma) for 20 min, washed twice with ice cold PBS, once with PRS buffer and finally resuspended in PRS^+^ buffer and lysed upon addition of 5x Laemmli sample buffer. Cycloheximide treated control parasites served as negative controls. Samples were resolved by 12% SDS-PAGE. Western analysis was performed using anti-puromycin antibodies.

### Growth and viability analysis of promastigotes

Wild type mid-log phase *L*. *amazonensis* promastigotes were exposed to different starvation conditions along with the control organisms for up to 5 days. Each day, 50 μl of cells were fixed using 4% paraformaldehyde and counted. Cell viability was measured using the trypan blue exclusion assay.

### Microscopic analysis and video recording

Wild type mid-log phase *L*. *amazonensis* promastigotes were exposed to different starvation conditions for 24 h. Cells were then washed, fixed and coverslips were mounted. Phase contrast images of parasites were captured at 100x magnification with a Zeiss Axiovert 200M fluorescence microscope equipped with a AxioCam HRm CCD camera.

Motility patterns of the parasites were examined by video-microscopy. Mid-log phase parasites were grown in either complete medium or specific nutrient-depleted medium for 24 h. Cells were then observed using the 40x objective of an Olympus IX73 inverted microscope. Videos were captured with a QImaging Retiga 6000 monochrome CCD camera.

## Results

### Nutritional stress inhibits translation

**Fig 1:**
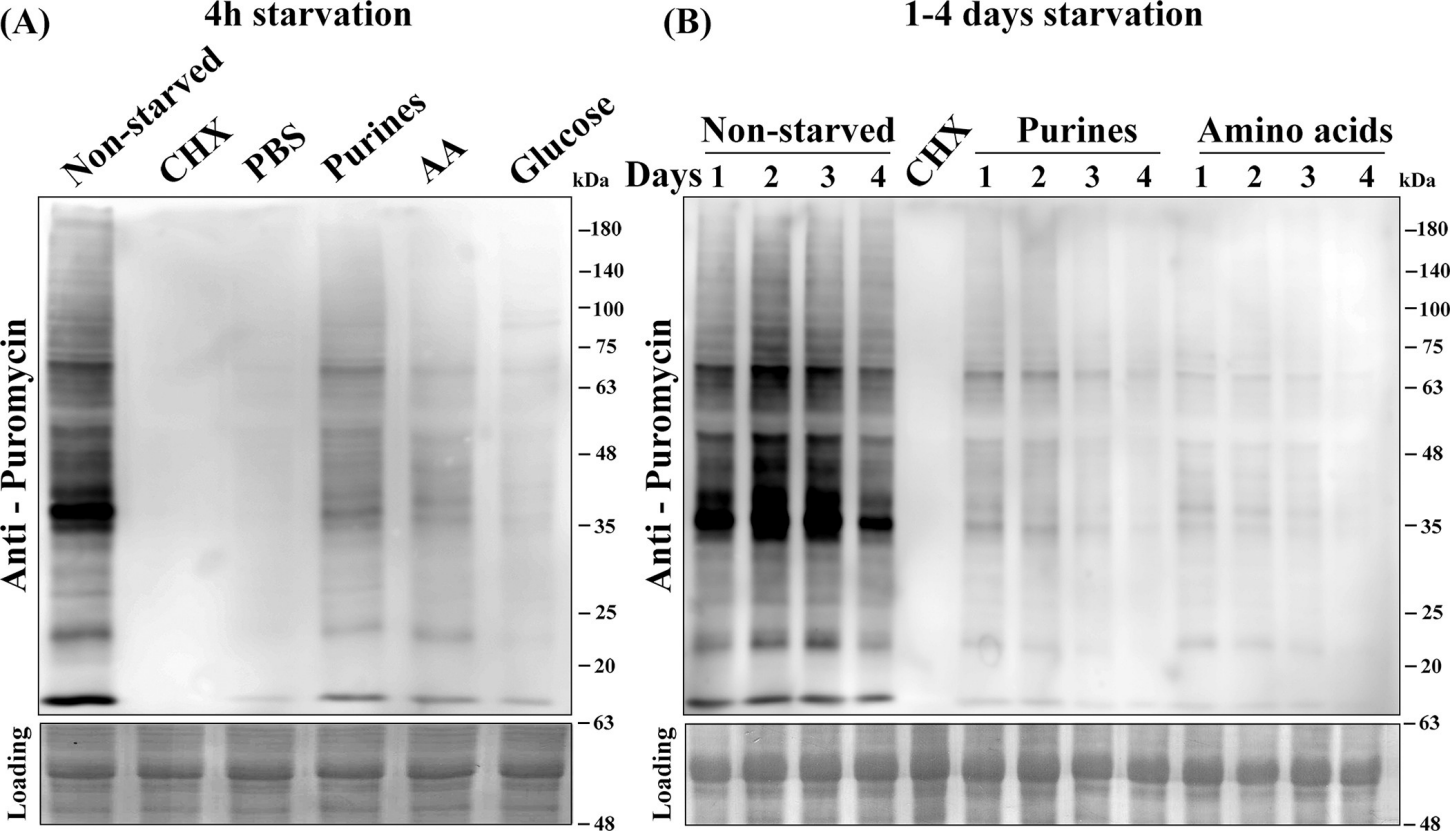
Inhibitory effect of depleting specific nutrients on translation. Wild type *L*. *amazonensis* cells were exposed to specific nutrient deprivation conditions [PBS, purines, amino acids [31] and glucose] for 4 h. A cycloheximide (CHX) control for translation inhibition is also shown **(A).** The effects of depriving purines and AA were measured over longer time periods extending up to 4 days **(B).** Cells were then incubated with 1 μg/ml puromycin for 20 min, extracted, separated over 12% SDS-PAGE and subjected to western analysis using specific antibodies against puromycin. The bottom lane shows the Ponceau stain of the blot, to verify equal gel loads.

The absence of specific nutrients occurs already in the sand fly, and is an integral part of the *Leishmania* life cycle. We, therefore, examined the effects of eliminating specific nutritional components on global protein synthesis. Active translation was followed using the 'surface sensing of translation' (SUnSET) technology, which is based on monitoring the incorporation of puromycin into *de novo* synthesized polypeptides [44]. *L*. *amazonensis* promastigotes were incubated in nutrient-free PBS, or in media deficient of glucose for 4 h, whereas removal of purines or amino acids was performed during longer time periods. The starved cells were then incubated with puromycin for 20 min and *de novo* translation was monitored on western blots using anti-puromycin antibodies. As shown in Fig 1A, translation was reduced already after 4 h of nutrient depletion, as compared to actively translating non-starved cells. The strongest effect was observed in cells incubated in PBS or cells depleted of glucose, as compared to the complete arrest observed in cells incubated with cycloheximide. The effects of eliminating purines and amino acids for longer periods ranging between 1–4 days (no cells survived longer periods of glucose depletion) were also examined (Fig 1B). Depletion of purines and amino acids reduced translation as early as after 4 h, and the effect became more pronounced with time. The presence or absence of dialyzed fetal calf serum did not change the inhibitory effect (S1A Fig), indicating that the absence of purines or amino acids was the cause of the reduced translation. The western blots were all quantified by densitometry (S1B Fig).

### Nutrient deprivation targets LeishIF4E-3 into cytoplasmic granules

**Fig 2:**
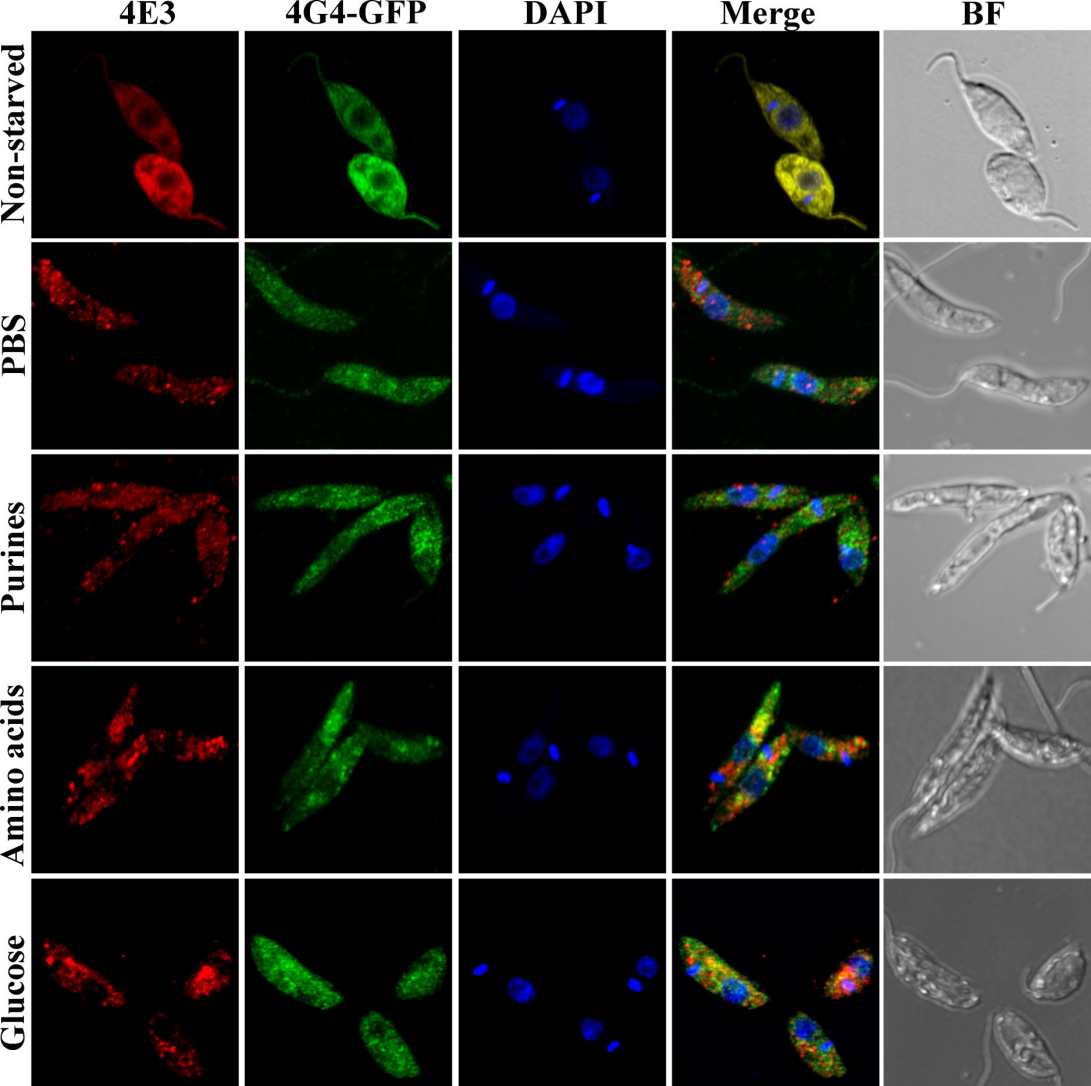
Cytoplasmic distribution of LeishIF4E-3 and LeishIF4G-4 in response to eliminating different nutritional components. *L*. *amazonensis* cells expressing LeishIF4G4-GFP were starved for 4 h either completely (PBS) or specifically to purines, amino acids or glucose. The cells were then washed, fixed, permeabilized and processed for confocal microscopy. LeishIF4E-3 was detected using rabbit anti-LeishIF4E-3 antibodies and a secondary fluorescent antibody (550 nm; red). LeishIF4G-4 was visualized through its fusion to GFP (488 nm; green). Nuclear and kinetoplast DNA was stained using DAPI (blue). A bright field (BF) picture of the cells is provided in the right column.

Impaired translation often leads to the formation of cytoplasmic RNA granules [20]. We previously examined granules induced by nutritional stress and showed that incubation in PBS caused LeishIF4E-3 to concentrate in specific cytoplasmic foci [34]. In view of the role played by purine starvation during metacyclogenesis pathways, we tested the effects of depleting purines, as well as other specific nutrients, on the formation of LeishIF4E-3-containing granules. In addition, the intracellular localization of LeishIF4G-4 was examined in a transgenic cell line expressing GFP-tagged LeishIF4G-4, thus allowing for parallel visualization of both proteins. Fig 2 and S2A Fig (for the broad field) show that under normal growth conditions, LeishIF4E-3 was dispersed throughout the cytoplasm, co-localizing with LeishIF4G-4. However, exposing the cells to different types of nutritional depletion caused LeishIF4E-3 to concentrate in specific cytoplasmic foci as early as 4 h after the onset of the stress. LeishIF4G-4 was not detected in the granules formed following purine starvation (see the merge panel in Fig 2). We excluded the possibility that granule formation could be affected by the high level of episome-derived expression of SBP-tagged LeishIF4E-3, since starvation-induced LeishIF4E-3 containing granules were observed when wild type parasites were subjected to nutritional stress but not under normal conditions [S2B Fig and S2C Fig (broad field)].

To analyze the dynamic nature of these granules, we tested whether they disassembled upon recovery from nutritional depletion. Starved parasites (4 h) were replenished with complete medium containing all the supplements required for normal growth, and were allowed to grow for 24 h. Confocal microscopy using antibodies against LeishIF4E-3 monitored the abundance of LeishIF4E-3-containing granules. Notably, following recovery from nutritional stress, the starvation-induced LeishIF4E-3 containing granules disappeared [S2D Fig and S2E Fig (broad field)]. Recovery was also observed if the cells were starved for purines during 4 days (with or without dialyzed FCS, S2F Fig). The granules disappeared after the cells returned to complete media for 24 h with and without dialyzed FCS [S2F Fig and S2G Fig (broad field)]. Thus, the absence of dialyzed FCS did not prevent recovery of the cells from the presence of LeishIF4E-3 containing granules.

### Co-localization of LeishIF4E-3-containing cytoplasmic granules and *HSP83* mRNA

**Fig 3:**
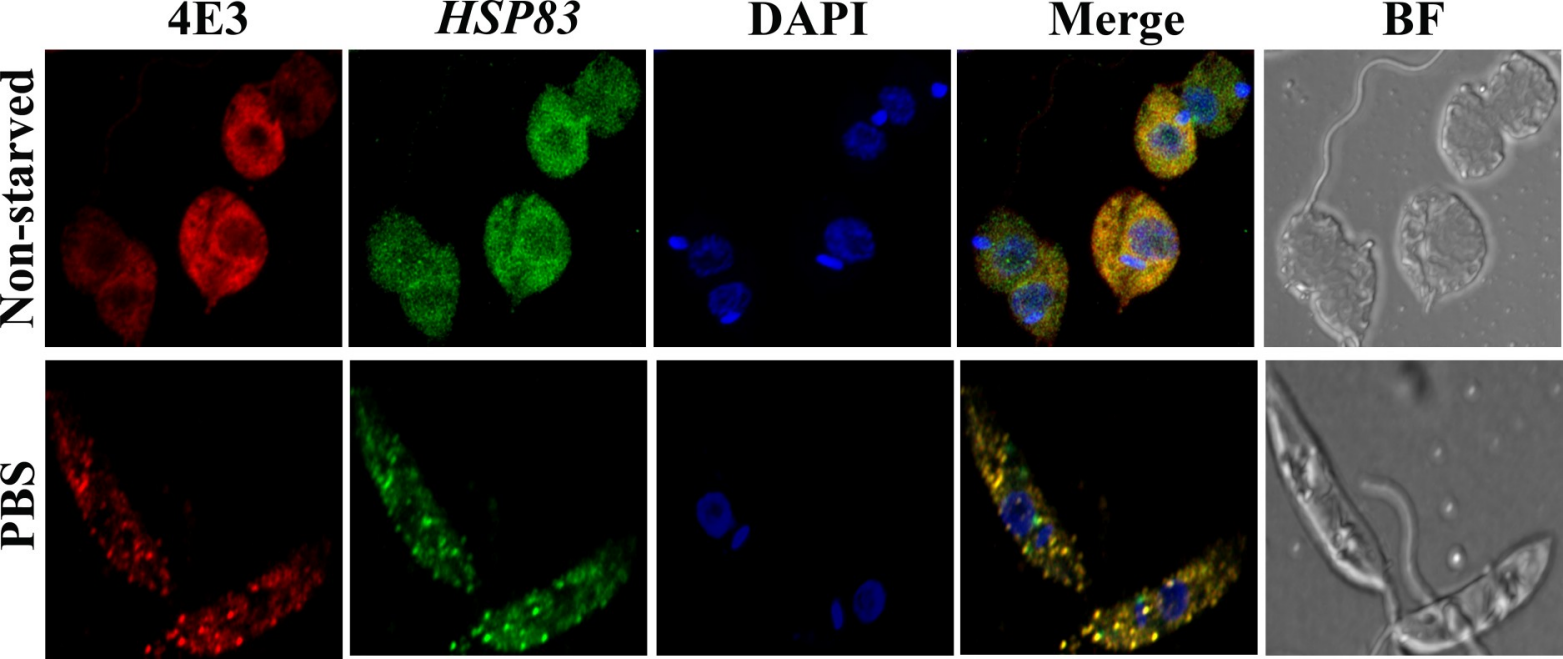
LeishIF4E-3 starvation-induced granules contain *HSP83* mRNA. Wild type *L*. *amazonensis* cells were starved in PBS for 12 h, fixed, permeabilized and processed for mRNA FISH analysis. mRNA was stained by fluorescence *in situ* hybridization using a DIG-labeled probe derived from *HSP83* mRNA. Hybridization was detected using FITC-labeled antibodies against DIG (488 nm, green). LeishIF4E-3 was detected using rabbit anti-LeishIF4E-3 antibodies as described in the legend to Fig 2 (red). Nuclear and kinetoplast DNA was stained using DAPI (blue) and a bright field (BF) picture of the cells is shown.

The mechanism that targets LeishIF4E-3 to starvation-induced cytoplasmic granules is poorly understood. One possibility is that LeishIF4E-3 escorts mRNAs for storage in granules, under conditions whereby translation is arrested due to nutrient deficiencies. Alternatively, if stalled ribosomes are targeted to these granules, associated mRNAs would accompany them. We, therefore, examined whether the starvation-induced LeishIF4E-3 containing granules indeed contain mRNA. Non-starved and starved parasites were examined by fluorescence *in situ* hybridization (FISH) analysis of mRNA, using DIG-labelled probes derived from *HSP83*. While non-starved parasites showed homogeneous cytoplasmic distribution of the mRNA, following nutritional depletion (12 h), their staining was enriched within the starvation-induced LeishIF4E-3 containing granules [Fig 3 and S3A Fig (broad field)]. The granules contained mature mRNAs, as no hybridization was observed with probes directed against the *HSP83* intergenic region [IR, positions 891–1118, S3B Fig and S3C Fig (broad field)]. Finally, we cannot exclude the possibility that LeishIF4E-3 and the *HSP83* transcript simply co-migrate into the granules, as we do not show a direct interaction between them. However, since the LeishIF4E-3 complex contains RNA binding proteins, these could be involved in targeting the *HSP83* mRNA to the cytoplasmic granules, along with LeishIF4E-3.

### LeishIF4E-3 is differentially phosphorylated in response to different nutrient deprivation

**Fig 4:**
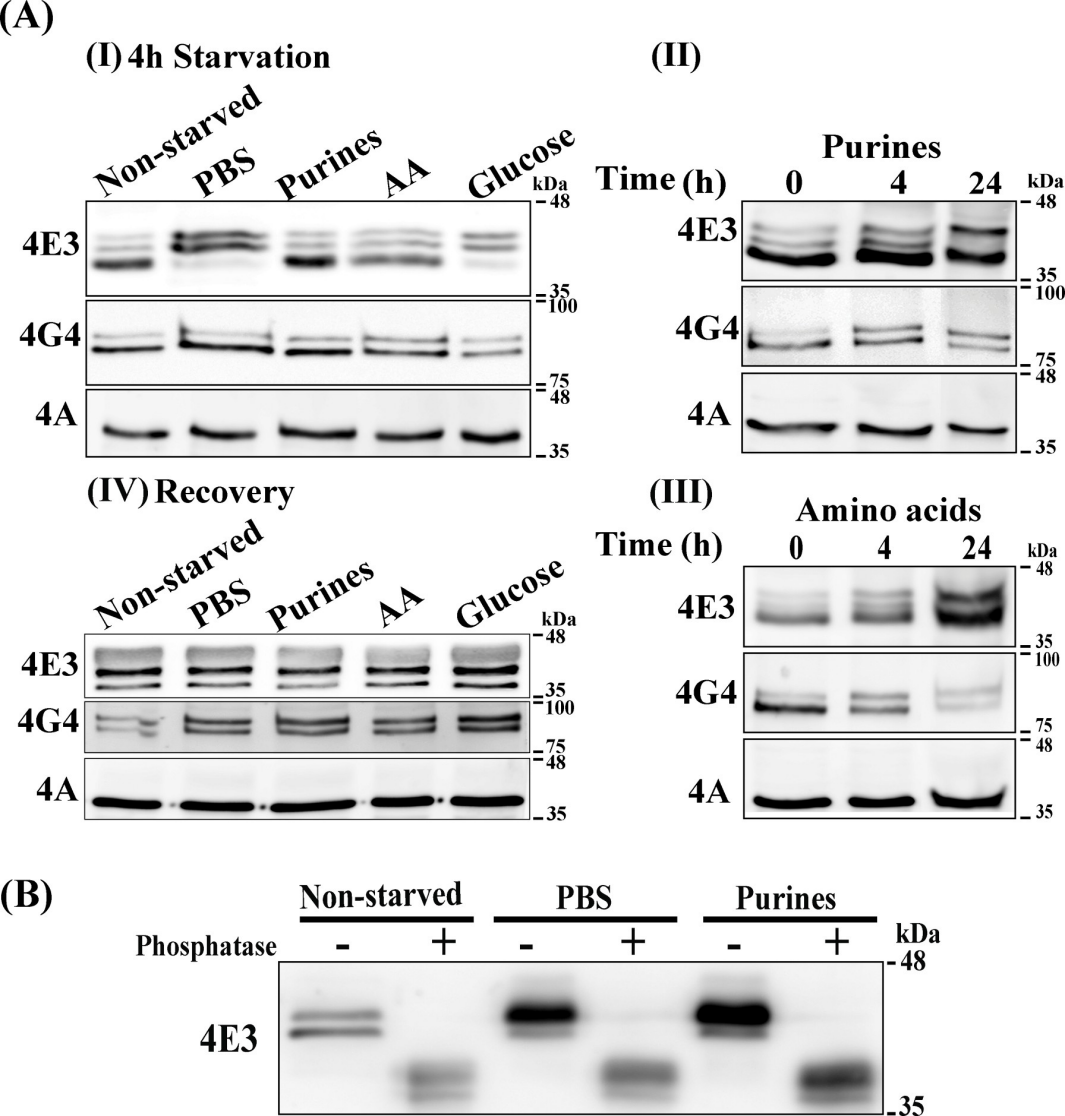
Changes in the migration profile of LeishIF4E-3 during different starvation conditions. **(A)** Wild type *L*. *amazonensis* promastigotes were grown in nutrient-free buffer (PBS) or in medium lacking purines, amino acids or glucose for 4 h **(I)**. Cells were starved for purines (**II)** or amino acids **(III)** during a 24 h window. Following a starvation period of 4 h, stressed parasites were allowed to recover in DMEM medium supplemented as described in Materials and Methods for 24 h **(IV)**. Total cellular extracts were resolved on reduced bis-acrylamide SDS-PAGE and subjected to western analysis using specific antibodies against LeishIF4E-3, LeishIF4G-4 or LeishIF4A. LeishIF4A levels remained unchanged during nutritional stress and, therefore, served as loading control. **(B)** λ**-**phosphatase treatment showing the effect of different starvation treatments on phosphorylation of LeishIF4E-3. Wild type *L*. *amazonensis* promastigotes were incubated in the absence of all nutrients (PBS) for 4 h or incubated in the absence of purines for 4 days. A non-starved parasite culture was used as control. Total cellular extracts were treated with λ**-** phosphatase for 30 min. Treated cellular lysates were resolved on 12% reduced bis-acrylamide SDS-PAGE and gels were subjected to western analysis using specific antibodies against LeishIF4E-3.

Western analysis of protein lysates from starved and non-starved parasites demonstrated changes in the migration profile of LeishIF4E-3 following specific nutrient starvation (4 h), and highlighting a shift to slower migrating isoforms [Fig 4A (I–III)]. All westerns of Fig 4A were subjected to densitometry quantification (S4A Fig). The shift in migration was observed even after a shorter 1 h incubation, with or without dialyzed FCS (S4B Fig). The effects of depleting purines and amino acids were observed only after 24 h [Fig 4A (II)] and continued for 4 days (S4C Fig). Following recovery from nutritional stress obtained by incubation of the parasites in fresh promastigote growth medium for 24 h, LeishIF4E-3 lost its differential migration pattern, as no change in the migration pattern of LeishIF4E-3 was observed, in comparison to non-starved parasites [Fig 4A (IV)].

To verify that the LeishIF4E-3 modification during starvation originated from phosphorylation, protein lysates from starved (4h in PBS and 4 days for purine starvation) and non-starved parasites were treated with λ-phosphatase and further analyzed in western blots. Fig 4B shows that following the phosphatase treatment, the LeishIF4E-3 once again resumed its original fast migrating pattern, reflecting the non-phosphorylated form of the protein.

### Identification of the LeishIF4E-3 phosphorylation site and mutational analysis

The phosphorylation site of LeishIF4E-3 following nutritional deprivation was identified using LC-MS/MS, performed with and without enrichment for the phospho-peptides. In both cases, a single phosphorylated peptide was identified in the enriched fraction of phospho-peptides, highlighting that phosphorylation occurred at position S75, which is part of the extended amino terminus of LeishIF4E-3 (S1 Table and S4D Fig). Peptides covering positions 84 and 105, which are known to be phosphorylated in *L*. *infantum* [3], were not phosphorylated in *L*. *amazonensis* LeishIF4E-3. The *T*. *brucei* phosphorylation sites [45, 46] are not conserved with *L*. *amazonensis*, or *L*. *infantum* (S4D Fig). The percentage of the phosphorylated peptide in the cell extract was 74% in cells starved in PBS, whereas only 15% of this peptide were phosphorylated in non-starved cells (S1 Table).

**Fig 5:**
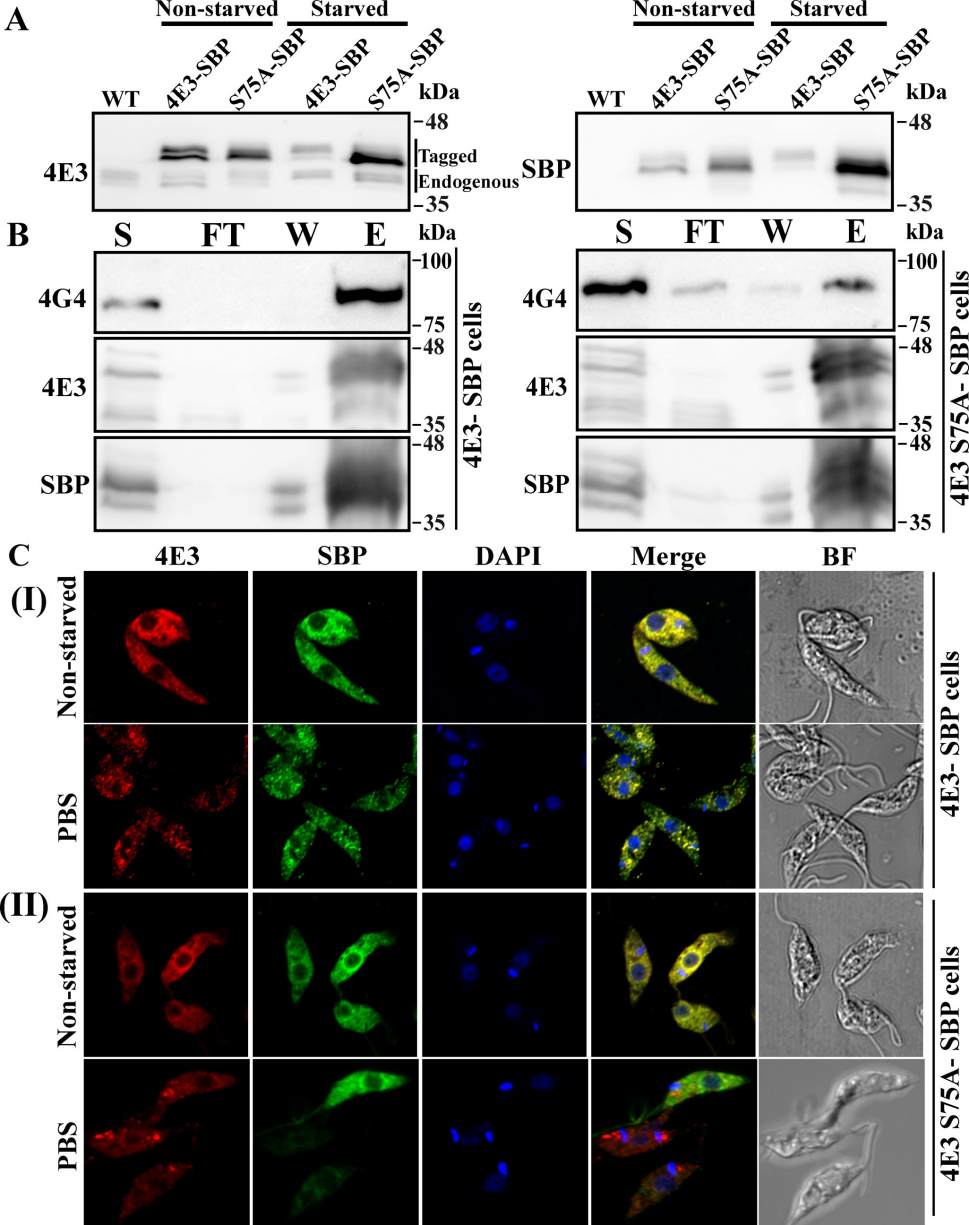
The S75A mutation of LeishIF4E3 leads to a decrease in granule formation in response to PBS starvation and to a reduced interaction with LeishIF4G-4. **(A)** Migration profile of the endogenous and tagged LeishIF4E-3 on SDS-PAGE under non-starved and starved conditions. Transgenic *L*. *amazonensis* promastigotes expressing either SBP-tagged LeishIF4E-3 or the S75A SBP-tagged mutant LeishIF4E3 were grown in complete DMEM or in nutrient-free buffer (PBS) for 4 h. Total cellular extracts were resolved on reduced bis-acrylamide SDS-PAGE and subjected to western analysis using specific antibodies against LeishIF4E-3, or against SBP tag. A non-starved parasite culture was used as control. **(B) Co-purification of LeishIF4G-4 with SBP-tagged LeishIF4E-3 and S75A mutant LeishIF4E-3 under normal conditions.** Non-starved parasites expressing either SBP-tagged LeishIF4E-3 or the S75A mutant LeishIF4E-3 were subjected to pull-down analysis over streptavidin-Sepharose beads. The eluted complexes were separated over 12% SDS-PAGE that were further subjected to western analysis using specific antibodies against LeishIF4E-3 or LeishIF4G-4. The gels were loaded with samples taken from the total supernatant prior to the pull down (S, 2%), the flow through fraction (FT, 2%), the final wash (W, 50%) and the eluted fraction (E, 50%). **(C) Confocal analysis of SBP-tagged LeishIF4E-3 (I), or SBP-tagged S75A mutant LeishIF4E3 ((II), starved or non-starved.** The cells were fixed, permeabilized and processed for confocal microscopy. LeishIF4E-3 was detected using rabbit anti-LeishIF4E-3 antibodies followed by incubation with anti-rabbit DyLight-labeled secondary antibodies (550 nm; red). Mutant SBP-tagged S75A LeishIF4E-3 was visualized using mouse monoclonal antibodies against SBP followed by incubation with anti-mouse DyLight-labeled secondary antibodies (488 nm; green). Nuclear and kinetoplast DNA was stained using DAPI (blue). Bright field pictures are shown on the right.

To test the relevance of the serine 75 phosphorylation for the ability of LeishIF4E-3 to form granules, a LeishIF4E-3 (S75A) phospho-mutant was overexpressed in cells via stable transfection. Fig 5A shows the migration profile of the endogenous and mutant LeishIF4E-3 polypeptides. Using the antibodies against the SBP tag shows that the slower migrating band of the mutant protein was reduced, as compared to the wild type tagged protein (S5A Fig). However, a faint band above the main interacting protein was still observed, and could be assigned to other modifications of the mutant S75A LeishIF4E-3 (carbamidomethylation and oxidation), which were identified in the mass spectrometry analysis (S1 Table). Fig 5B shows that the S75A mutation of the phosphorylation site also reduced the interaction between LeishIF4E-3 and LeishIF4G-4 (Fig 5B and S5B Fig). Finally, the S75A mutation also caused a reduction in the ability of the mutated LeishIF4E-3 to granulate in response to a nutritional stress, as observed in Fig 5C and S5C Fig, whereas the formation of granules containing native LeishIF4E-3 was not interrupted.

### Enrichment of LeishIF4E-3-associated proteins from the dense sucrose fractions.

**Fig 6:**
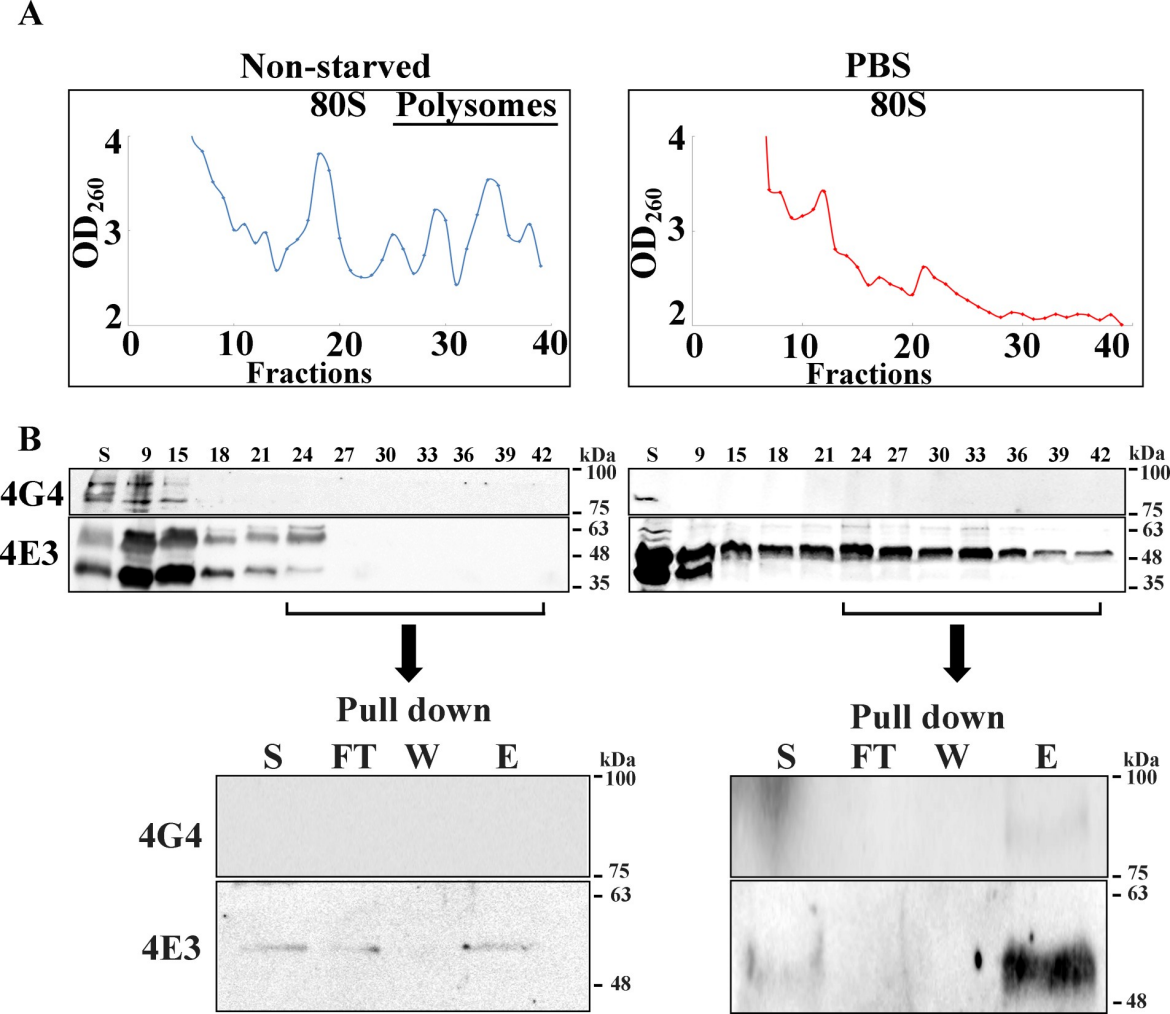
Enrichment of starvation-induced LeishIF4E-3-containing granules over sucrose gradients. Transgenic *L*. *amazonensis* promastigotes expressing SBP-tagged LeishIF4E-3 were fully starved (PBS, right panel) or kept under normal conditions as controls (left panel). **(A)** Cell extracts were treated with cycloheximide (100 μg/ml) followed by fractionation over 10–40% sucrose gradients. The OD_260_ of the sucrose fractions is shown in the top panels. **(B)** Samples from the fractionated proteins were precipitated by TCA and further resolved over 12% SDS-PAGE. The migration profile of the proteins was shown by western analysis using specific antibodies against LeishIF4E-3 or LeishIF4G-4. The gels were loaded with 15 μl from the total supernatant fraction (S, 0.75%) and 15 μl from each fraction (fraction number, 5%). Fractions 25–42 were pooled, and further pulled-down over streptavidin-Sepharose beads. The eluted complexes were analyzed in western blots using specific antibodies against LeishIF4E-3 or LeishIF4G-4. The gels were loaded with a sample of the pooled fractions prior to the pull down (S, 10%) and from the flow through fraction (FT, 10%), followed by a sample of the last wash (W, 50%) and the eluted fraction (E, 50%). Similar results were obtained from three independent experiments.

The proteomic content of the dense sucrose fractions following pull down by LeishIF4E-3 was analyzed. These fractions could represent the newly formed starvation-induced granules. Extracts of exponentially growing cells that expressed the tagged LeishIF4E-3 were fractionated over sucrose gradients and the heavy fractions were pooled. The OD_260_ profile is given in Fig 6A. The western analysis of the gradient fractions that is shown in Fig 6B (middle panel) further highlights that starvation conditions eliminated the polysomes. The western analysis of the different fractions shows that starvation conditions caused LeishIF4E-3 to migrate in the heavy fractions. These were pooled for further treatment. Parallel fractions from non-starved control cells contained only a small amount of LeishIF4E-3, as expected, since under normal growth conditions LeishIF4E-3 is not abundant in the heavy fractions. The pooled heavy fractions from extracts of stressed cells were affinity-purified over streptavidin beads and the presence of LeishIF4E-3 in the eluted fractions was verified by western analysis (Fig 6 bottom panel and S6A Fig). A control assay verified that the addition of cycloheximide to the cells prior to granule purification did not prevent the formation of granules during starvation. This was verified by confocal microscopy of starved and non-starved cells that were incubated with cycloheximide [S6B Fig and S6C Fig (broad field)]. In accordance, LeishIF4E-3 migrated in the heavy fractions of the gradient in the absence of cycloheximide (S6D Fig).

**Fig 7:**
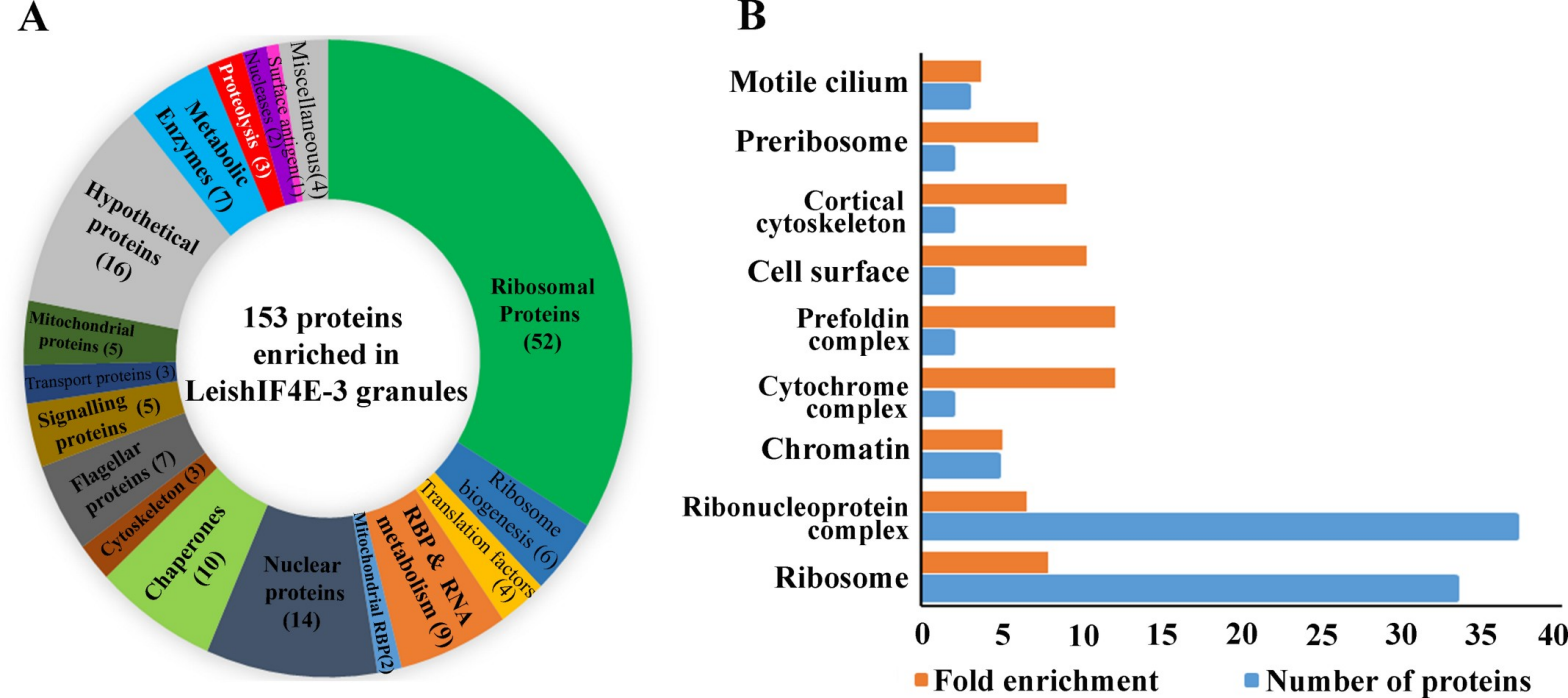
Categorized proteomic content of the starvation-induced LeishIF4E-3 containing granules. The proteomic content of starvation-induced LeishIF4E-3 containing granules enriched over sucrose gradients and further pulled-down over streptavidin-Sepharose beads was determined by LC-MS/MS analysis, in triplicates and compared to a control pull down with a non-relevant protein. Parallel control cells expressing SBP-tagged luciferase were treated similarly and subjected to LC-MS/MS analysis, in triplicates and in the same run. The proteins were identified by the MaxQuant software using TriTrypDB database annotations. Differences between the proteomic contents of the LeishIF4E-3 and luciferase pulled-down fractions were determined using the Perseus statistical tool. Proteins enriched two fold with a p<0.05 were categorized according to their function. **(A)** Relative distribution of proteins that were manually categorized into functional groups. The pie chart shows the relative abundance of each category, based on the summed intensities of the peptides that were used to identify the individual proteins. The numbers in brackets in the pie chart represent the number of proteins in each category. Hypothetical proteins and proteins with non-defined functions are in grey, while proteins with known molecular functions are differently colored. **(B)** GO term enrichment by cellular components. The resulting GO terms were enriched by at least four fold as compared to the gene sets encoded in the genome, with p< 0.01.

The protein complexes that were eluted from the heavy fractions following pull-down of SBP-tagged LeishIF4E-3 were subjected to LC-MS/MS analysis. To exclude non-specific pulled-down products, we employed the same protocol with a control *Leishmania* cell line that over-expressed the luciferase reporter gene, to generate a non-related protein that was also SBP-tagged. Extracts of cells expressing SBP-tagged luciferase were separated over sucrose gradients, the heavy fractions were pooled and pulled-down via the SBP tag using streptavidin beads (S6E Fig). The eluted material was analyzed by LC-MS/MS in parallel to the LeishIF4E-3 pulled-down products. Each pull-down experiment was performed in triplicates and all samples were analyzed in the same run. The peptides that were pulled down from SBP-tagged LeishIF4E-3 and from SBP-tagged luciferase fractions were identified using the MaxQuant software. The Perseus statistical analysis highlights the proteins that were significantly enriched by at least two fold in the LeishIF4E-3 pulled-down assay, as compared to the luciferase control pulled-down experiment, with p<0.05. This comparison was based on average intensities, calculated from three similar but independent experiments. The proteins enriched in the LeishIF4E-3 containing granules were manually categorized into functional groups, as shown in Fig 7A and S2 Table. The manual classification highlighted the presence of multiple ribosomal proteins, along with RNA binding proteins, chaperones, signaling proteins and metabolic enzymes. The proteins were also analyzed for their GO term enrichment, using TriTrypDB; using a threshold of four fold enrichment as compared to the gene sets encoded in the genome, with a p< 0.01.

Results are shown in Fig 7B and S3 Table. The GO term analysis also highlighted the enrichment of a large number of ribosomal proteins, along with ribonucleoproteins, pre-ribosomal proteins and proteins involved in ribosome maturation. Altogether, our observations suggest that the granules could serve for storage of ribosomes and ribosomal subunits that were stalled due to the nutritional stress.

The LeishIF4E-3 pulled-down granules also contained RNA helicases and RNA-binding proteins, in line with the potential role of these granules in RNA storage. The presence of a variety of metabolic enzymes is intriguing, but supported by a recent report indicating their ability to bind RNA, despite the absence of canonical RNA recognition motifs [47, 48]. With respect to translation, while most initiation factors were not detected, the granules contained factors related to the elongation process, namely LeishEF1b and LeishIF5a. The latter has been implicated in elongation, rather than in initiation of translation [49]. The enriched fractions also contained LeishPABP2, but not LeishPABP1, which is part of the LeishIF4E-4 canonical complex [29, 50]. The trypanosomatid ortholog TbPABP2 is also found in stress granules in *T*. *brucei* [51, 52]. It is important to note that RNA degradation enzymes were not highlighted in the LeishIF4E-3 pulled-down granules. This distinguishes the LeishIF4E-3 pulled-down granules from the nutritional stress-induced granules of *T*. *brucei*, which were purified using a different approach [26]. Another notable difference is that classical P-body markers, such as DHH1 and Scd6, were not found in the LeishIF4E-3 granule proteome. The absence of LeishIF4G-4 among the significantly enriched granular proteins is in line with our former report that nutritional stress disrupted the interaction between LeishIF4E-3 and LeishIF4G-4 [34]. As expected, the starvation-induced LeishIF4E-3 containing granules contained several chaperones, which usually accompany proteins that are over-expressed in cells. The presence of a few signaling proteins is not surprising, as are the few proteins related to the cytoskeleton. However, the presence of a larger repertoire of nuclear proteins is intriguing.

**Fig 8:**
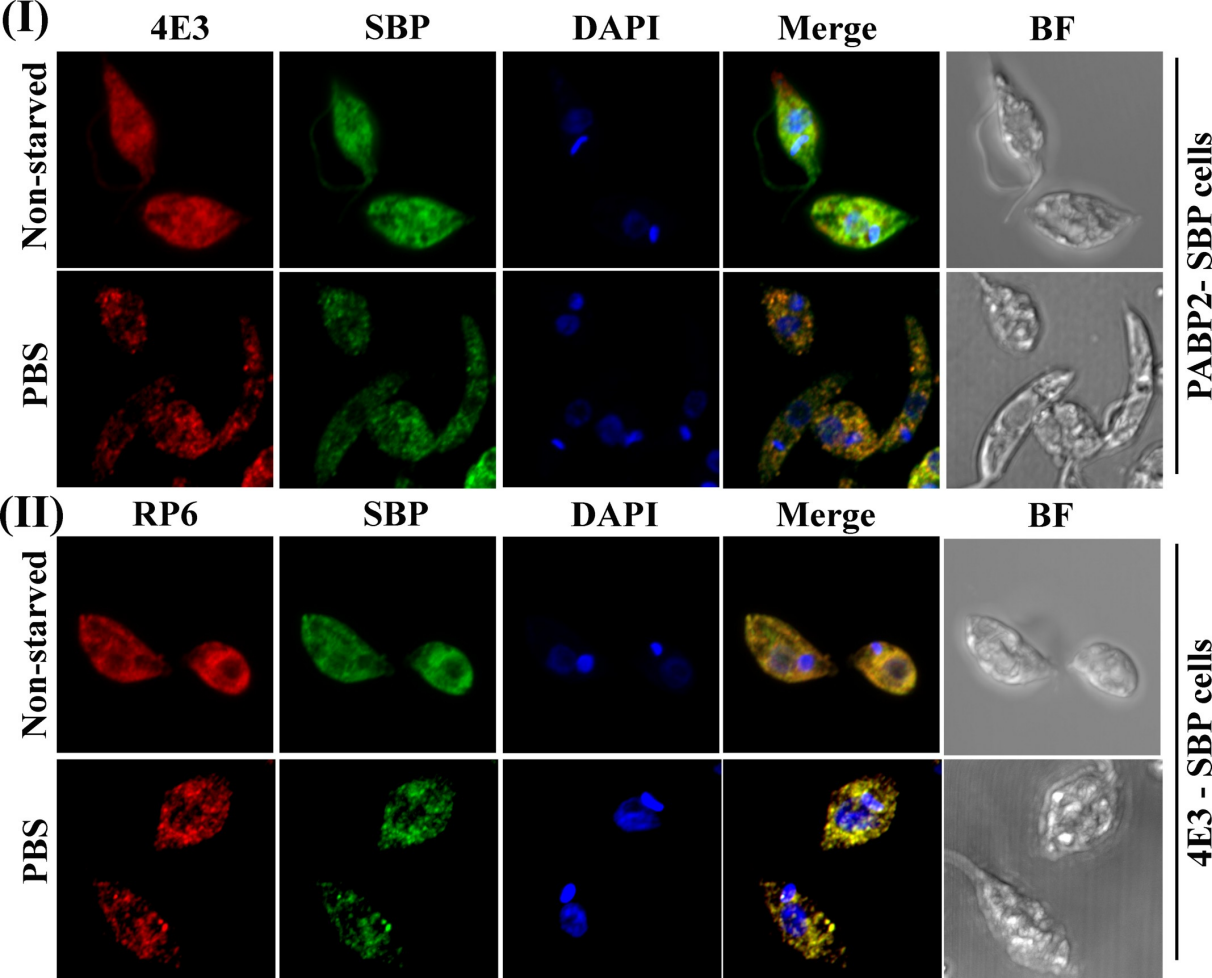
LeishPABP2 and LeishRPS6 co-localize with starvation-induced LeishIF4E-3 containing granules following nutrient deprivation. Transgenic *L*. *amazonensis* promastigotes expressing either SBP-tagged LeishPABP2 **(I)** or SBP-tagged LeishIF4E-3 **(II)** were subjected to nutrient starvation (PBS) for 4 h. The cells were then fixed, permeabilized and processed for confocal microscopy. **(I)** LeishIF4E-3 was immuno-stained with specific rabbit antibodies and secondary DyLight-labeled anti-rabbit antibodies (550 nm; red). LeishPABP2 was detected using mouse monoclonal antibodies against SBP and secondary anti-mouse DyLight antibodies (488 nm; green). **(II)** RPS6 was detected using specific rabbit antibodies and secondary DyLight anti-rabbit antibodies (550 nm; red). SBP-tagged LeishIF4E-3 was visualized with mouse monoclonal anti-SBP antibodies and detected with DyLight-labeled anti-mouse secondary antibodies (488 nm; green). Nuclear and kinetoplast DNA was stained using DAPI (blue). Bright field pictures are shown on the right.

Using confocal microscopy, we further validated the co-localization of two proteins that were identified in the LeishIF4E-3 starvation granule, namely LeishPABP2 and the *Leishmania* ribosomal protein S6 (RPS6) [Fig 8 (I) and (II) and S7 Fig]. LeishPABP2 was tagged with SBP and its localization was monitored using anti-SBP monoclonal antibodies. LeishIF4E-3 was stained by rabbit-raised specific antibodies. Fig 8 shows that the two proteins co-localize, at least on a partial basis, which is not surprising, since LeishPABP2 could participate in multiple complexes on top of the granules. For testing co-localization of RPS6 and LeishIF4E-3 during a starvation stress, the protein was stained with rabbit antibodies against RPS6, LeishIF4E-3 was monitored using mouse anti-SBP monoclonal antibodies. The results verify the presence of ribosomal proteins in the starvation-induced LeishIF4E-3 containing granules. Thus, the confocal analysis suggests both RPS6 and PABP2 co-localize, at least partially, with LeishIF4E-3 in the same nutritional stress-induced granules.

### Nutritional stress affects parasite growth, viability and motility *in vitro*

**Fig 9:**
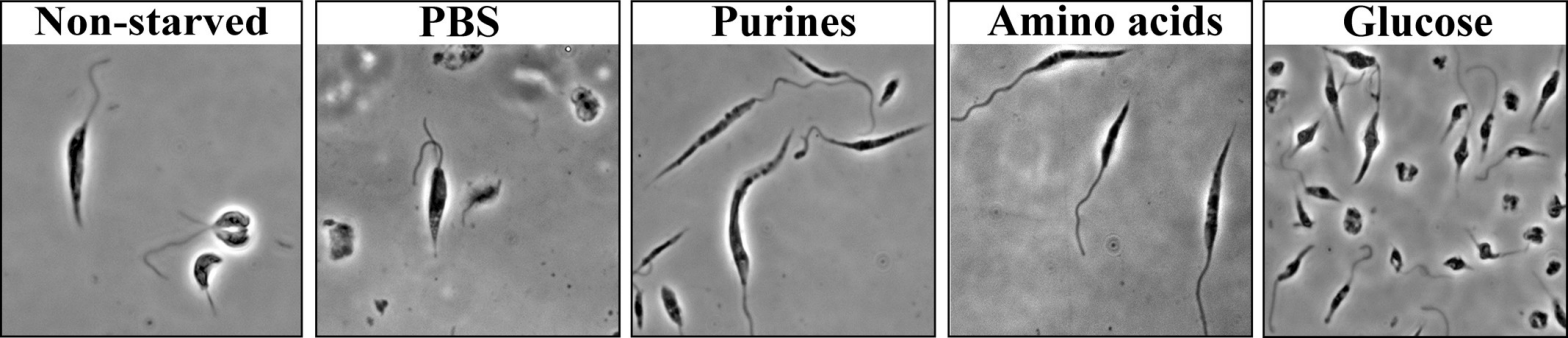
Nutrient deprivation of *L*. *amazonensis* in culture affects parasite morphology. *L*. *amazonensis* promastigotes were grown in DMEM containing all supplements in control experiments. Cells were starved upon incubation in PBS for 24 h, or grown in DMEM lacking purines, amino acids or glucose for the same period, with FCS that was pre-dialyzed to remove low molecular weight materials. The cells were fixed and visualized by phase contrast microscopy at 100x magnification. Phase contrast images of parasites were captured with a Zeiss Axiovert 200M fluorescence microscope and photographs were obtained using an AxioCam HRm CCD camera.

The effects of specific nutrient stresses on parasite growth and viability were monitored following the depletion of purines, essential amino acids or glucose. S8 Fig shows that while removal of each nutrient arrested cell proliferation, their effects on cell viability varied. While prolonged deprivation of either glucose or amino acids was lethal to the cells, the absence of purines over the same period did not reduce viability. Furthermore, the deprivation of each nutrient had a different effect on cell morphology (Fig 9). Eliminating purines as well as amino acids induced the appearance of slender-shaped cells with a long flagellum, which are typical of nectomonad cells, a form that precedes differentiation into metacyclic cells [53]. Glucose deprivation resulted in the appearance of "fat" cells, which are usually associated with cell lethality. Starvation for purines and amino acids maintained and even increased parasite motility (S1–S5 Videos). This mimics events in the fly, where starved parasites leave the hindgut and migrate to the front parts of the mouth [53].

## Discussion

In this paper, we report a direct correlation between the availability of nutrients on translation, post translational modifications of LeishIF4E-3 and on morphological changes. We further propose that the parasites may have developed temporary means to store inactive mRNAs and stalled ribosomes in dedicated granules that also contain LeishIF4E-3, possibly allowing their recycling upon renewal of nutrient availability.

In trypanosomes, TbIF4E-3 is an essential protein, which could support its role in active translation. The *Leishmania* ortholog, LeishIF4E-3, could be assigned a similar role, although it binds the mRNA cap structure rather inefficiently as compared to other LeishIF4Es [54]. Indeed, one of the three key aromatic residues in its cap-binding pocket is substituted by methionine, possibly accounting for its weak cap-binding activity [30]. We, therefore, searched for additional functions for LeishIF4E-3 that could coincide with being part of storage bodies for RNA, and possibly inactive ribosomes.

Leishmanias and trypanosomes are known to harbor a multitude of RNA granules, which are formed in response to different stresses [19, 21, 55]. We previously reported that during nutritional stress, LeishIF4E-3 concentrated in specific granules [34], but the content of these granules was not resolved. We verify here the presence of mature mRNAs in these starvation-induced LeishIF4E-3 containing granules, in line with our hypothesis that these granules serve as storage bodies for mature mRNAs, such as the *HSP83* transcript. However, it is not clear whether the *HSP83* mRNA concentrates in the granules through a direct association with LeishIF4E-3, or through other RNA-binding proteins that were identified in our proteomic analysis. A partial co-localization of LeishIF4G-4 with the starvation-induced LeishIF4E-3 containing granules was observed, but mostly in cells starved for amino acids and glucose, and not in cells fully starved (PBS) or starved for purines.

We further characterize the nutritional stress that led to the formation of LeishIF4E-3 containing granules. Depleting purines, amino acids or glucose led to a decrease in translation (with variable kinetics), despite previous reports that mRNA abundance is not altered during purine starvation [11]. However, removal of each nutrient had a different effect on cell viability and morphology. Depletion of purines generated elongated slender cells with a long flagellum that maintained their viability and motility, whereas depletion of glucose generated "fat cells" that died within 24 h. The effect of depleting glucose for short periods (4 h) could be reversed, while longer incubations were lethal. Unlike glucose depletion, elimination of purines for a relatively long time (4 days) did not lead to cell death and the cells could easily recover. During recovery, the LeishIF4E-3 containing granules disappeared, and the protein resumed its homogenous cytosolic distribution.

In attempt to examine the nature of the LeishIF4E-3 containing foci following a nutritional stress, we separated extracts of cells expression the SBP-tagged LeishIF4E-3 over sucrose gradients and isolated the dense fractions. These could include the LeishIF4E-3 containing granules, since the non-granulated LeishIF4E-3 remained in the top fractions. A subsequent pull-down step over streptavidin beads enriched the fraction of the putative starvation-induced LeishIF4E-3 containing granules, which was subjected to LC-MS/MS analysis. Parallel control cultures expressing SBP-tagged luciferase were treated and processed similarly, and analyzed in the same run, to control for non-specific binding to the streptavidin-Sepharose beads. The statistical analysis identified a significant increase of the bait protein, LeishIF4E-3, along with a large group of ribosomal proteins. Co-localization of the ribosomal protein RPS6 in the LeishIF4E-3 containing granules was verified by confocal microscopy. Although a potential contamination of ribosomal proteins could be attributed to residual polysomes in these dense fractions, these were not enriched in the control experiment with the SBP-luciferase expressing cells. Thus, enrichment of ribosomal proteins in these granules can account for the mechanism by which the parasites store stalled ribosomes.

LeishPABP2 was identified in the LeishIF4E-3 containing granules, and further verified by confocal microscopy. The PABP2 ortholog was also found in stress granules of trypanosomes [26, 51]. In response to transcription inhibition by the addition of Actinomycin D [18], LeishPABP2 and LeishPABP3 were also shown to migrate to the nucleus. It is interesting to note that the LeishIF4E-3 containing granules did not contain other translation initiation factors, except for two elongation factors, LeishEF1b and LeishIF5a, with the latter having been shown to be involved in elongation rather than in initiation [49]. The possibility that stress granules are active in translation elongation but not in initiation was recently proposed [15]. Although we have no evidence that translation elongation is performed in these granules, the significant enrichment of an elongation factor in the granules could support the possibility that a limited elongation process occurred within such bodies. Finally, enrichment with ribosomal proteins was not reported in *T*. *brucei*, excluding the possibility that translation elongation takes place in the *T*. *brucei* granules [26].

The presence of ribosomal proteins in the LeishIF4E-3 containing granules were also identified through the GO term enrichment analysis, along with several RNA helicases and RNA-binding proteins that usually accompany RNA molecules. The *Leishmania* and *Trypanosoma* granules [26] share several RNA-binding proteins, such as DRB2 and the Lupus La protein. La is an RNA chaperone that can promote translation of cellular RNAs through internal ribosome entry sites [56] and also appears in nutritional stress granules in *T*. *brucei* [26]. PUF proteins and UBP2 usually function as important post-transcriptional regulators [57–59], both are found in *T*. *brucei* and *Leishmania* nutritional stress granules. A few other RNA-binding domain proteins were found exclusively in the LeishIF4E-3 granules, thereby distinguishing them from *T*. *brucei* granules [26]. The significant enrichment of metabolic enzymes in the LeishIF4E-3 granules is an intriguing observation. However, recent reports have highlighted a non-classical RNA-binding activity of such enzymes, despite the absence of conventional RNA-binding domains [47]. A non-canonical moonlighting RNA-binding activity was also recorded for several metabolic enzymes in *T*. *brucei* [48], of which enolase (Tb927.10.2890) was shown to be enriched in our analysis. Other granular polypeptides enriched in the *Leishmania* LeishIF4E-3 containing granules were chaperones, along with signaling proteins, and proteins that presently play unknown roles in the granules, such as nuclear proteins.

Unlike the nutritional stress granules in *T*. *brucei* [26], the LeishIF4E-3 granules described in this paper do not contain RNA degradation enzymes as described in [60]. We also could not find typical P-body markers, DHH1 or Scd6, in accordance with our previous report that LeishIF4E-3-containing granules did not co-localize with DHH1 containing granules [34]. The absence of RNA degradation enzymes and P-body markers strengthens our conclusion that the LeishIF4E-3 containing granules described in this paper are dedicated mainly for storage of inactive mRNAs and stalled ribosomes, unlike the starvation granules described elsewhere [26]. Previous studies based on the content of P-bodies suggest that they are involved not only in storage of translationally repressed mRNAs, but possibly in their decay as well [17]. However, these reports were challenged by studies claiming that purified P-bodies are not involved in mRNA decay [61]. Altogether, it appears that P bodies could have a dynamic nature that is highly sensitive to the conditions that induce their formation and content. We can envision how different severities of nutritional stress in trypanosomatids could lead to the appearance of different subsets of granules and to dynamic changes between them. It is also possible that the different methodologies used to purify or enrich the granular fraction led to the identification of two granule sub-populations. Although both studies use a similar starvation protocol (incubation in PBS), the purification processes vary. *T*. *brucei* granules were obtained by sequential lysis and centrifugations using the cytoskeletal network as a molecular sieve. However, the *Leishmania* granules were enriched over sucrose gradients followed by pull-down of SBP-tagged LeishIF4E-3 over streptavidin beads.

Finally, all eIF4E orthologs are known to be phospho-proteins, whereby phosphorylation affects their function [62, 63]. Trypanosomatid eIF4Es are also phospho-proteins, but their phosphorylation sites follow a variable profiles. For example, LeishIF4E-4 is phosphorylated at several positions in its N-terminal extended region [64]. TbIF4E-3 is also phosphorylated in several positions in its extended N-terminus [45, 46]. Here we show that LeishIF4E-3 from *L*. *amazonensis* is phosphorylated in its N-terminal extension as well, but unlike the multiple sites in TbIF4E-3, it is phosphorylated only at position S75. This phosphorylation increased during nutritional stress. *L*. *amazonensis* phosphorylation of LeishIF4E-3 varies from that in other *Leishmania* species, since different sites were identified in *L*. *infantum*, (S84 and S105), as compared to S75 in *L*. *amazonensis*. S75 is not conserved in *L*. *infantum*, *L*. *major* or *L*. *donovani* [3] (S4D Fig). S84 in *L*. infantum is constitutively phosphorylated and S105 is phosphorylated mainly in promastigotes, and not in axenic amastigotes [3]. These findings further highlight differences between *Leishmania* species, in addition to the variability between leishmanias and trypanosomes.

In addition to the observed phosphorylation of LeishIF4E-3, additional modifications were noted, such as carbamidomethylation and oxidation, possibly explaining the migration pattern of the mutant protein. The S75A substitution of the phosphorylation site of LeishIF4E-3 from *L*. *amazonensis* reduced its ability to concentrate in granules, the mutant protein also reduced its capacity to bind LeishIF4G-4, though not completely.

The N-terminal extension of LeishIF4E-4 was recently shown to be responsible for binding LeishPABP1 and a similar prediction was made for LeishIF4E-3 in that paper [50]. Our analysis highlights that LeishIF4E-3 and LeishPABP2 co-migrate to the same granules. In that paper, the interaction of LeishIF4E-4 with LeishIF4G-3 was shown to be mediated by the conserved core region of LeishIF4E-4 [50]. Similar to the assembly mode of this complex, we previously reported that residue W187 of the LeishIF4E-3 core is required for the interaction with LeishIF4G-4 [34].It now appears that in addition to the core region of LeishIF4E-3, its phosphorylated N-terminus also influences this interaction. It is conceivable that substitution of the S75 phosphorylation site at the N-terminal region could have an allosteric effect on binding to LeishIF4G-4, possibly through alteration in the net charge of LeishIF4E-3 [65, 66]. Additionally, the interaction between the mutated LeishIF4E-3 and LeishIF4G-4 was reduced, but not completely diminished.

The formation of LeishIF4E-3-containing granules is reversible, since recovery of the nutrient supply caused the LeishIF4E-3 containing granules to disappear. It is not yet clear whether components comprising the nutritional granules were recycled back into the cytoplasm, or whether the granules were fully removed from the cells, possibly by secretion. Removal of metabolites from trypanosomatid cells via exosomes has been reported [67–69]. RNA secretion was also reported in trypanosomatids, whereby a heat shock stress that led to an inhibition of splicing caused the excess Spliced Leader RNA to concentrate in cytoplasmic vesicles, which are secreted as exosomes in a mechanism that resembles that of microRNA secretion by mammalian cells [70]. Whether a mechanism of similar nature also occurs for the starvation-induced LeishIF4E-3 containing granules and their associated mRNA molecules remains to be seen.

## Supporting information

S1 Fig **(A) Inhibitory effects of purine deprivation on translation are not affected by the presence or absence of dialyzed FCS.** Wild type *L. amazonensis* cells were subjected to purine deprivation for 1–4 days, with or without dialyzed FCS. Cycloheximide (CHX) treatment is shown as a control for complete inhibition of translation. Cells were incubated with 1 μg/ml puromycin for 20 min. Whole cell extracts (similar protein loads) were separated by 12% SDS-PAGE and subjected to western analysis using antibodies against puromycin. The bottom lane of Ponceau staining shows the protein loads. (B) Densitometrical analysis was performed on the gel. Each lane of western blots monitoring translation from Fig 1 was fully quantified using the Multi Gauge software, version 2.0. The values were normalized to the protein load. Densitometric analysis of translation is based on translation inhibition measured after starvation for 4 h (I) or 1–4 days (II) in the presence or absence of FCS (III).
(PDF)

S2 Fig Cytoplasmic distribution of LeishIF4E-3 and LeishIF4G-4 in response to different starvation conditions of *L. amazonensis* cell lines. **(A)** Field view of cells expressing LeishIF4G4-GFP that were subjected to different nutrient starvation conditions for 4 h. **(B)** Wild type cells incubated in PBS for 4 h. **(C)** Field view of cells shown in B. **(D)** Recovery experiment: Cells expressing LeishIF4G4-GFP were subjected to different nutrient starvation conditions for 4 h and allowed to recover in complete and supplemented DMEM growth medium for 24 h. **(E)** Field view of cells shown in D. **(F)** Cells expressing LeishIF4G4-GFP were subjected to purine starvation for 4 days in presence or absence of dialyzed FCS and allowed to recover in DMEM promastigote growth medium for 24 h. **(G)** Field view of cells shown in F. Following the different treatments the cells were fixed, permeabilized and processed for confocal microscopy. LeishIF4E-3 was detected using specific rabbit anti-LeishIF4E-3 antibodies and secondary DyLight-labeled antibodies (550 nm; red). LeishIF4G-4 was visualized through its fusion with GFP (488 nm; green). Nuclear and kinetoplast DNA was stained using DAPI (blue). A bright field (BF) picture of the cells is on the right.
(PDF)

S3 Fig Hybridization of *HSP83* probes in starvation-induced LeishIF4E-3 containing granules. **(A)** A field view of starved *L*. *amazonensis* wild type cells hybridized with a probe derived from the open reading frame of *HSP83* (761–1241). **(B)** No hybridization was observed for starved wild type cells hybridized with an *HSP83* intergenic region probe derived from positions (891–1118 of the intergenic region)**. (C)** A field view of cells shown in B. Cells were subjected to nutritional starvation for 12h, fixed, permeabilized and processed for mRNA FISH analysis. The *HSP83* mRNA was visualized using fluorescence *in situ* hybridization by a DIG-labeled probe corresponding to *HSP83*. Hybridization was detected using a FITC-labeled antibody against DIG (488 nm, green). LeishIF4E-3 was stained using rabbit specific antibodies against LeishIF4E-3 and detected using a DyLight-labeled secondary antibody (550 nm; red). Nuclear and kinetoplast DNA was stained using DAPI (blue). A bright field (BF) picture of the cells is shown.
(PDF)

S4 Fig Post translational modifications of LeishIF4E-3 in response to different starvation treatments. **(A)** Densitometric analysis of changes in the migration profile of LeishIF4E-3 due to post-translational modifications, shown in Fig 4A. Each lane of western blots from Fig 4A were quantified using the Multi Gauge, version 2.0 software. Histograms describe the densitometric analysis of LeishIF4E-3 modified forms (i.e., phosphorylated, intermediate or non-phosphorylated) following 4 h (I) or 24 h (II and III) of purine and amino acid depletion, respectively. **(B)** Changes in the migration profile of LeishIF4E-3 on SDS-PAGE are observed already after 1 h of incubation in PBS, with or without dialyzed FCS. Wild type *L*. *amazonensis* promastigotes were subjected to different starvation conditions for 1 h with **(I)** or without dialyzed FCS **(II).** Total cellular extracts were resolved on reduced bis-acrylamide 12% SDS-PAGE and subjected to western analysis using specific antibodies against LeishIF4E-3, LeishIF4G-4 or LeishIF4A. LeishIF4A served as loading control. **(C)** FCS deprivation does not change the LeishIF4E-3 migration pattern following purine starvation during 4 days. **(I)** Wild type *L*. *amazonensis* promastigotes were grown in medium lacking purines without FCS or in medium lacking purines in presence of 10% dialyzed FCS for 4 days. Total cellular extracts were resolved on reduced bis-acrylamide 12% SDS-PAGE and subjected to western analysis using antibodies against LeishIF4E-3. The bottom lane showing Ponceau staining verifies equal protein loads. **(II)** Densitometric analysis of modified LeishIF4E-3 following 4 days of purine starvation with or without dialyzed FCS. Each band in three different experiments was quantified using the Multi Gauge, version 2.0 software. **(D**) A phosphorylation site is located in the N-terminal extension of LeishIF4E-3. The phosphorylation sites in LeishIF4E-3 (marked with a star, *) are boxed in red for *L*. *amazonensis* (Ser 75) and in green for *L*. *infantum* (Ser 84, 105). The multiple phosphorylation sites in *T*. *brucei* are boxed in purple. The multiple sequence alignment was performed using MAFFT, version 7. Sequence conservations were generated by Jalview and are highlighted in greyscale.
(PDF)

S5 Fig Effect of the S75A mutation on migration of the mutant LeishIF4E-3 and its ability to granulate and to interact with LeishIF4G-4. **(A)** Densitometric analysis of steady-state expression of the endogenous and SBP-tagged LeishIF4E-3 in transgenic lines expressing the tagged LeishIF4E-3 and the S75A LeishIF4E-3 mutant under normal conditions and in starved cells. Each lane of western blots from Fig 5A and 5B were quantified using the Multi Gauge, version 2.0 software. Dot plots describe the densitometric analysis of LeishIF4E-3 forms (i.e. native or SBP-tagged) under non-starved and starved conditions. Dotted bars represent native LeishIF4E3 and SBP-tagged LeishIF4E-3. **(B)** Densitometric analysis of LeishIF4G-4 co-purification along with LeishIF4E-3 over streptavidin beads. Dot plots describe the densitometric analysis of pulled down proteins through SBP-tagged LeishIF4E-3 and SBP-tagged mutant (S75A) under non-starved conditions. Dotted bars represent LeishIF4G-4, native LeishIF4E3 and SBP-tagged LeishIF4E-3. **(C)** Broad field of cells shown in Fig 5C, demonstrating reduced granule formation by the mutant S75A LeishIF4E3 in response to PBS starvation. Transgenic *L*. *amazonensis* promastigotes expressing either SBP-tagged LeishIF4E-3 or SBP-tagged mutant LeishIF4E3 (S75A) were subjected to starvation in PBS for 4 h. The cells were then fixed, permeabilized and processed for confocal microscopy. LeishIF4E-3 was detected using rabbit anti-LeishIF4E-3 antibodies followed by incubation with anti-rabbit DyLight-labeled secondary antibodies (550 nm; red). The mutant SBP-tagged S75A LeishIF4E-3 was visualized using mouse monoclonal antibodies against SBP followed by incubation with anti-mouse DyLight-labeled secondary antibodies (488 nm; green). Nuclear and kinetoplast DNA was stained using DAPI (blue). Bright field pictures are shown on the right.
(PDF)

S6 Fig Control experiments for LeishIF4E-3 granule purification following PBS starvation. **(A)** Densitometric analysis of LeishIF4E-3 pulled-down from heavy sucrose fractions of gradients addressing starved and non-starved parasites. Bands representing the supernatant and eluted gradient fractions separated by SDS-PAGE and blotted (shown in Fig 6B) were quantified using the Multi Gauge, version 2.0 software. Each band from three different experiments of LeishIF4E-3 pulled-down representing non-starved (left bottom panel) or starved cells (right bottom panel) were quantified and values are presented here. **(B)** Incubation with cycloheximide does not affect LeishIF4E-3 granule assembly in *L*. *amazonensis* cells expressing LeishIF4G4-GFP starved by incubation in PBS for 4 h, either in the presence or absence of cycloheximide (100 μg/ml). The cells were fixed, permeabilized and processed for confocal microscopy. LeishIF4E-3 was detected using specific antibodies and secondary DyLight antibodies (550nm; red). LeishIF4G-4 was visualized through its fusion with GFP (488 nm; green). Nuclear and kinetoplast DNA was stained using DAPI (blue). Bright field pictures are shown on the right. **(C)** Broad field of cells shown in (B). **(D)** Western analysis showing that LeishIF4E-3 migrates in heavy sucrose fractions in the absence of cycloheximide. Transgenic *L*. *amazonensis* promastigotes expressing SBP-tagged LeishIF4E-3 were incubated in nutrient free buffer (PBS) for 12 h along with non-starved cells as control. Cell extracts were fractionated over 10–40% sucrose gradients in the absence of cycloheximide. Samples from the fractionated proteins were precipitated by TCA, resolved over 12% SDS-PAGE, electro-blotted and subjected to western analysis using antibodies against LeishIF4E-3. **(E**) A control experiment shows the pull-down of luciferase-SBP from heavy fractions of sucrose gradients following PBS starvation. Transgenic *L*. *amazonensis* promastigotes expressing SBP-tagged luciferase were incubated in nutrient-free buffer (PBS) for 12 h. Cell extracts were fractionated over 10–40% sucrose gradients. Fractions 25–42 were pooled and subjected to pull-down analysis using streptavidin-Sepharose beads. The wash fractions and eluted proteins were precipitated by TCA, and resolved by 12% SDS-PAGE, along with samples from the supernatant (S) and flow through (FT) fractions. The gel blots were subjected to western analysis using specific antibodies against the SBP tag.
(PDF)

S7 Fig A field view showing that LeishPABP2-SBP and LeishRPS6 co-localize in starvation-induced LeishIF4E-3 containing granules following nutrient deprivation. Transgenic *L*. *amazonensis* promastigotes expressing either SBP-tagged LeishPABP2 **(I)** or SBP-tagged LeishIF4E-3 **(II)** were subjected to nutrient starvation (PBS) for 4 h. The cells were then fixed, permeabilized and processed for confocal microscopy. **(I)** LeishIF4E-3 was immuno-stained with specific rabbit antibodies and secondary DyLight-labeled anti-rabbit antibodies (550 nm; red). LeishPABP2 was detected using mouse monoclonal antibodies against SBP and secondary anti-mouse DyLight antibodies (488 nm; green). **(II)** RPS6 was detected using specific rabbit antibodies and secondary DyLight anti-rabbit antibodies (550 nm; red). SBP-tagged LeishIF4E-3 was visualized with mouse monoclonal anti-SBP antibodies, detected with DyLight-labeled anti-mouse secondary antibodies (488 nm; green). Nuclear and kinetoplast DNA was stained using DAPI (blue). Bright field pictures are shown on the right.
(PDF)

S8 Fig Growth and viability curves of cultures grown following depletion of specific nutrients. Cell growth [top panel: (I)] was monitored by cell count using a Neubauer hemocytometer. Cell viability [bottom panel: (II)] was monitored by cell count using the trypan blue exclusion assay. Non-starved cells are shown in blue, purine-starved cells are shown in red, amino acid-starved cells are shown in purple and glucose-starved cells are shown in black.
(PDF)

S1 Video Movie portraying the motility of *L. amazonensis* promastigotes grown under normal conditions. *L*. *amazonensis* mid-log parasites (4–6 x 10^6^ cells/ml) were transferred to DMEM supplemented with FCS (10%) and other supplements (4 mM L-glutamine, 0.1 mM adenine, 5 μg/ml hemin, 40 mM Hepes, pH 7.5, 100 U/ml penicillin and 100 μg/ml streptomycin) for 24 h at 25°C. The video was recorded after 24 h of parasite growth in culture. The movie shows the motility of non-starved control promastigotes grown under normal conditions. Cells were visualized through phase contrast microscopy at 40x magnification using an Olympus IX73 inverted microscope. Videos were captured with a QImaging Retiga 6000 monochrome CCD camera.
(AVI)

S2 Video Movie portraying the effect of total nutrient deprivation on motility of *L. amazonensis* promastigotes in culture. *L*. *amazonensis* mid-log parasites (4–6 x 10^6^ cells/ml) were transferred to nutrient-free buffer (PBS) for 24 h at 25°C. The movie shows motility of *L*. *amazonensis* promastigotes starved by incubation in PBS for 24 h. Cells were visualized through phase contrast microscopy at 40x magnification, as described in the legend to S1 Video.
(AVI)

S3 Video Movie portraying the effect of purine deprivation on motility of *L. amazonensis* promastigotes in culture. Mid-log phase (4–6 x 10^6^ cells/ml) parasites were transferred to DMEM lacking purines supplemented with 4 mM L-glutamine, 5 μg/ml hemin, 40 mM Hepes, pH 7.5, 100 U/ml penicillin and 100 μg/ml streptomycin, for 24 h at 25°C. The movie shows the high motility of *L*. *amazonensis* promastigotes starved of purines for 24 h. Cells were visualized through phase contrast microscopy at 40x magnification as described in the legend to S1 Video.
(AVI)

S4 Video Movie portraying the effect of amino acid deprivation on motility of *L. amazonensis* promastigotes in culture. Mid log parasites (4–6 x 10^6^ cells/ml) were transferred to DMEM lacking amino acids, supplemented with 4 mM L-glutamine, 0.1 mM adenine, 5 μg/ml hemin, 40 mM Hepes, pH 7.5, 100 U/ml penicillin and 100 μg/ml streptomycin for 24h at 25°C. The movie was recorded after 24 h of parasite starvation. The movie shows the high motility of *L*. *amazonensis* promastigotes starved of amino acids for 24 h. Cells were visualized through phase contrast microscopy at 40x magnification as described in the legend to S1 Video.
(AVI)

S5 Video Movie portraying the effect of glucose deprivation on motility of *L. amazonensis* promastigotes in culture. Mid-log parasites (4–6 x 10^6^ cells/ml) were transferred to DMEM lacking glucose, supplemented with 4 mM L-glutamine, 0.1 mM adenine, 5 μg/ml hemin, 40 mM Hepes, pH 7.5, 100 U/ml penicillin and 100 μg/ml streptomycin, for 24 h at 25°C. The movie shows the slow motility of *L*. *amazonensis* promastigotes starved of glucose during 24 h. Cells were visualized through phase contrast microscopy at 40x magnification as described in the legend to S1 Video.
(AVI)

S1 Table Identification of the LeishIF4E-3 phospho-peptides. Extracts from wild type *L*. *amazonensis* cells, starved in PBS or non-starved were separated over SDS-PAGE and subjected to LC-MS/MS, with or without enrichment for phospho peptides over titanium dioxide beads. The resulting peptides were identified using the Proteome Discoverer software version 1.4 with the Sequest algorithm that compared them with the *L*. *amazonensis* LeishIF4E-3 sequence (38). Post translational modifications are described in column E. The probability of the phosphorylation found at position S75 was calculated using the phosphoRS tool (column I).
(XLSX)

S2 Table Proteomic content of starvation-induced LeishIF4E-3 containing granules—manual classification. Starvation-induced LeishIF4E-3 containing granules, enriched over sucrose gradients, were subjected to pull-down analysis over streptavidin beads. The proteomic content was assessed by LC-MS/MS. Proteins were identified using the MaxQuant software. Enrichment analysis, as compared to the luciferase control, was determined using the Perseus statistical tool. Proteins with at least two fold increase and a significance of Padj<0.05 were highlighted. Identified proteins that were enriched in the starvation-induced LeishIF4E-3 containing granules were categorized into groups according to function.
(XLSX)

S3 Table Proteomic content of starvation-induced LeishIF4E-3 containing granules–classification by GO term enrichment. Starvation-induced LeishIF4E-3 containing granules, enriched over sucrose gradients, were subjected to pull-down analysis over streptavidin beads and compared to the proteins pulled down from SBP-tagged luciferase expressing cells. The proteomic content was assessed by LC-MS/MS as described in the Materials and Methods chapter. The granular enriched proteins given in S2 Table, were analyzed by the GO term enrichment tool based on cellular component. All GO terms were enriched by at least four fold as compared to the gene sets encoded in the genome, with a p< 0.01.
(XLSX)
