# Supplementary material for: Nutritional stress targets LeishIF4E-3 to storage granules that contain RNA and ribosome components in Leishmania
Source: PLoS Negl Trop Dis. 2019 Mar 14;13(3):e0007237. doi: 10.1371/journal.pntd.0007237 (PMC6435199; doi:10.1371/journal.pntd.0007237)
Supplement: S1 Fig — (A) Inhibitory effects of purine deprivation on translation are not affected by the presence or absence of dialyzed FCS. Wild type L. amazonensis cells were subjected to purine deprivation for 1–4 days, with or without dialyzed FCS. Cycloheximide (CHX) treatment is shown as a control for complete inhibition of translation. Cells were incubated with 1 μg/ml puromycin for 20 min. Whole cell extracts (similar protein loads) were separated by 12% SDS-PAGE and subjected to western analysis using antibodies against puromycin. The bottom lane of Ponceau staining shows the protein loads. (B) Densitometrical analysis was performed on the gel. Each lane of western blots monitoring translation from Fig 1 was fully quantified using the Multi Gauge software, version 2.0. The values were normalized to the protein load. Densitometric analysis of translation is based on translation inhibition measured after starvation for 4 h (I) or 1–4 days (II) in the presence or absence of FCS (III). (PDF) [file pntd.0007237.s001.pdf]

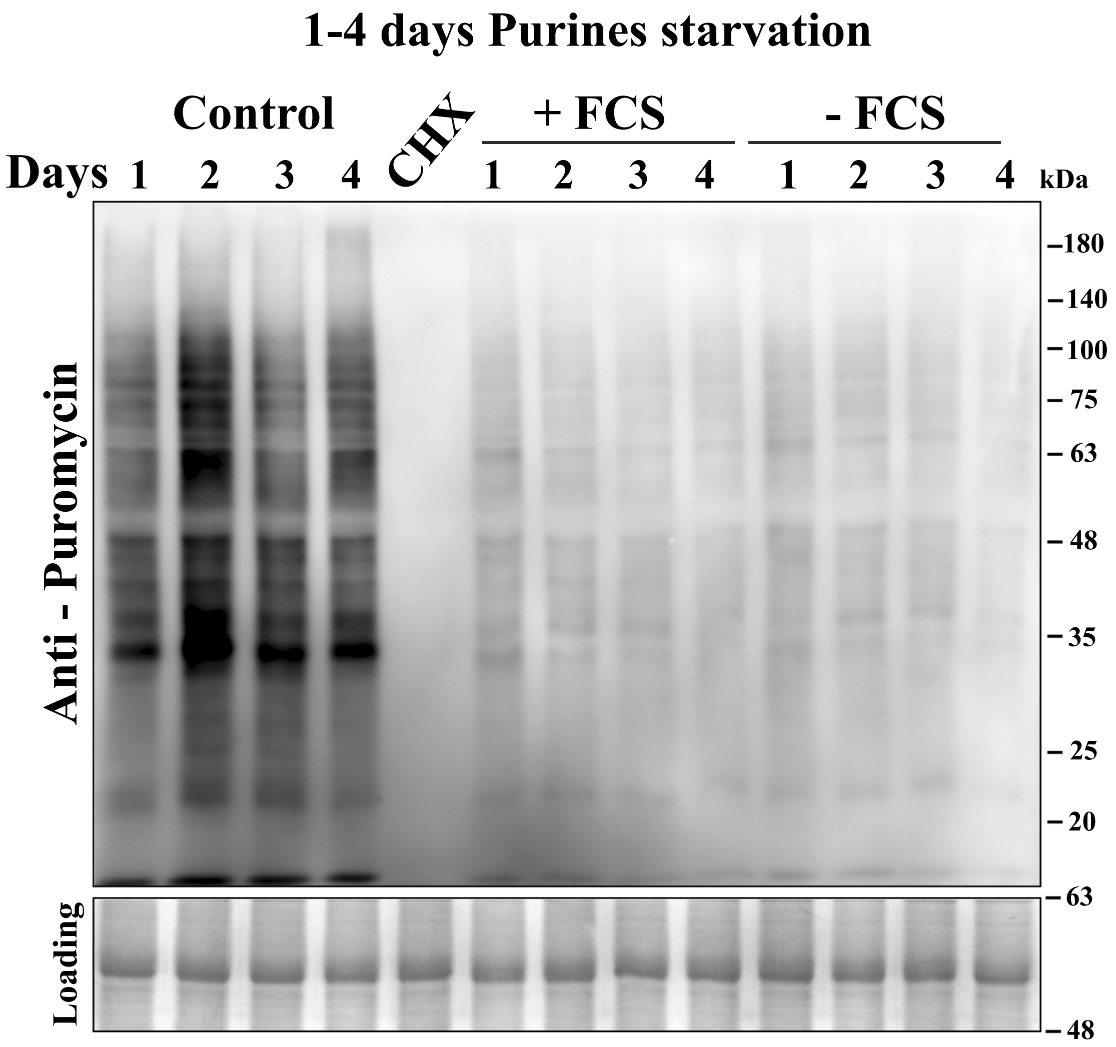

S1A Fig. Inhibitory effects of purine deprivation on translation are not affected by the presence or absence of dialyzed FCS. Wild type *L. amazonensis* cells were subjected to purine deprivation for 1-4 days, with or without dialyzed FCS. Cycloheximide (CHX) treatment is shown as a control for complete inhibition of translation. Cells were incubated with 1 µg/ml puromycin for 20 min. Whole cell extracts (similar protein loads) were separated by 12% SDS-PAGE and subjected to western analysis using antibodies against puromycin. The bottom lane of Ponceau staining shows the protein loads.

S1B Fig

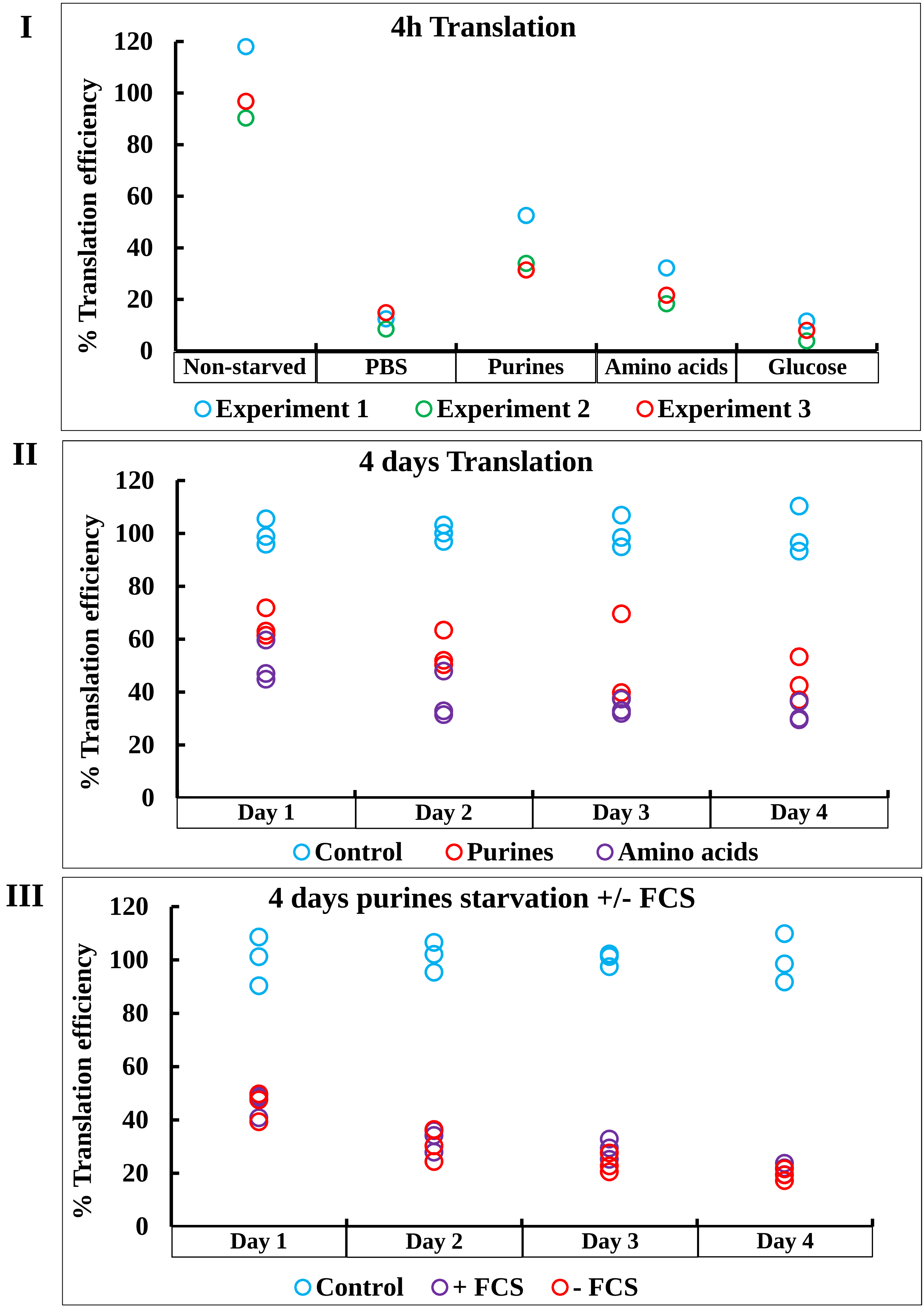
I
4h Translation
% Translation efficiency
0
20
40
60
80
100
120
Non-starved
PBS
Purines
Amino acids
Glucose
Experiment 1
Experiment 2
Experiment 3
II
4 days Translation
% Translation efficiency
0
20
40
60
80
100
120
Day 1
Day 2
Day 3
Day 4
Control
Purines
Amino acids
III
4 days purines starvation +/- FCS
% Translation efficiency
0
20
40
60
80
100
120
Day 1
Day 2
Day 3
Day 4
Control
+ FCS
- FCS

S1B Fig. Densitometric analysis of the effect of different starvation regimes on translation shown in Figure 1. Densitometrical analysis was performed using Multi Gauge software, version 2.0, on each lane of the western blots. The values were normalized to the protein loads. Translation was monitored after starvation periods of 4 h (I) or 1-4 days (II) in the presence or absence of FCS (III).
