# Supplementary material for: Nutritional stress targets LeishIF4E-3 to storage granules that contain RNA and ribosome components in Leishmania
Source: PLoS Negl Trop Dis. 2019 Mar 14;13(3):e0007237. doi: 10.1371/journal.pntd.0007237 (PMC6435199; doi:10.1371/journal.pntd.0007237)
Supplement: S2 Fig — (A) Field view of cells expressing LeishIF4G4-GFP that were subjected to different nutrient starvation conditions for 4 h. (B) Wild type cells incubated in PBS for 4 h. (C) Field view of cells shown in B. (D) Recovery experiment: Cells expressing LeishIF4G4-GFP were subjected to different nutrient starvation conditions for 4 h and allowed to recover in complete and supplemented DMEM growth medium for 24 h. (E) Field view of cells shown in D. (F) Cells expressing LeishIF4G4-GFP were subjected to purine starvation for 4 days in presence or absence of dialyzed FCS and allowed to recover in DMEM promastigote growth medium for 24 h. (G) Field view of cells shown in F. Following the different treatments the cells were fixed, permeabilized and processed for confocal microscopy. LeishIF4E-3 was detected using specific rabbit anti-LeishIF4E-3 antibodies and secondary DyLight-labeled antibodies (550 nm; red). LeishIF4G-4 was visualized through its fusion with GFP (488 nm; green). Nuclear and kinetoplast DNA was stained using DAPI (blue). A bright field (BF) picture of the cells is on the right. (PDF) [file pntd.0007237.s002.pdf]

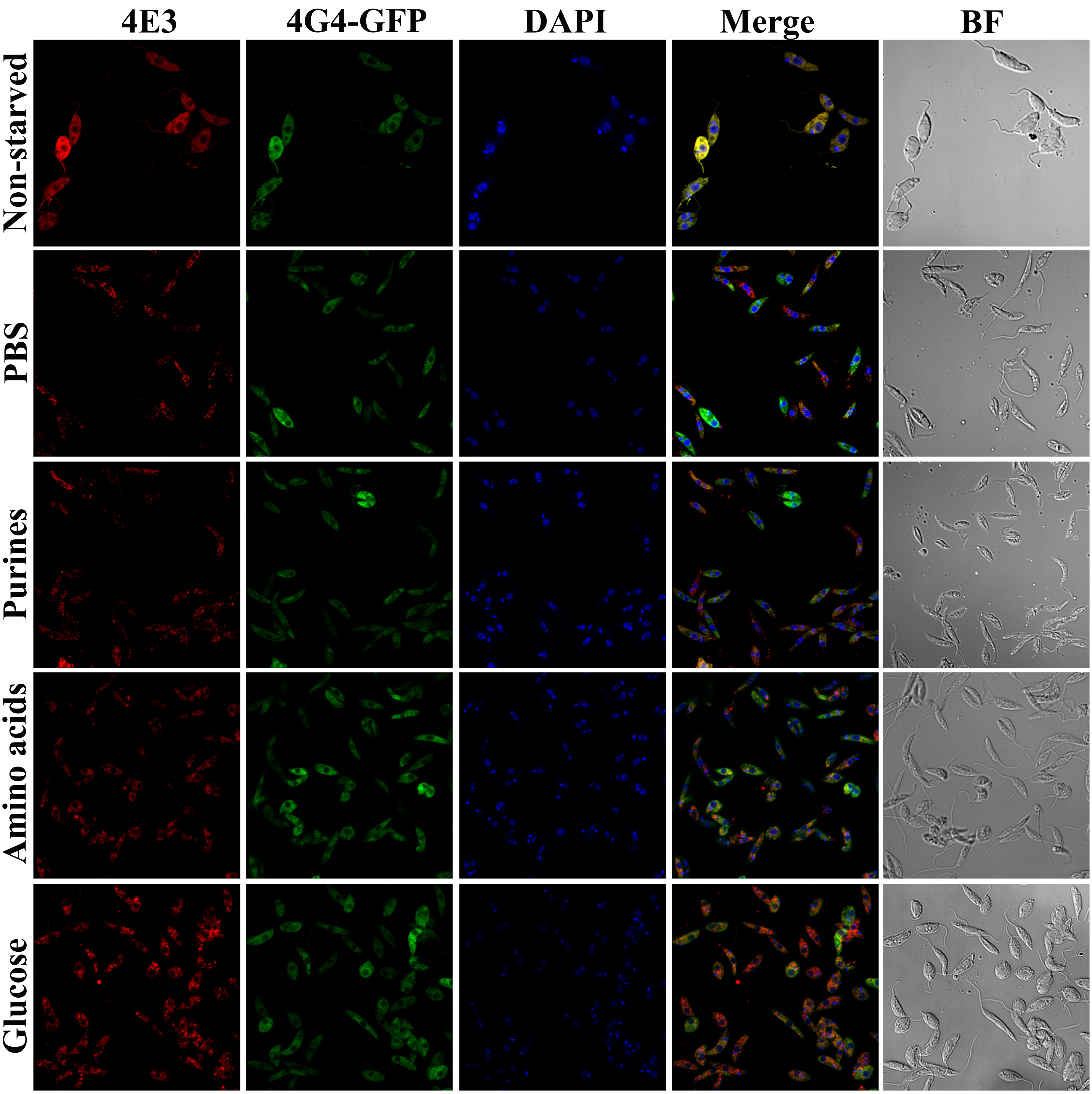

S2A Fig. A field view of the cytoplasmic distribution of LeishIF4E-3 and LeishIF4G-4 in response to different starvation conditions. *L. amazonensis* cells expressing LeishIF4G4-GFP were subjected to specific nutrient starvation for 4 h. The cells were then fixed, permeabilized and processed for confocal microscopy. LeishIF4E-3 was detected using specific rabbit anti-LeishIF4E-3 antibodies and secondary DyLight-labeled antibodies (550 nm; red). LeishIF4G-4 was visualized through its fusion with GFP (488 nm; green). Nuclear and kinetoplast DNA was stained using DAPI (blue). A bright field (BF) picture of the cells is on the right.

S2B Fig

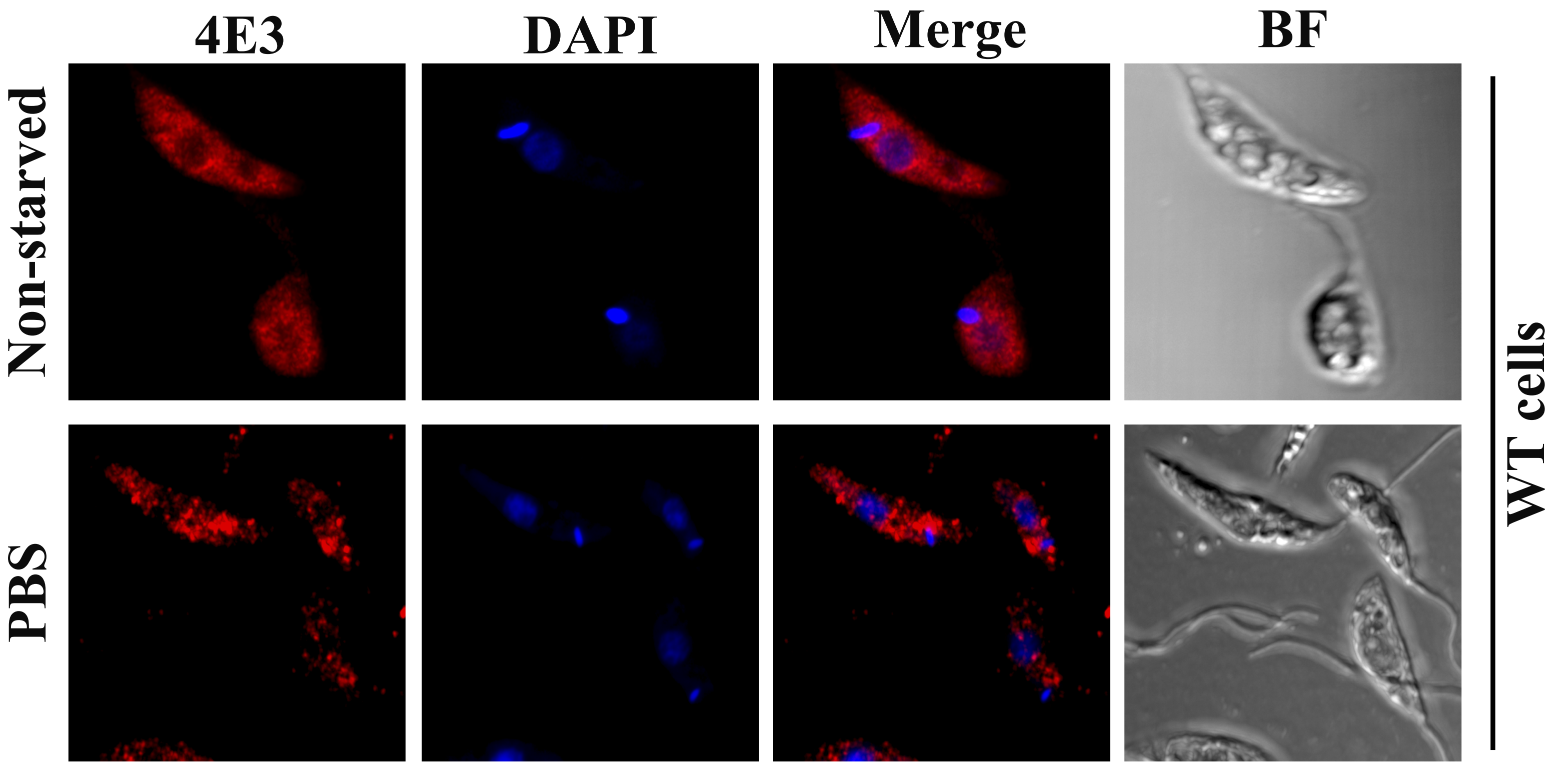

S2B Fig. Cytoplasmic distribution of LeishIF4E-3 in response to PBS starvation (4h) in wild type cells. Wild type *L. amazonensis* cells were subjected to nutritional starvation for 4 h. Cells were washed, fixed, permeabilized and processed for analysis in a confocal microscope. LeishIF4E-3 was stained using specific rabbit antibodies against LeishIF4E-3 and detected using secondary DyLight-labeled antibodies (550 nm; red). Nuclear and kinetoplast DNA were stained with DAPI (blue). A bright field (BF) picture of the cells is also shown.

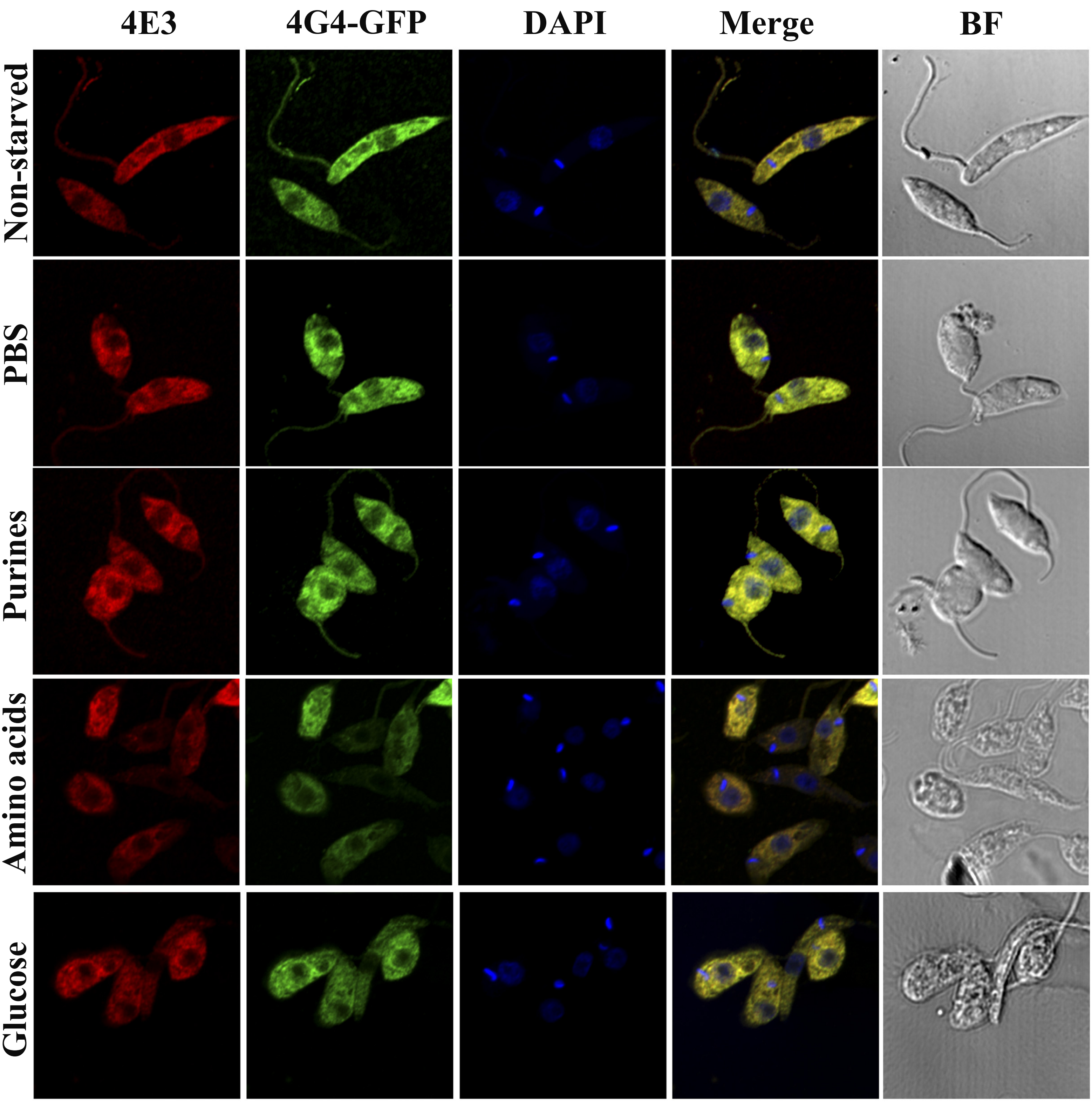

S2C Fig. Cytoplasmic distribution of LeishIF4E-3 and LeishIF4G-4 after a 24h recovery from different starvation conditions applied for 4 h. *L. amazonensis* cells expressing LeishIF4G4-GFP were subjected to specific nutrient starvation for 4 h and allowed to recover in complete DMEM and supplemented growth medium for 24 h. The cells were then fixed, permeabilized and processed for confocal microscopy. LeishIF4E-3 was stained using rabbit-raised anti-LeishIF4E-3 antibodies and detected using anti-rabbit DyLight-labeled secondary antibodies (550 nm). LeishIF4G-4 was visualized through its fusion with GFP. Nuclear and kinetoplast DNA was stained using DAPI (blue). A bright field (BF) picture of the cells is presented in the right column.

S2D Fig

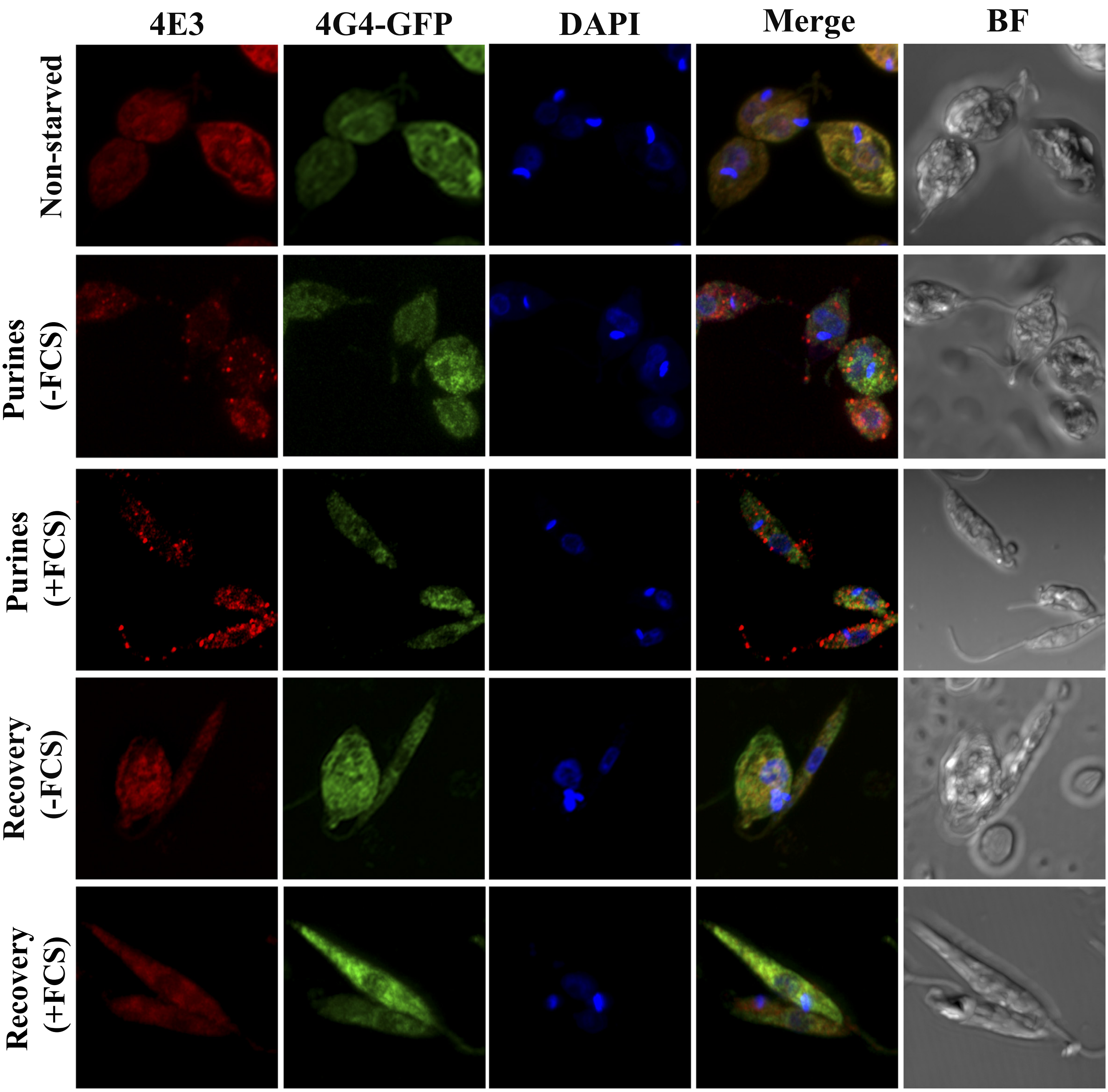

S2D Fig. Cytoplasmic distribution of LeishIF4E-3 and LeishIF4G-4 following 4 days of purine starvation with or without dialyzed FCS and following recovery for 24 h. *L. amazonensis* cells expressing LeishIF4G4-GFP were subjected to purine starvation for 4 days in presence or absence of dialyzed FCS and allowed to recover in DMEM promastigote growth medium for 24 h. The cells were then fixed, permeabilized and processed for confocal microscopy. LeishIF4E-3 was immuno-stained using antibodies against LeishIF4E-3 and detected using DyLight-labeled secondary antibodies (550 nm; red). LeishIF4G-4 was visualized through its fusion with GFP. Nuclear and kinetoplast DNA was stained using DAPI (blue). A bright field (BF) picture of the cells is presented in the right column.

S2E Fig

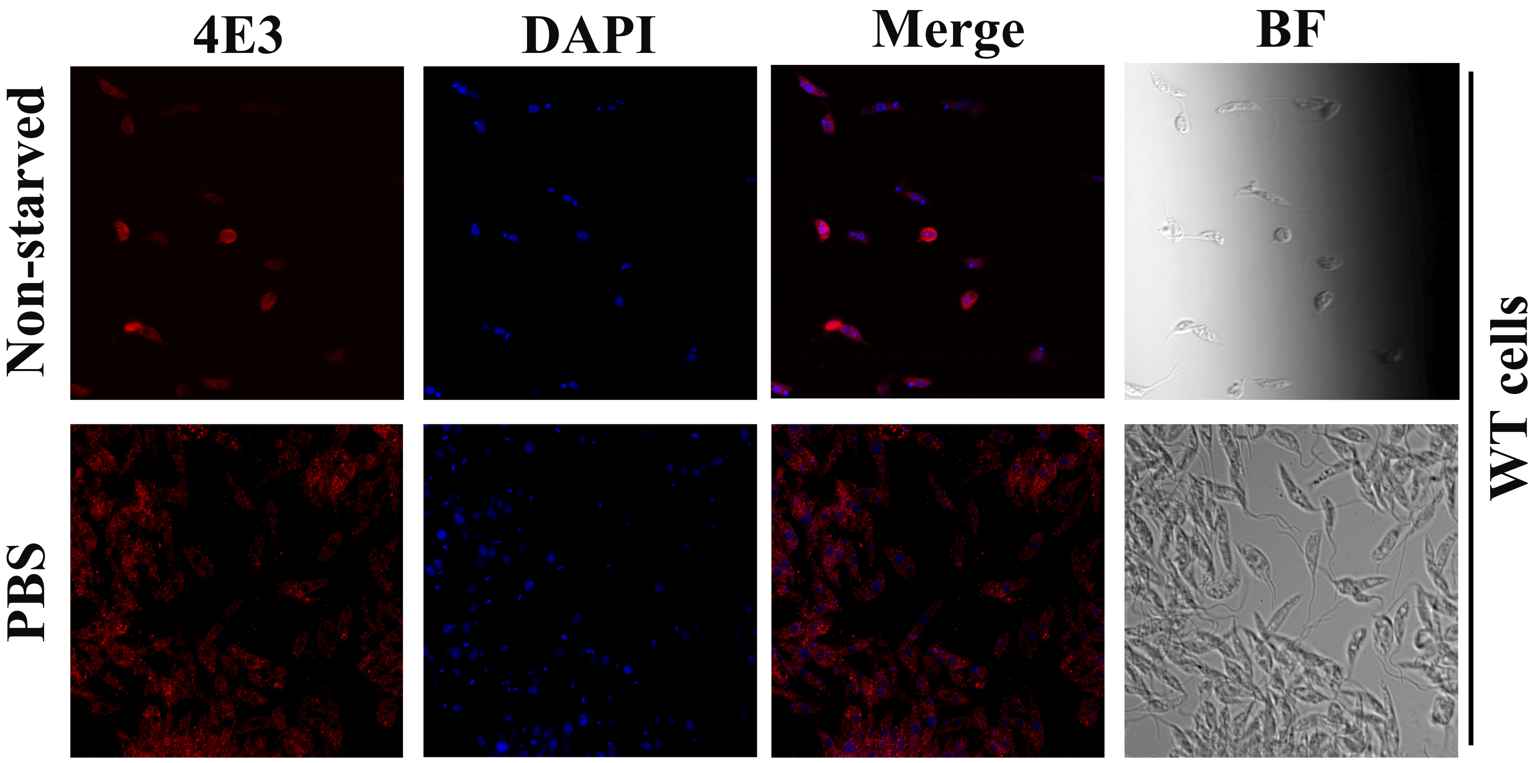

S2E Fig. A field view of the cytoplasmic distribution of LeishIF4E-3 in response to PBS starvation (4 h) in wild type cells. Wild type *L. amazonensis* cells were subjected to general nutrient starvation in PBS for 4 h. Cells were fixed, permeabilized and processed for analysis in a confocal microscope. LeishIF4E-3 was stained using specific antibodies against LeishIF4E-3 and DyLight-labeled secondary antibodies (550 nm; red). Nuclear and kinetoplast DNA was stained using DAPI (blue). A bright field (BF) picture of the cells is presented in the right column.

S2F Fig

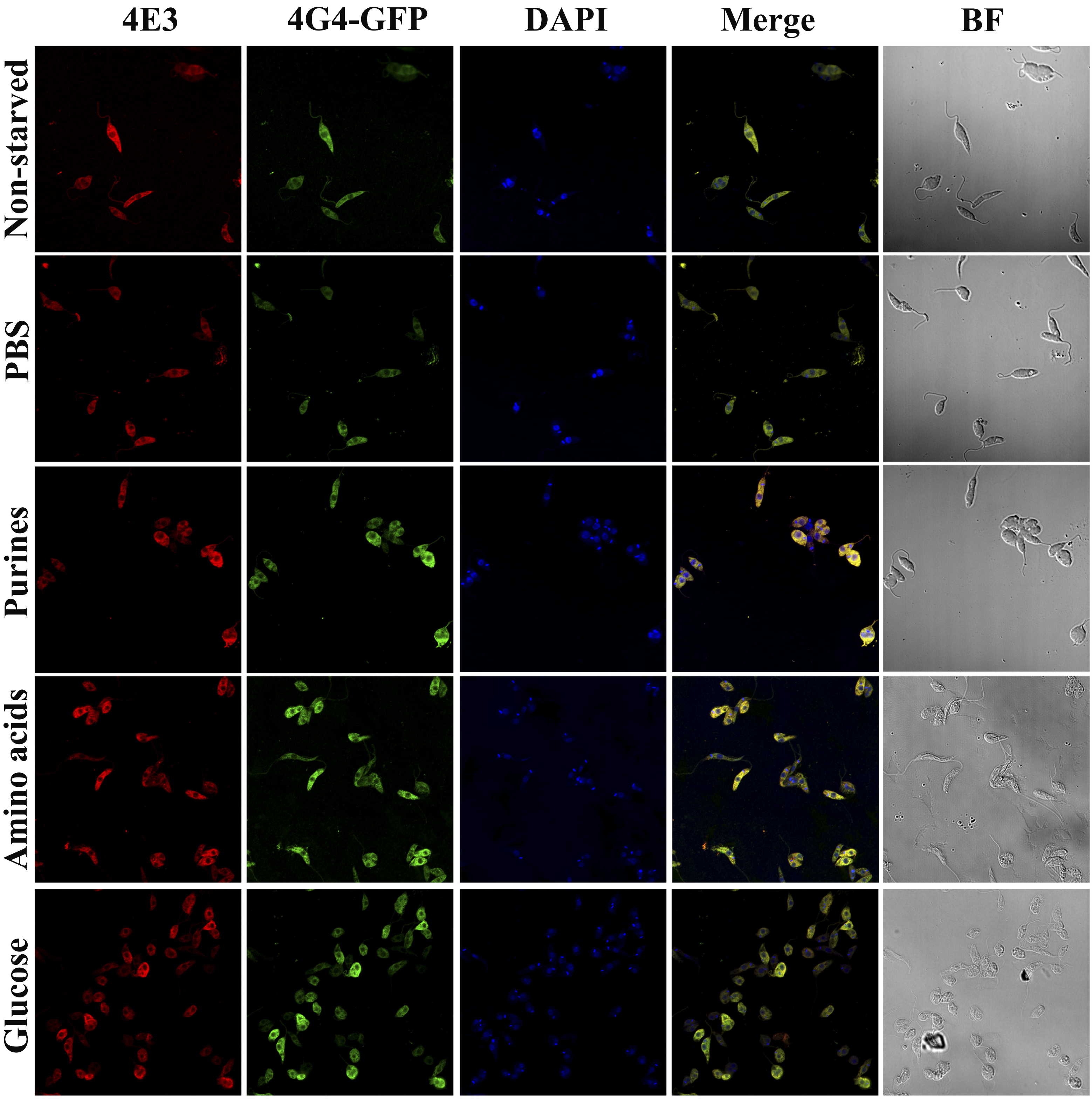

S2F Fig. A field view of the cytoplasmic distribution of LeishIF4E-3 and LeishIF4G-4 following recovery during 24 h from different starvation conditions applied for 4 h. *L. amazonensis* cells expressing LeishIF4G4-GFP were subjected to specific nutrient starvation for 4 h and allowed to recover in DMEM promastigote growth medium for 24 h. The cells were then fixed, permeabilized and processed for confocal microscopy. LeishIF4E-3 was stained using specific rabbit antibodies against LeishIF4E-3 and detected using DyLight-labeled secondary antibodies (550 nm; red). LeishIF4G-4 was visualized through its fusion with GFP. Nuclear and kinetoplast DNA was stained using DAPI (blue). A bright field (BF) picture of the cells is presented in the right column.

S2G Fig

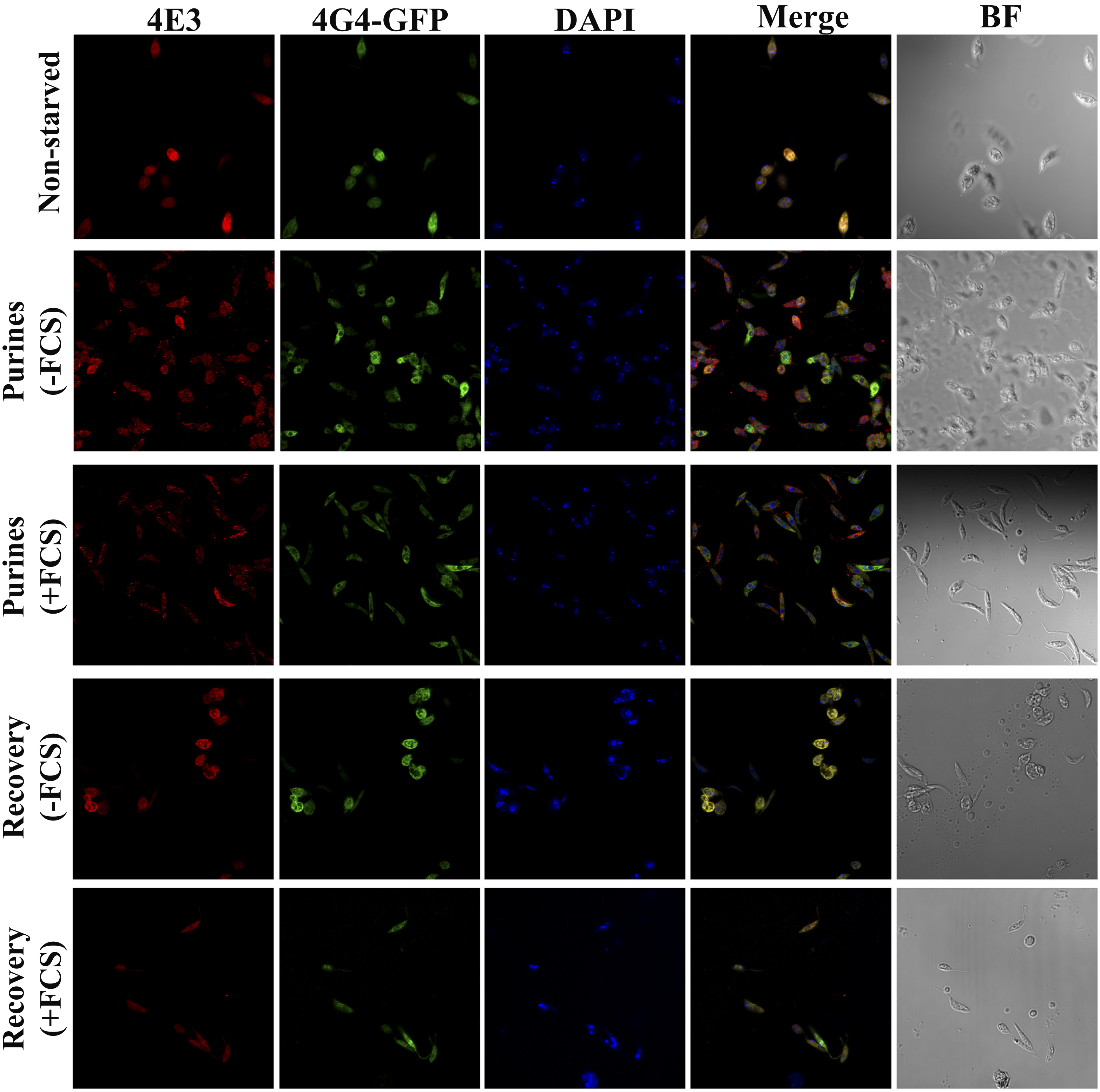

S2G Fig. A field view of the cytoplasmic distribution of LeishIF4E-3 and LeishIF4G-4 following 4 days of purine starvation with or without dialyzed FCS and following a 24 h recovery. *L. amazonensis* cells expressing LeishIF4G4-GFP were subjected to purine starvation for 4 days in presence or absence of dialyzed FCS and allowed to recover in complete DMEM and supplemented growth medium for 24 h. The cells were then fixed, permeabilized and processed for confocal microscopy. Fixed cells were incubated with anti-LeishIF4E-3 antibodies and detected using DyLight-labeled secondary antibodies (550 nm, red). LeishIF4G-4 was visualized through its fusion with GFP. Nuclear and kinetoplast DNA was stained using DAPI (blue). A bright field (BF) picture of the cells is presented in the right column.
