# Supplementary material for: Nutritional stress targets LeishIF4E-3 to storage granules that contain RNA and ribosome components in Leishmania
Source: PLoS Negl Trop Dis. 2019 Mar 14;13(3):e0007237. doi: 10.1371/journal.pntd.0007237 (PMC6435199; doi:10.1371/journal.pntd.0007237)
Supplement: S3 Fig — (A) A field view of starved L. amazonensis wild type cells hybridized with a probe derived from the open reading frame of HSP83 (761–1241). (B) No hybridization was observed for starved wild type cells hybridized with an HSP83 intergenic region probe derived from positions (891–1118 of the intergenic region). (C) A field view of cells shown in B. Cells were subjected to nutritional starvation for 12h, fixed, permeabilized and processed for mRNA FISH analysis. The HSP83 mRNA was visualized using fluorescence in situ hybridization by a DIG-labeled probe corresponding to HSP83. Hybridization was detected using a FITC-labeled antibody against DIG (488 nm, green). LeishIF4E-3 was stained using rabbit specific antibodies against LeishIF4E-3 and detected using a DyLight-labeled secondary antibody (550 nm; red). Nuclear and kinetoplast DNA was stained using DAPI (blue). A bright field (BF) picture of the cells is shown. (PDF) [file pntd.0007237.s003.pdf]

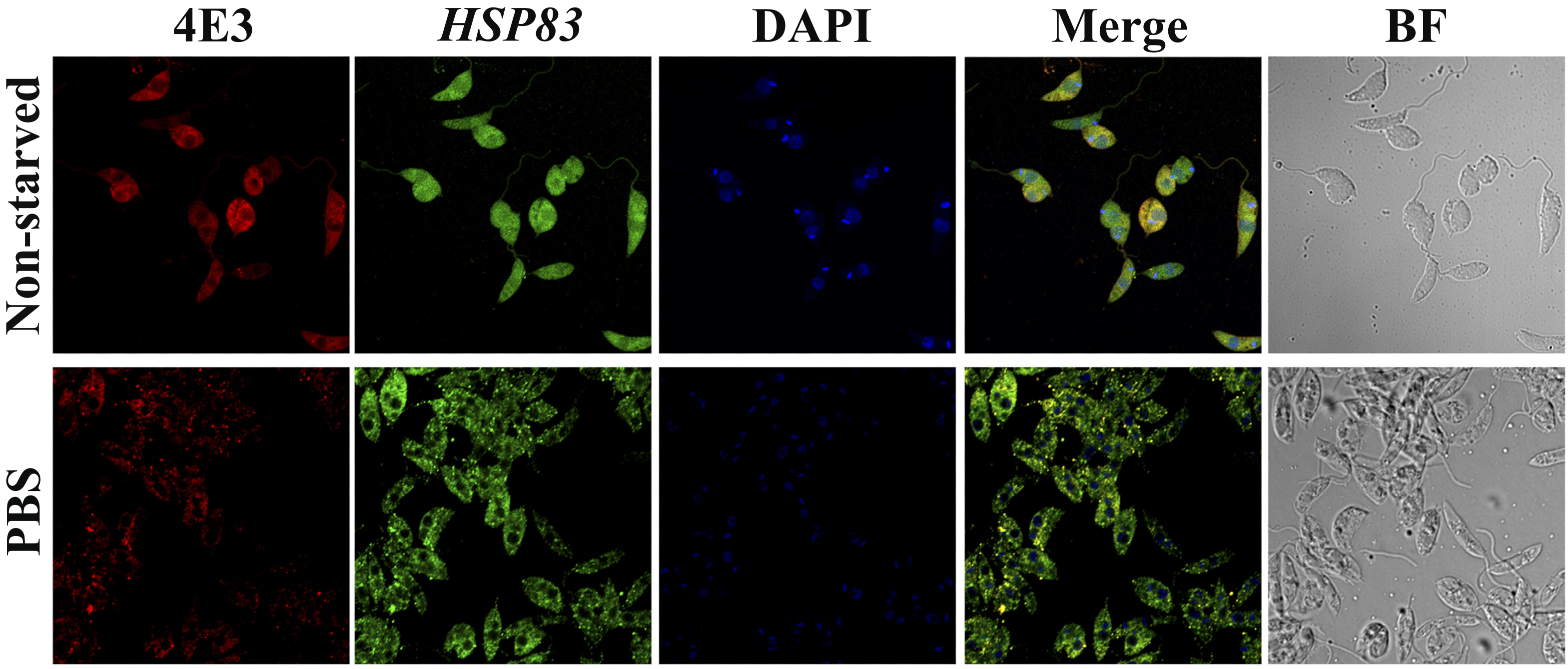

S3A Fig. A field view of *HSP83* mRNA hybridization in starvation-induced LeishIF4E-3 containing granules. Wild type *L. amazonensis* cells were subjected to nutritional starvation for 12h. Cells were then fixed, permeablized and processed for mRNA FISH analysis. The *HSP83* mRNA was visualized using fluorescence *in situ* hybridization by a DIG-labeled probe corresponding to *HSP83*. Hybridization was detected using a FITC-labeled antibody against DIG (488 nm, green). LeishIF4E-3 was stained using rabbit specific antibodies against LeishIF4E-3 and detected using a DyLight-labeled secondary antibody (550 nm; red). Nuclear and kinetoplast DNA was stained using DAPI (blue). A bright field (BF) picture of the cells is shown.

S3B Fig

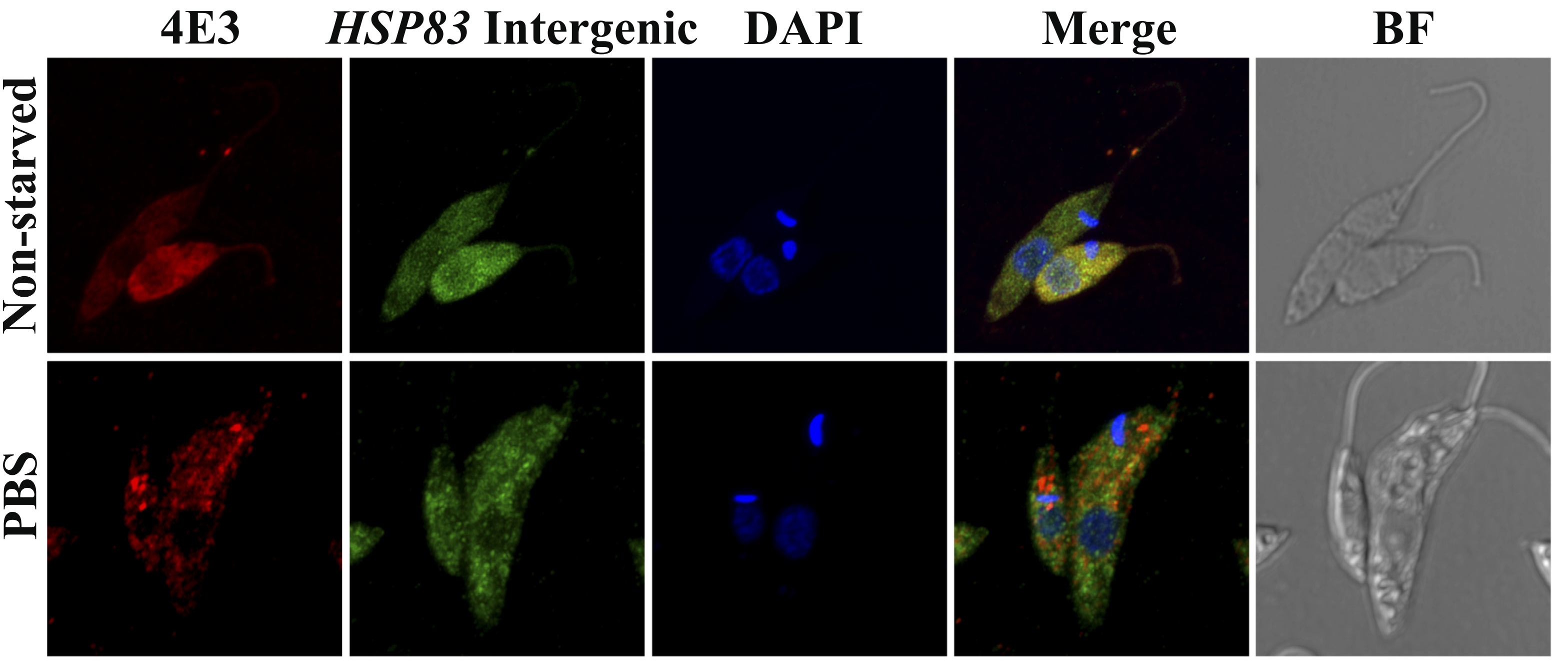

S3B Fig. Starvation-induced LeishIF4E-3 containing granules do not hybridize with a DIG-labelled probe directed at *HSP83* intergenic sequences. Wild type *L. amazonensis* cells were starved in PBS for 12 h. Cells were then fixed, permeabilized and processed for mRNA FISH analysis. mRNA were visualized using fluorescence *in situ* hybridization by DIG-labeled probe directed at *HSP83* intergenic sequences. Hybridization was detected using FITC-labeled antibodies against DIG (488 nm; green). Hybridized cells were incubated with rabbit-raised specific antibodies against LeishIF4E-3 and was detected using DyLight-labelled secondary antibodies (550 nm; red). Nuclear and kinetoplast DNA was stained using DAPI (blue). A bright field (BF) picture of the cells is also presented.

S3C Fig

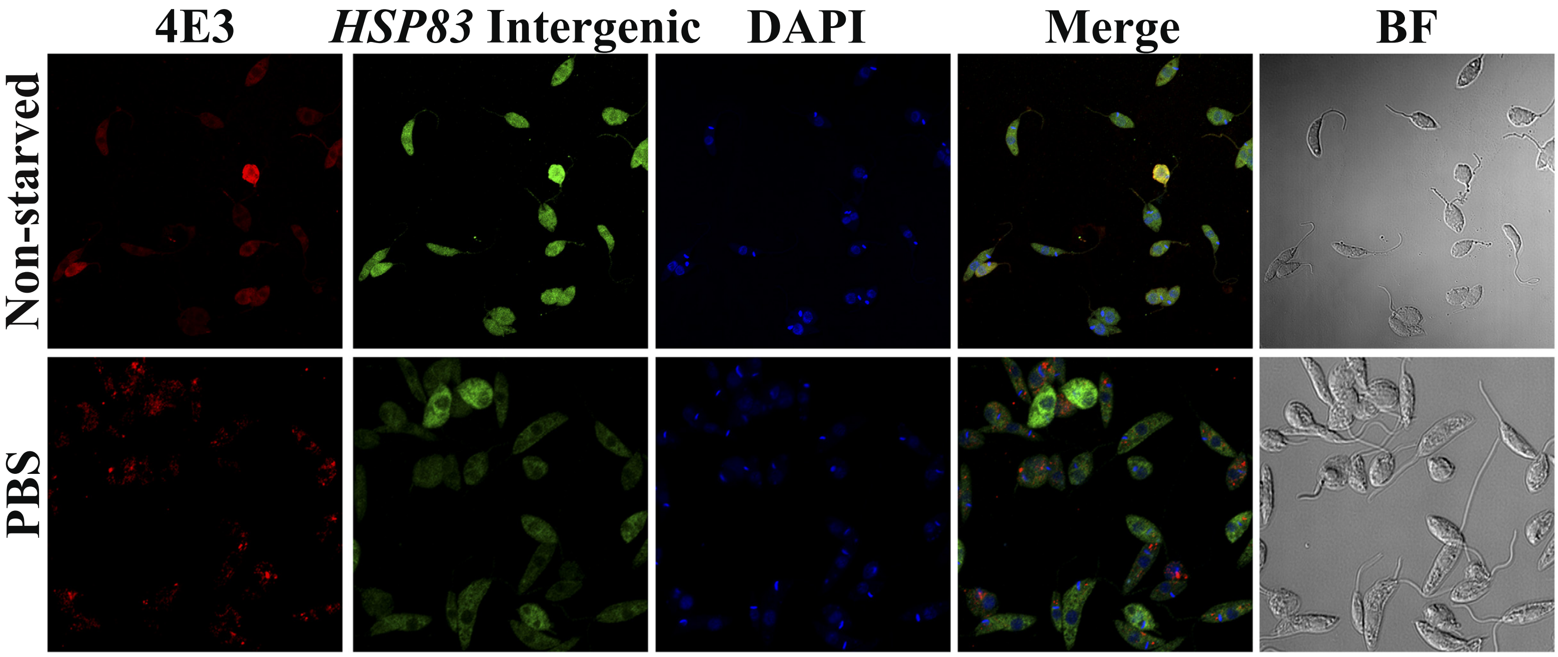

S3C. A field view showing that starvation-induced LeishIF4E-3 containing granules do not hybridize with the DIG-labelled probe directed at intergenic sequences of the *HSP83* locus. Wild type *L. amazonensis* cells were subjected to nutritional starvation for 12 h. Cells were then fixed, permeabilized and processed for mRNA FISH analysis. mRNAs were visualized using fluorescence *in situ* hybridization by DIG-labeled probe directed at the *HSP83* intergenic sequences. Hybridization was detected using FITC-labeled antibodies against DIG (488 nm; green). Hybridized cells were incubated with rabbit-raised specific antibodies against LeishIF4E-3 and detected using DyLight-labeled secondary antibodies (550 nm; red). Nuclear and kinetoplast DNA was stained using DAPI (blue). A bright field (BF) picture of the cells is also presented.
