# Supplementary material for: Nutritional stress targets LeishIF4E-3 to storage granules that contain RNA and ribosome components in Leishmania
Source: PLoS Negl Trop Dis. 2019 Mar 14;13(3):e0007237. doi: 10.1371/journal.pntd.0007237 (PMC6435199; doi:10.1371/journal.pntd.0007237)
Supplement: S4 Fig — (A) Densitometric analysis of changes in the migration profile of LeishIF4E-3 due to post-translational modifications, shown in Fig 4A. Each lane of western blots from Fig 4A were quantified using the Multi Gauge, version 2.0 software. Histograms describe the densitometric analysis of LeishIF4E-3 modified forms (i.e., phosphorylated, intermediate or non-phosphorylated) following 4 h (I) or 24 h (II and III) of purine and amino acid depletion, respectively. (B) Changes in the migration profile of LeishIF4E-3 on SDS-PAGE are observed already after 1 h of incubation in PBS, with or without dialyzed FCS. Wild type L. amazonensis promastigotes were subjected to different starvation conditions for 1 h with (I) or without dialyzed FCS (II). Total cellular extracts were resolved on reduced bis-acrylamide 12% SDS-PAGE and subjected to western analysis using specific antibodies against LeishIF4E-3, LeishIF4G-4 or LeishIF4A. LeishIF4A served as loading control. (C) FCS deprivation does not change the LeishIF4E-3 migration pattern following purine starvation during 4 days. (I) Wild type L. amazonensis promastigotes were grown in medium lacking purines without FCS or in medium lacking purines in presence of 10% dialyzed FCS for 4 days. Total cellular extracts were resolved on reduced bis-acrylamide 12% SDS-PAGE and subjected to western analysis using antibodies against LeishIF4E-3. The bottom lane showing Ponceau staining verifies equal protein loads. (II) Densitometric analysis of modified LeishIF4E-3 following 4 days of purine starvation with or without dialyzed FCS. Each band in three different experiments was quantified using the Multi Gauge, version 2.0 software. (D) A phosphorylation site is located in the N-terminal extension of LeishIF4E-3. The phosphorylation sites in LeishIF4E-3 (marked with a star, *) are boxed in red for L. amazonensis (Ser 75) and in green for L. infantum (Ser 84, 105). The multiple phosphorylation sites in T. brucei are boxed in purple. The multipl [file pntd.0007237.s004.pdf]

## (I) Starvation with dialyzed FCS

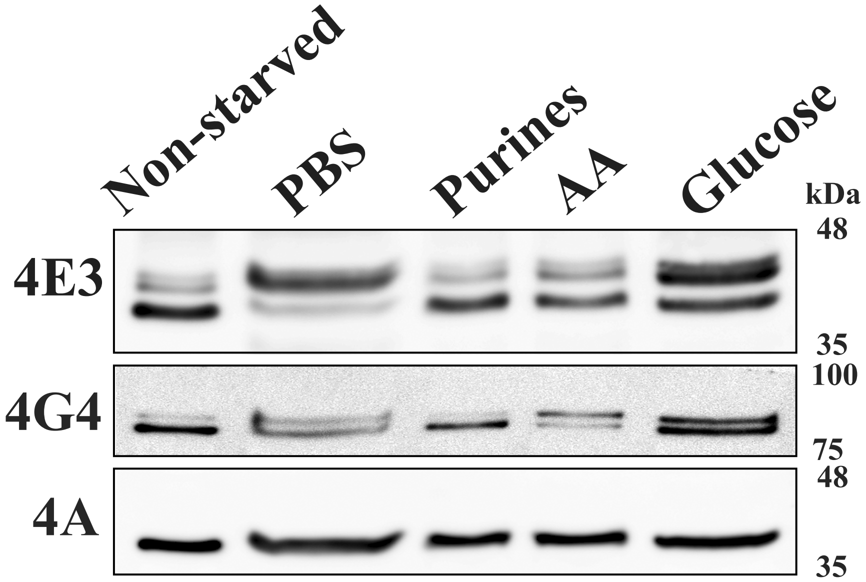


## (II) Starvation without FCS

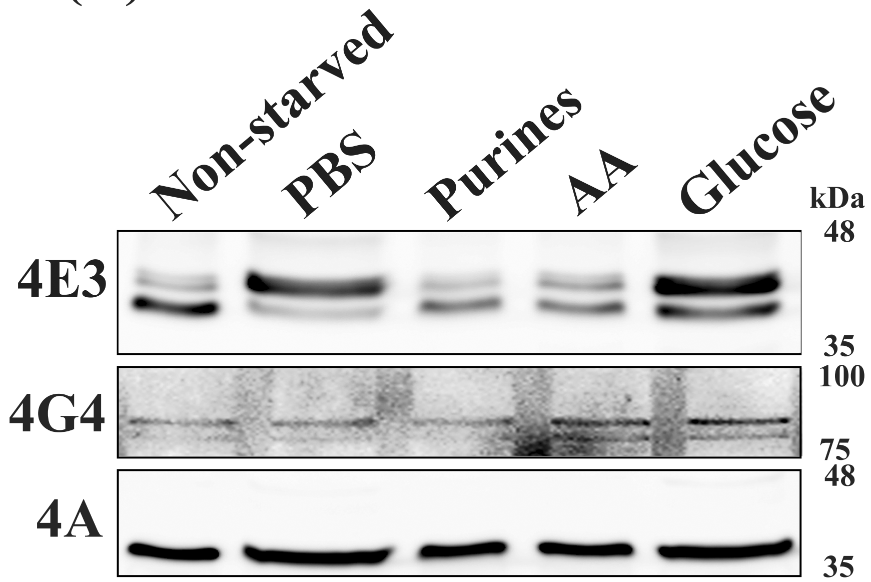


**S4A Fig. Changes in the migration profile of LeishIF4E-3 are observed already after 1 h of incubation in PBS, with or without dialyzed FCS.** Wild type *L. amazonensis* promastigotes were subjected to different starvation conditions for 1 h with (I) or without dialyzed FCS (II). Total cellular extracts were resolved on reduced bis-acrylamide 12% SDS-PAGE and subjected to western analysis using specific antibodies against LeishIF4E-3, LeishIF4G-4 or LeishIF4A. LeishIF4A served as loading control.

S4B Fig

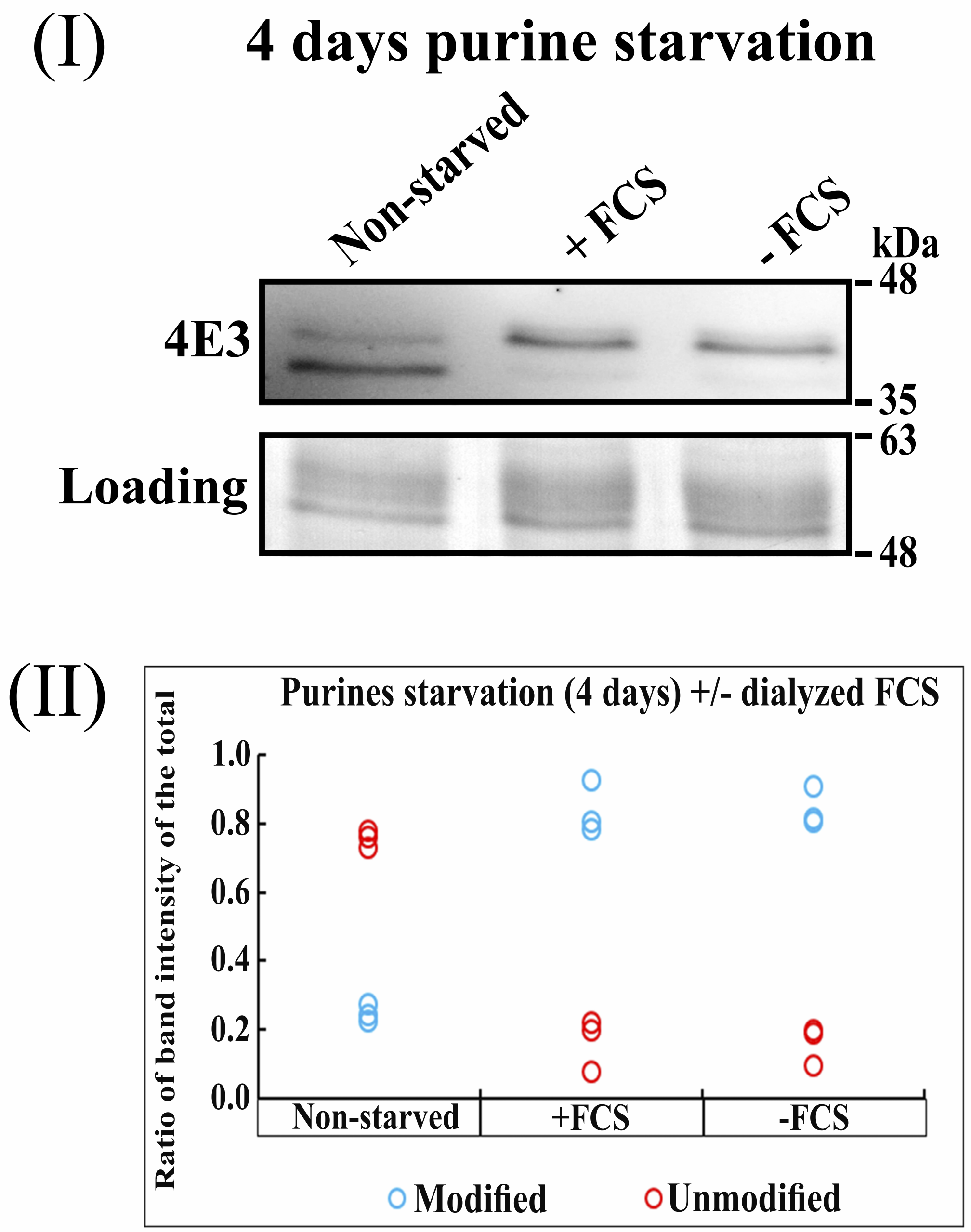


**S4B Fig. FCS deprivation does not change the LeishIF4E-3 migration pattern following purine starvation during 4 days.** **(I)** Wild type *L. amazonensis* promastigotes were grown in medium lacking purines without FCS or in medium lacking purines in presence of 10% dialyzed FCS for 4 days. Total cellular extracts were resolved on reduced bis-acrylamide 12% SDS-PAGE and subjected to western analysis using antibodies against LeishIF4E-3. The bottom lane showing Ponceau staining verifies equal protein loads. **(II)** Densitometric analysis of modified LeishIF4E-3 following 4 days of purine starvation with or without dialyzed FCS. Each band in three different experiments was quantified using the Multi Gauge, version 2.0 software.

# S4C Fig

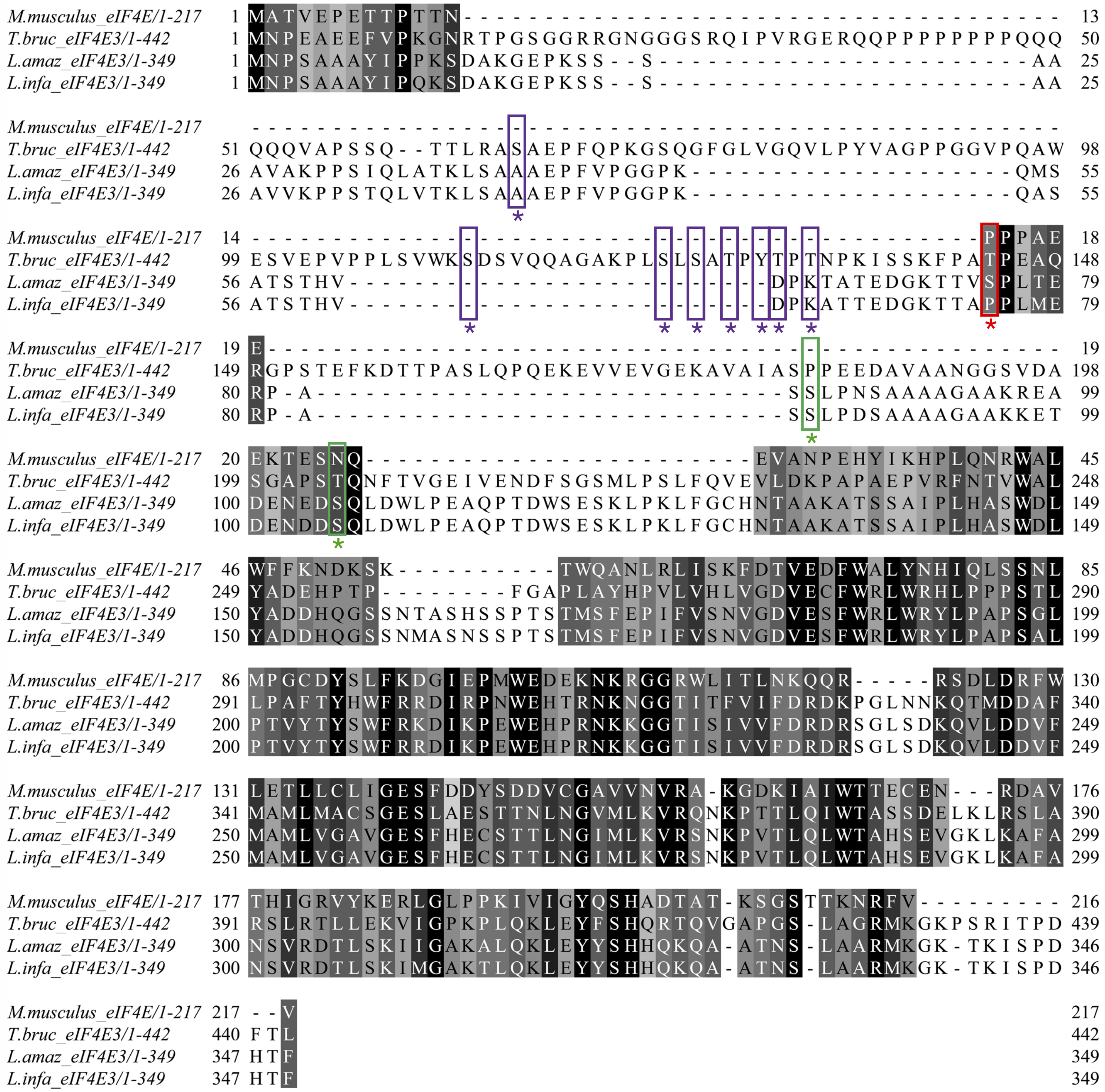


**S4C Fig. A phosphorylation site is located in the N-terminal extension of LeishIF4E-3 .** The phosphorylation sites in LeishIF4E-3 (marked with a star, *) are boxed in red for *L. amazonensis* (Ser 75) and in green for *L. infantum* (Ser 84, 105). The multiple phosphorylation sites in *T. brucei* are boxed in purple. The multiple sequence alignment was carried out using MAFFT, version 7. Sequence conservations were generated by Jalview and are highlighted in greyscale

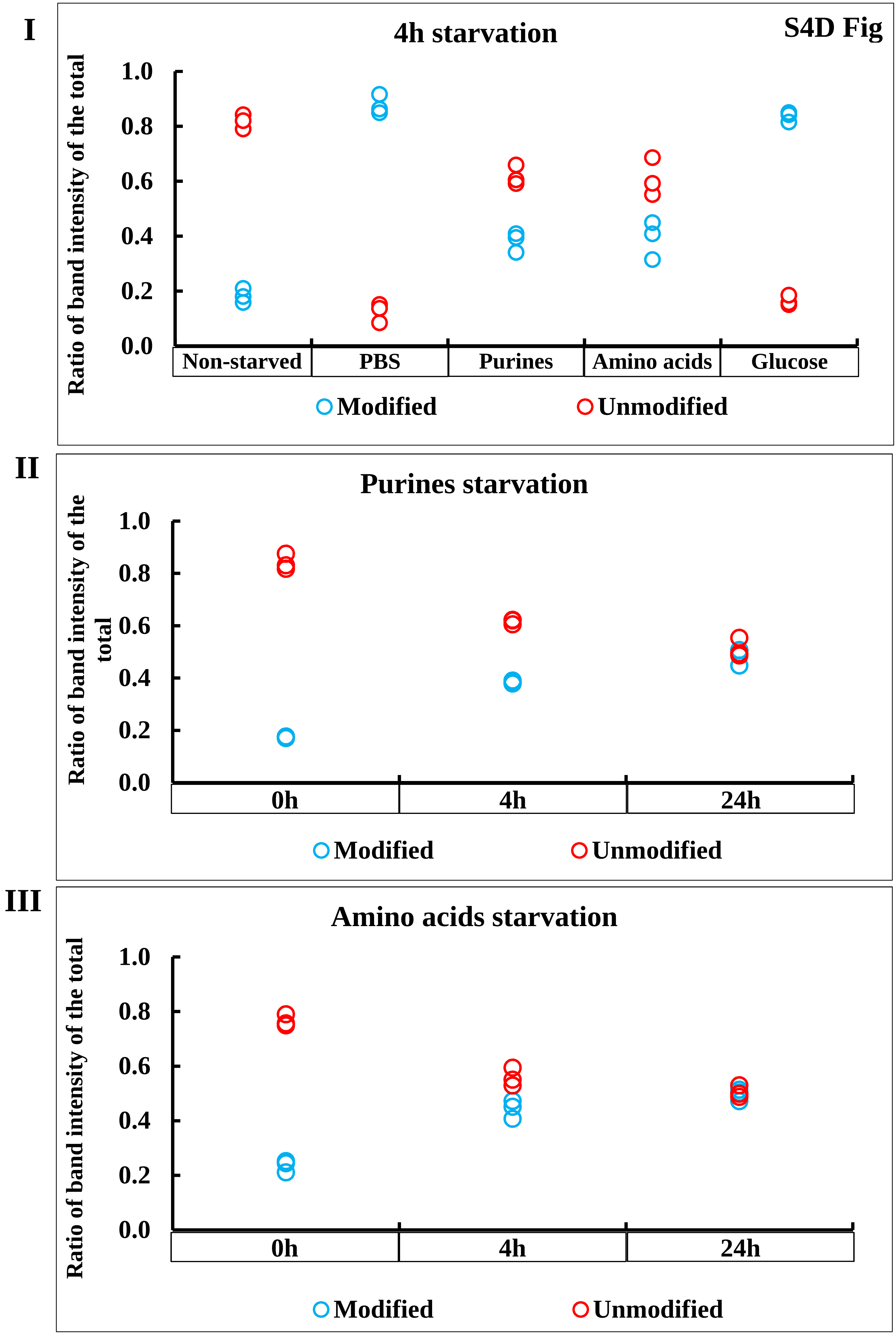
S4D Fig
I
4h starvation
Ratio of band intensity of the total
1.0
0.8
0.6
0.4
0.2
0.0
Non-starved
PBS
Purines
Amino acids
Glucose
Modified
Unmodified
II
Purines starvation
Ratio of band intensity of the total
1.0
0.8
0.6
0.4
0.2
0.0
0h
4h
24h
Modified
Unmodified
III
Amino acids starvation
Ratio of band intensity of the total
1.0
0.8
0.6
0.4
0.2
0.0
0h
4h
24h
Modified
Unmodified

**S4D Fig.** Densitometric analysis of modified LeishIF4E-3 forms following different starvation treatments. Each band of western blots from Figure 4A were quantified using the Multi Gauge, version 2.0 software. The measured values show the intensities of each LeishIF4E-3 modified form (i.e. modified or non-modified) following 4 h (I) or 24 h (II and III) of purine and amino acid depletion, respectively.
