# Supplementary material for: Nutritional stress targets LeishIF4E-3 to storage granules that contain RNA and ribosome components in Leishmania
Source: PLoS Negl Trop Dis. 2019 Mar 14;13(3):e0007237. doi: 10.1371/journal.pntd.0007237 (PMC6435199; doi:10.1371/journal.pntd.0007237)
Supplement: S5 Fig — (A) Densitometric analysis of steady-state expression of the endogenous and SBP-tagged LeishIF4E-3 in transgenic lines expressing the tagged LeishIF4E-3 and the S75A LeishIF4E-3 mutant under normal conditions and in starved cells. Each lane of western blots from Fig 5A and 5B were quantified using the Multi Gauge, version 2.0 software. Dot plots describe the densitometric analysis of LeishIF4E-3 forms (i.e. native or SBP-tagged) under non-starved and starved conditions. Dotted bars represent native LeishIF4E3 and SBP-tagged LeishIF4E-3. (B) Densitometric analysis of LeishIF4G-4 co-purification along with LeishIF4E-3 over streptavidin beads. Dot plots describe the densitometric analysis of pulled down proteins through SBP-tagged LeishIF4E-3 and SBP-tagged mutant (S75A) under non-starved conditions. Dotted bars represent LeishIF4G-4, native LeishIF4E3 and SBP-tagged LeishIF4E-3. (C) Broad field of cells shown in Fig 5C, demonstrating reduced granule formation by the mutant S75A LeishIF4E3 in response to PBS starvation. Transgenic L. amazonensis promastigotes expressing either SBP-tagged LeishIF4E-3 or SBP-tagged mutant LeishIF4E3 (S75A) were subjected to starvation in PBS for 4 h. The cells were then fixed, permeabilized and processed for confocal microscopy. LeishIF4E-3 was detected using rabbit anti-LeishIF4E-3 antibodies followed by incubation with anti-rabbit DyLight-labeled secondary antibodies (550 nm; red). The mutant SBP-tagged S75A LeishIF4E-3 was visualized using mouse monoclonal antibodies against SBP followed by incubation with anti-mouse DyLight-labeled secondary antibodies (488 nm; green). Nuclear and kinetoplast DNA was stained using DAPI (blue). Bright field pictures are shown on the right. (PDF) [file pntd.0007237.s005.pdf]

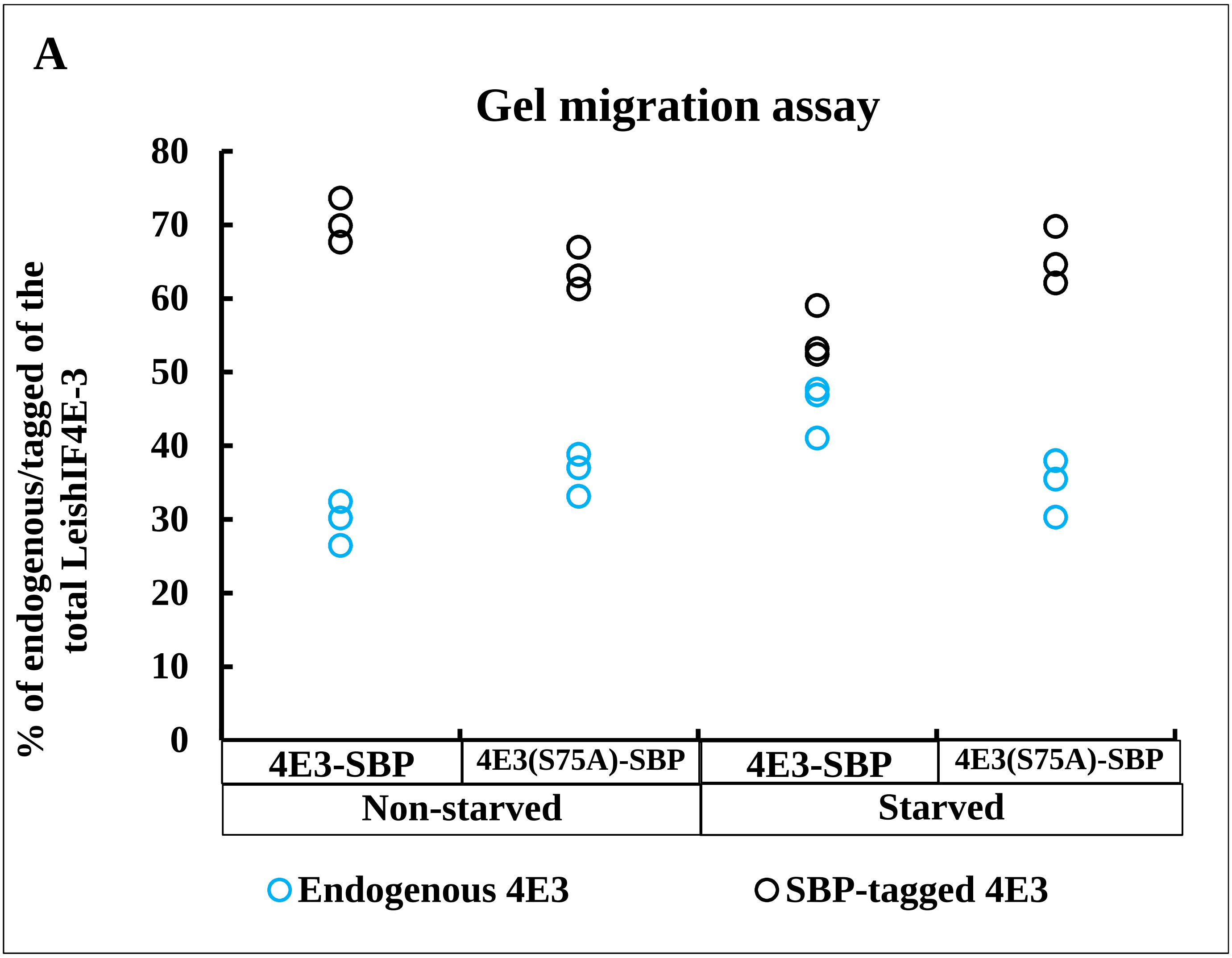
A
Gel migration assay
% of endogenous/tagged of the total LeishIF4E-3
0
10
20
30
40
50
60
70
80
4E3-SBP
4E3(S75A)-SBP
4E3-SBP
4E3(S75A)-SBP
Non-starved
Starved
Endogenous 4E3
SBP-tagged 4E3

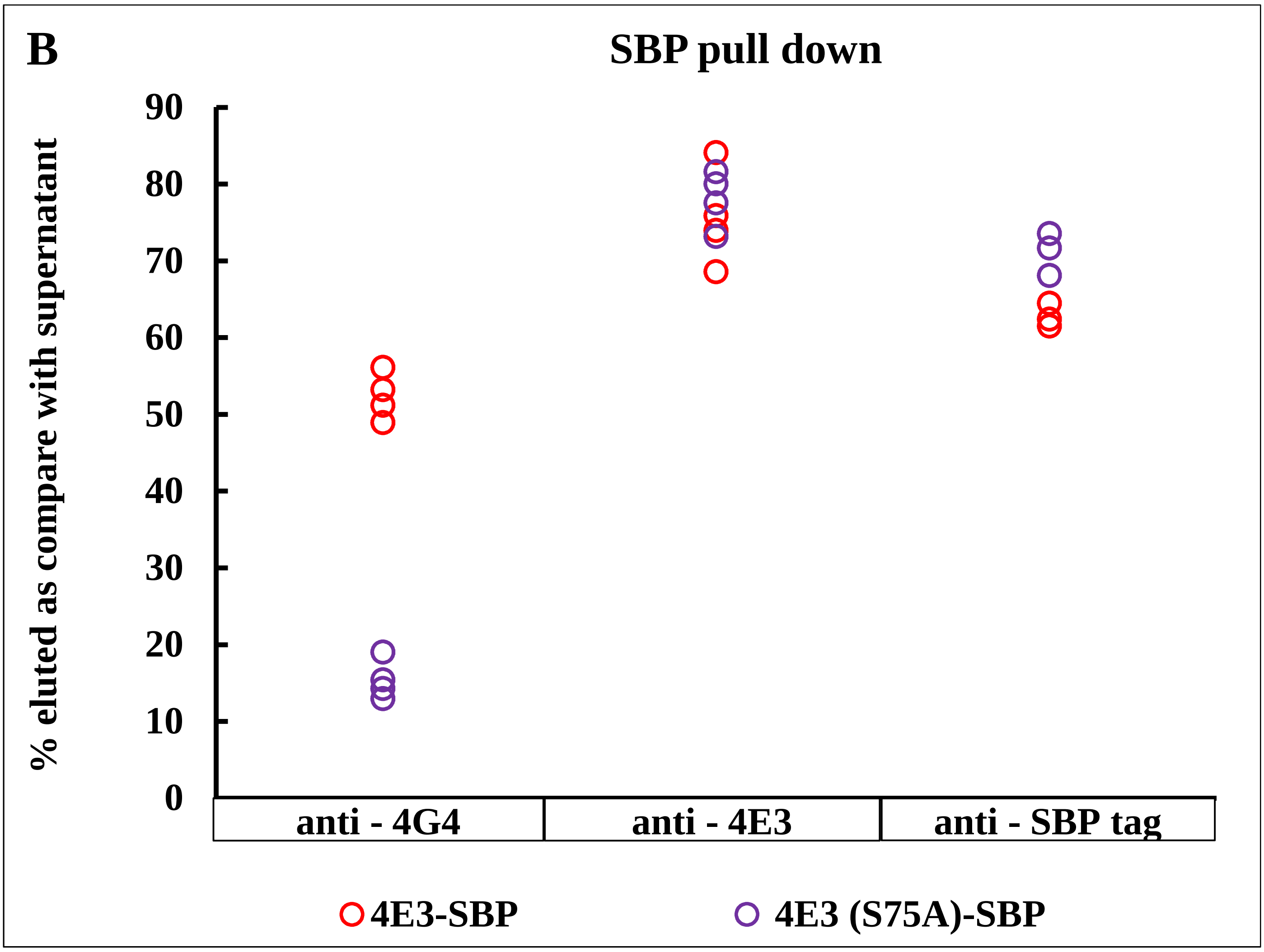
B
SBP pull down
% eluted as compare with supernatant
0
10
20
30
40
50
60
70
80
90
anti - 4G4
anti - 4E3
anti - SBP tag
4E3-SBP
4E3 (S75A)-SBP

S5 Fig. (A) Densitometric analysis of endogenous and SBP-tagged LeishIF4E-3 in transgenic lines expressing both proteins, under normal conditions and in starved cells. Each lane of western blots from Figure 5A and B were quantified using the Multi Gauge, version 2.0 software. Dot plots describe the densitometric analysis of LeishIF4E-3 forms (i.e. endogenous or SBP-tagged) under non-starved and starved conditions. **(B)** Densitometric analysis of LeishIF4G-4 co-purification along with LeishIF4E-3 over streptavidin beads. Dot plots describe the densitometric analysis of pulled down proteins through SBP-tagged LeishIF4E-3 and SBP-tagged mutant (S75A). Dotted bars represent LeishIF4G-4, native LeishIF4E3 and SBP-tagged LeishIF4E-3.

S5C Fig

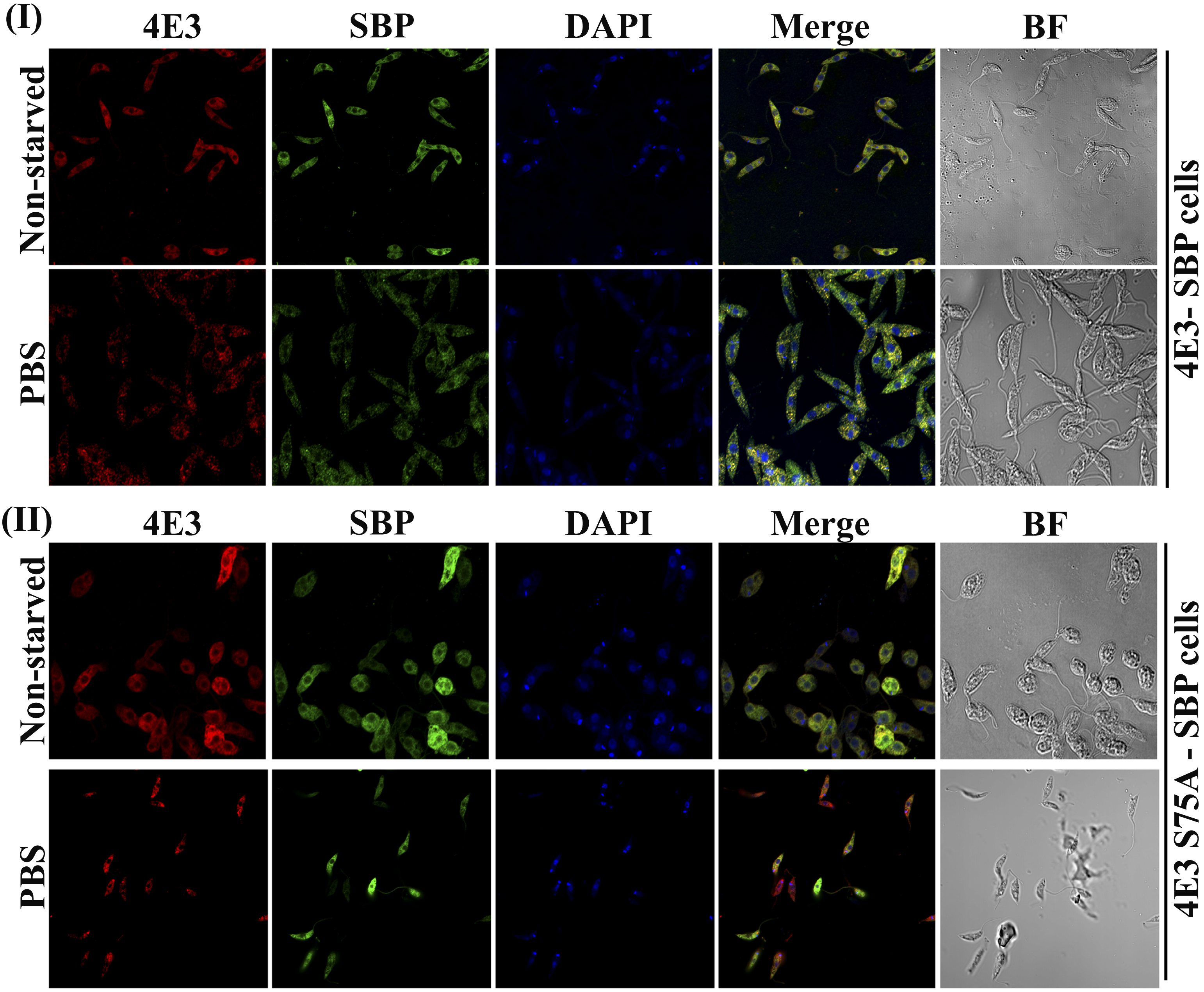

S5C Fig. Broad field showing reduced granule formation by the mutant S75A LeishIF4E3 in response to PBS starvation. Transgenic *L. amazonensis* promastigotes expressing either SBP-tagged LeishIF4E-3 or SBP-tagged mutant LeishIF4E3 (S75A) were subjected to starvation in PBS for 4 h. The cells were then fixed, permeabilized and processed for confocal microscopy. LeishIF4E-3 was detected using rabbit anti-LeishIF4E-3 antibodies followed by incubation with anti-rabbit DyLight-labeled secondary antibodies (550 nm; red). The mutant SBP-tagged S75A LeishIF4E-3 was visualized using mouse monoclonal antibodies against SBP followed by incubation with anti-mouse DyLight-labeled secondary antibodies (488 nm; green). Nuclear and kinetoplast DNA was stained using DAPI (blue). Bright field pictures are shown on the right.
