# Supplementary material for: Nutritional stress targets LeishIF4E-3 to storage granules that contain RNA and ribosome components in Leishmania
Source: PLoS Negl Trop Dis. 2019 Mar 14;13(3):e0007237. doi: 10.1371/journal.pntd.0007237 (PMC6435199; doi:10.1371/journal.pntd.0007237)
Supplement: S6 Fig — (A) Densitometric analysis of LeishIF4E-3 pulled-down from heavy sucrose fractions of gradients addressing starved and non-starved parasites. Bands representing the supernatant and eluted gradient fractions separated by SDS-PAGE and blotted (shown in Fig 6B) were quantified using the Multi Gauge, version 2.0 software. Each band from three different experiments of LeishIF4E-3 pulled-down representing non-starved (left bottom panel) or starved cells (right bottom panel) were quantified and values are presented here. (B) Incubation with cycloheximide does not affect LeishIF4E-3 granule assembly in L. amazonensis cells expressing LeishIF4G4-GFP starved by incubation in PBS for 4 h, either in the presence or absence of cycloheximide (100 μg/ml). The cells were fixed, permeabilized and processed for confocal microscopy. LeishIF4E-3 was detected using specific antibodies and secondary DyLight antibodies (550nm; red). LeishIF4G-4 was visualized through its fusion with GFP (488 nm; green). Nuclear and kinetoplast DNA was stained using DAPI (blue). Bright field pictures are shown on the right. (C) Broad field of cells shown in (B). (D) Western analysis showing that LeishIF4E-3 migrates in heavy sucrose fractions in the absence of cycloheximide. Transgenic L. amazonensis promastigotes expressing SBP-tagged LeishIF4E-3 were incubated in nutrient free buffer (PBS) for 12 h along with non-starved cells as control. Cell extracts were fractionated over 10–40% sucrose gradients in the absence of cycloheximide. Samples from the fractionated proteins were precipitated by TCA, resolved over 12% SDS-PAGE, electro-blotted and subjected to western analysis using antibodies against LeishIF4E-3. (E) A control experiment shows the pull-down of luciferase-SBP from heavy fractions of sucrose gradients following PBS starvation. Transgenic L. amazonensis promastigotes expressing SBP-tagged luciferase were incubated in nutrient-free buffer (PBS) for 12 h. Cell extracts were fractionated over 10–4 [file pntd.0007237.s006.pdf]

Supporting Information **S6A Fig**

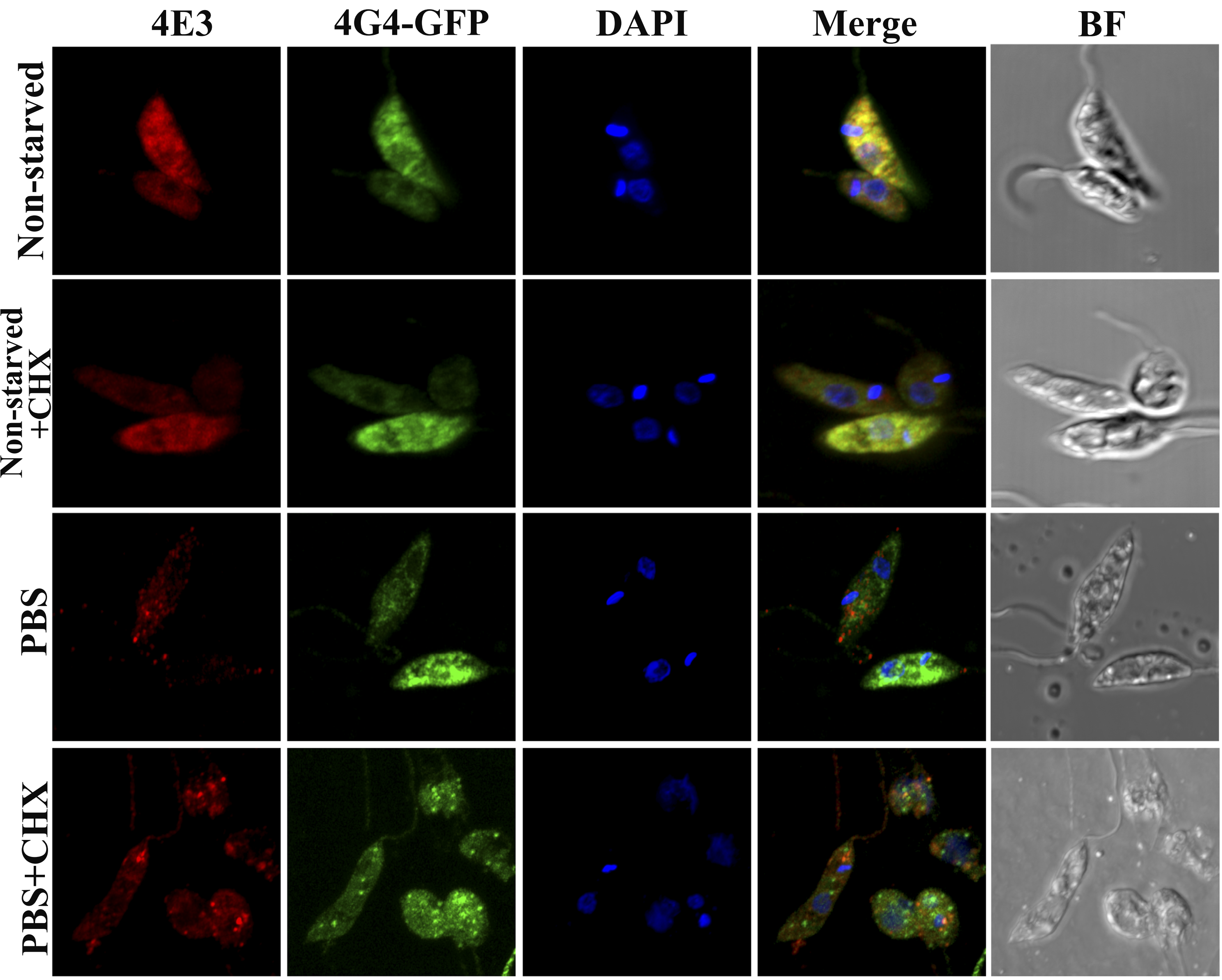

S6A Fig. Cycloheximide does not affect LeishIF4E-3 granule assembly following PBS starvation. *L. amazonensis* cells expressing LeishIF4G4-GFP were starved by incubation in PBS for 4 h, either in the presence or absence of cycloheximide (100 μg/ml). The cells were then fixed, permeabilized and processed for confocal microscopy. LeishIF4E-3 was detected using specific antibodies and secondary DyLight antibodies (550nm; red). LeishIF4G-4 was visualized through its fusion with GFP (488 nm; green). Nuclear and kinetoplast DNA was stained using DAPI (blue). Bright field pictures are shown on the right.

S6B Fig

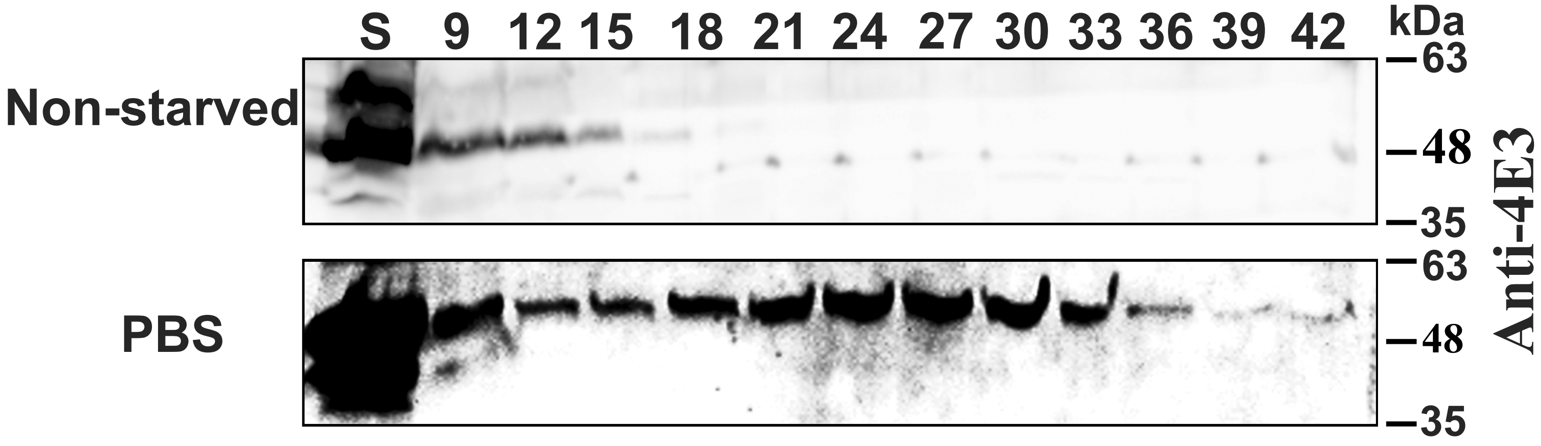

S6B Fig. LeishIF4E-3 migrates in heavy sucrose fractions in the absence of cycloheximide. Transgenic *L. amazonensis* promastigotes expressing SBP-tagged LeishIF4E-3 were incubated in nutrient free buffer (PBS) for 12 h along with non-starved cells as control. Cell extracts were fractionation over 10-40% sucrose gradients in the absence of cycloheximide. Samples from the fractionated proteins were precipitated by TCA, resolved over 12% SDS-PAGE, electroblotted and subjected to western analysis using antibodies against LeishIF4E-3.

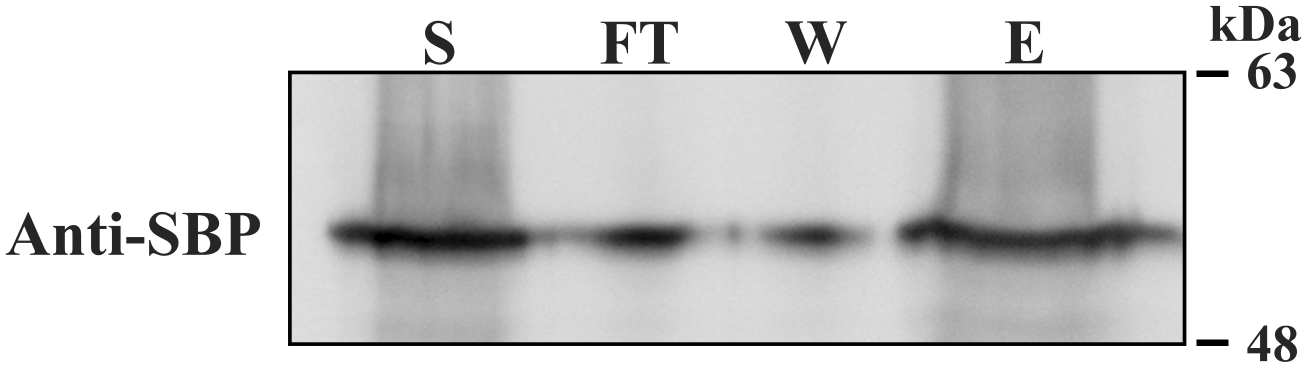

S6C Fig. Pull-down of luciferase-SBP from the heavy fractions of sucrose gradients following PBS starvation. Transgenic *L. amazonensis* promastigotes expressing SBP-tagged luciferase were incubated in nutrient-free buffer (PBS) for 12 h. Cell extracts were fractionated over 10-40% sucrose gradients. Fractions 25–42 were pooled and subjected to pull-down analysis using strepatividin-Sepharose beads. The wash fractions (W, 50%) and eluted (E, 50%) proteins were precipitated by TCA, and resolved by 12% SDS-PAGE, along with samples from the supernatant (S, 10%) and flow through (FT, 10%) fractions. The gel blots were subjected to western analysis using specific antibodies against the SBP tag.

S6D Fig

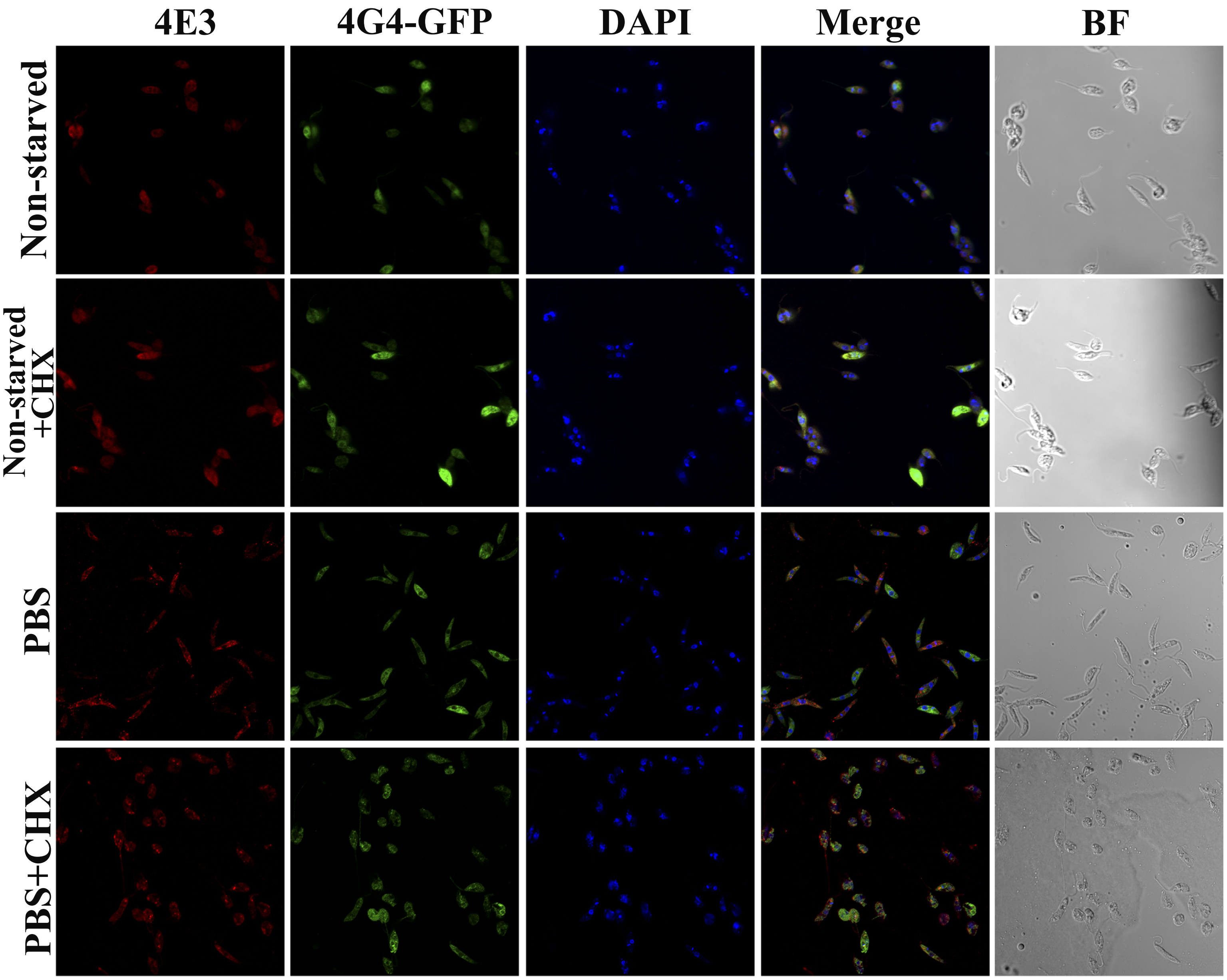

S6D Fig. A field view showing that cycloheximide does not affect LeishIF4E-3 granule assembly following PBS starvation. *L. amazonensis* cells expressing LeishIF4G4-GFP were starved by incubation in PBS for 4 h, either in the presence or absence of cycloheximide (100 μg/ml). The cells were then fixed, permeabilized and processed for confocal microscopy. LeishIF4E-3 was detected using specific antibodies and secondary DyLight antibodies (550 nm; red). LeishIF4G-4 was visualized through its fusion with GFP (488 nm; green). Nuclear and kinetoplast DNA was stained using DAPI (blue). Bright field pictures are shown on the right.

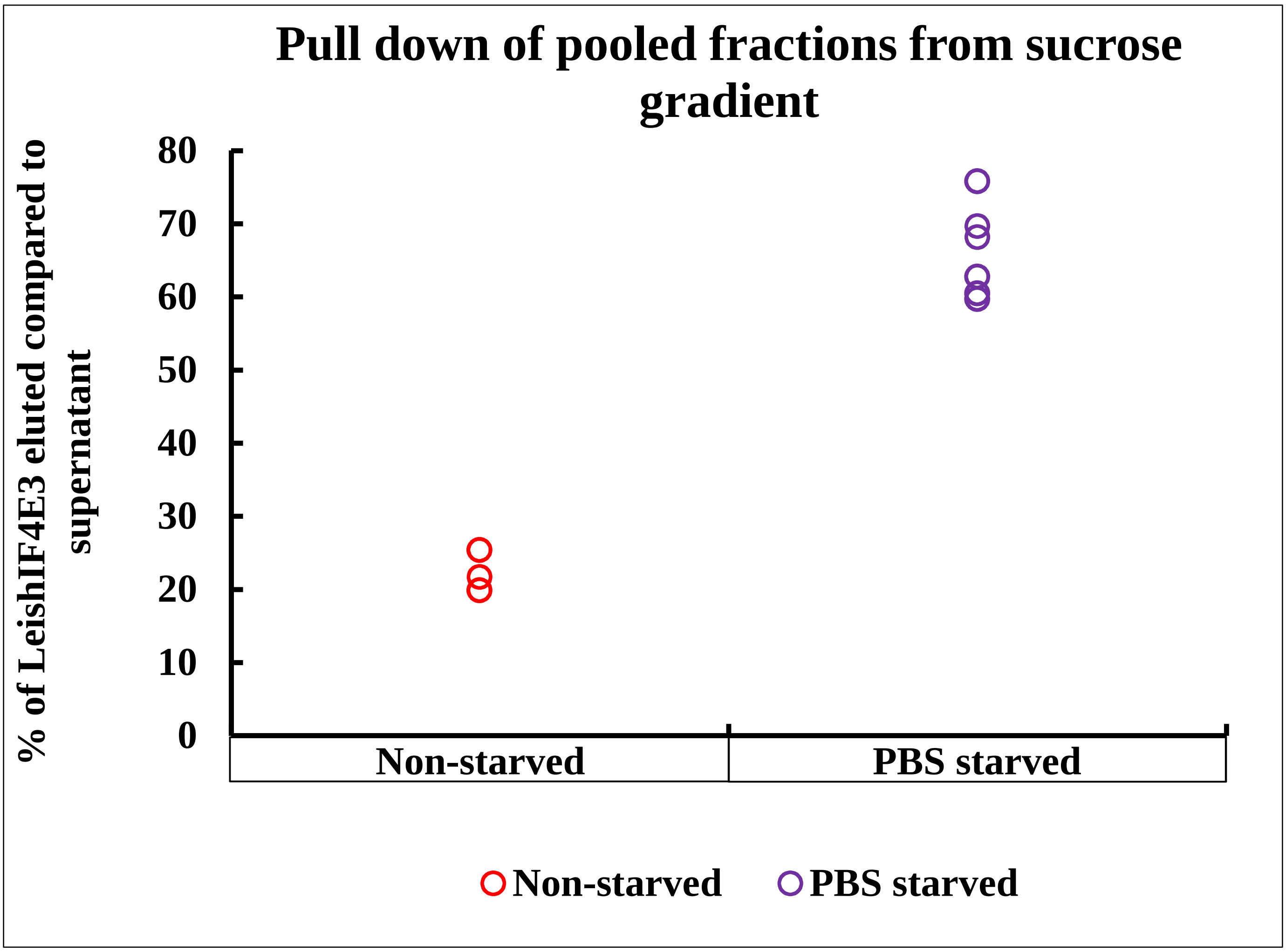

S6E Fig. Densitometric analysis of LeishIF4E-3 pulled-down from heavy sucrose fractions of gradients addressing starved and non-starved parasites. Bands representing the supernatant and eluted gradient fractions separated by SDS-PAGE and blotted (shown in Figure 6B) were quantified using the Multi Gauge, version 2.0 software. Each band from three different experiments of LeishIF4E-3 pulled-down representing non-starved (left bottom panel) or starved cells (right bottom panel) were quantified and values are presented here.
