# Supplementary material for: Nutritional stress targets LeishIF4E-3 to storage granules that contain RNA and ribosome components in Leishmania
Source: PLoS Negl Trop Dis. 2019 Mar 14;13(3):e0007237. doi: 10.1371/journal.pntd.0007237 (PMC6435199; doi:10.1371/journal.pntd.0007237)
Supplement: S7 Fig — Transgenic L. amazonensis promastigotes expressing either SBP-tagged LeishPABP2 (I) or SBP-tagged LeishIF4E-3 (II) were subjected to nutrient starvation (PBS) for 4 h. The cells were then fixed, permeabilized and processed for confocal microscopy. (I) LeishIF4E-3 was immuno-stained with specific rabbit antibodies and secondary DyLight-labeled anti-rabbit antibodies (550 nm; red). LeishPABP2 was detected using mouse monoclonal antibodies against SBP and secondary anti-mouse DyLight antibodies (488 nm; green). (II) RPS6 was detected using specific rabbit antibodies and secondary DyLight anti-rabbit antibodies (550 nm; red). SBP-tagged LeishIF4E-3 was visualized with mouse monoclonal anti-SBP antibodies, detected with DyLight-labeled anti-mouse secondary antibodies (488 nm; green). Nuclear and kinetoplast DNA was stained using DAPI (blue). Bright field pictures are shown on the right. (PDF) [file pntd.0007237.s007.pdf]

Supporting Information S7 Fig

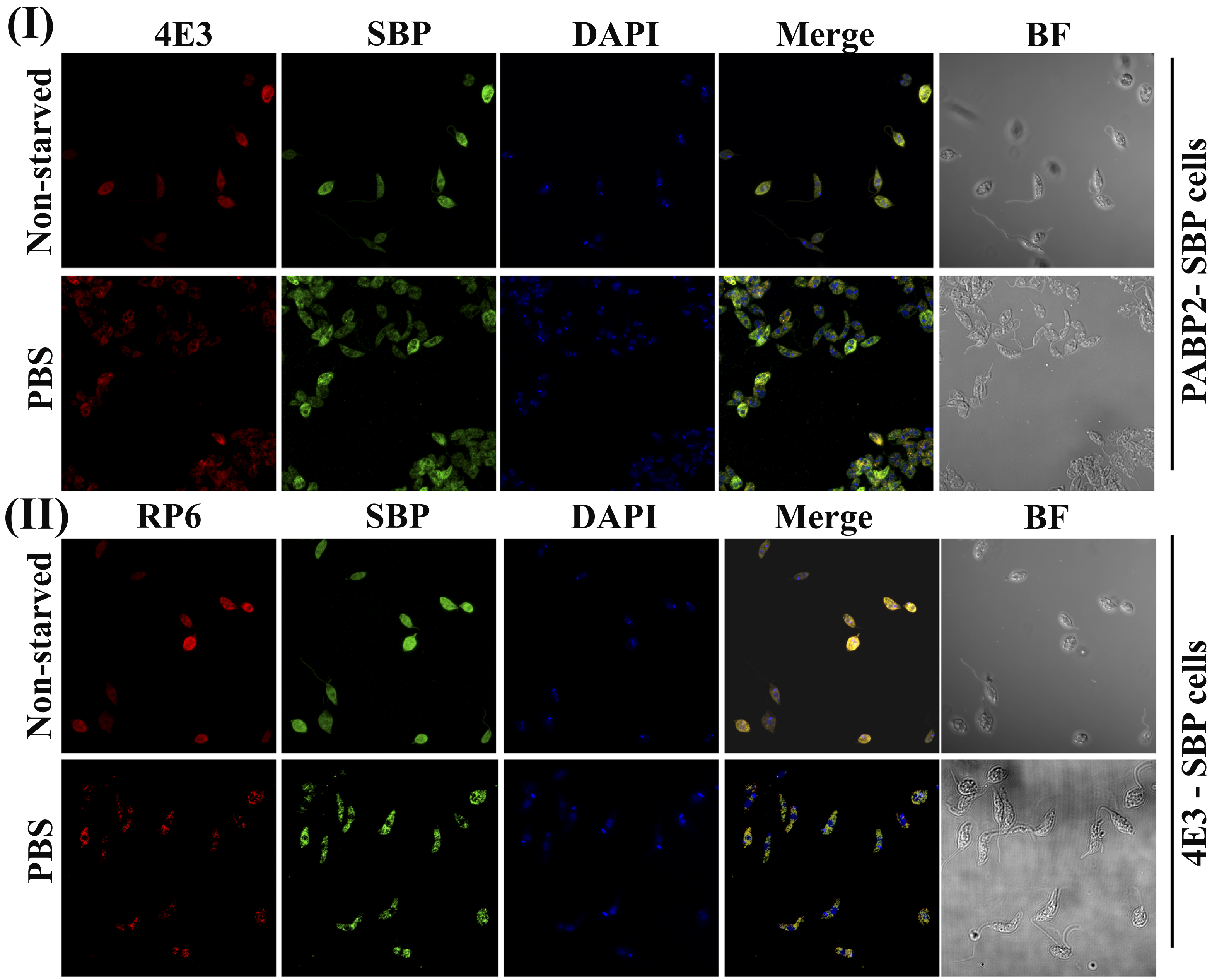

S7 Fig. A field view showing that LeishPABP2-SBP and LeishRPS6 co-localize in starvation-induced LeishIF4E-3 containing granules following nutrient deprivation. Transgenic *L. amazonensis* promastigotes expressing either SBP-tagged LeishPABP2 (I) or SBP-tagged LeishIF4E-3 (II) were subjected to nutrient starvation (PBS) for 4 h. The cells were then fixed, permeabilized and processed for confocal microscopy. (I) LeishIF4E-3 was immuno-stained with specific rabbit antibodies and secondary DyLight-labeled anti-rabbit antibodies (550 nm; red). LeishPABP2 was detected using mouse monoclonal antibodies against SBP and secondary anti-mouse DyLight antibodies (488 nm; green). (II) RPS6 was detected using specific rabbit antibodies and secondary DyLight anti-rabbit antibodies (550 nm; red). SBP-tagged LeishIF4E-3 was visualized with mouse monoclonal anti-SBP antibodies, detected with DyLight-labeled anti-mouse secondary antibodies (488 nm; green). Nuclear and kinetoplast DNA was stained using DAPI (blue). Bright field pictures are shown on the right.
