# Supplementary material for: Nutritional stress targets LeishIF4E-3 to storage granules that contain RNA and ribosome components in Leishmania
Source: PLoS Negl Trop Dis. 2019 Mar 14;13(3):e0007237. doi: 10.1371/journal.pntd.0007237 (PMC6435199; doi:10.1371/journal.pntd.0007237)
Supplement: S8 Fig — Cell growth [top panel: (I)] was monitored by cell count using a Neubauer hemocytometer. Cell viability [bottom panel: (II)] was monitored by cell count using the trypan blue exclusion assay. Non-starved cells are shown in blue, purine-starved cells are shown in red, amino acid-starved cells are shown in purple and glucose-starved cells are shown in black. (PDF) [file pntd.0007237.s008.pdf]

(I)

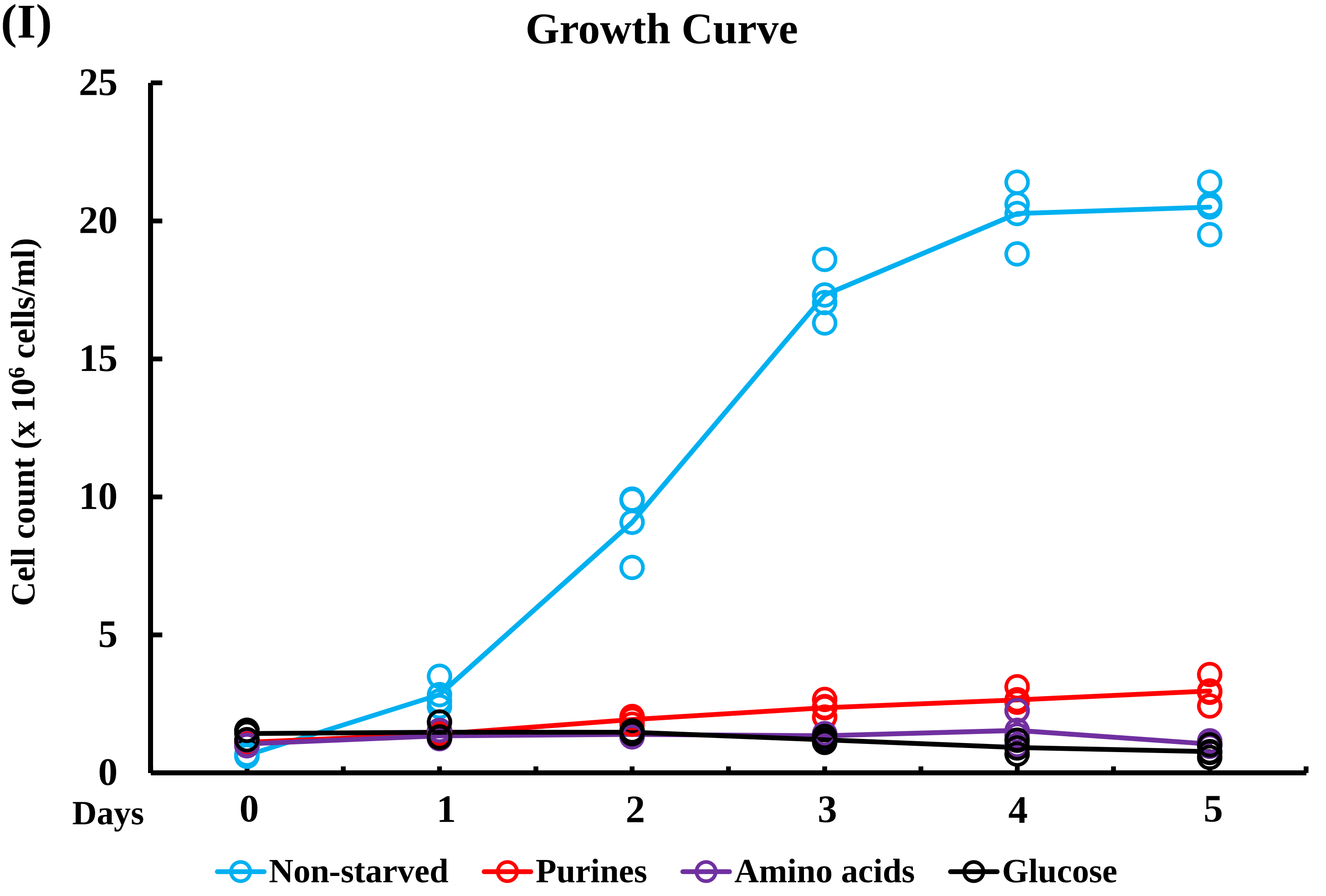

		Cell counts					
**Days**		**0**	**1**	**2**	**3**	**4**	**5**
	**1**	0.68	2.60	7.44	17.04	18.80	21.40
	**2**	0.60	2.40	9.92	18.60	20.60	20.60
	**3**	0.64	3.50	9.88	16.30	21.40	19.50
**Control**	**Mean**	0.64	2.83	9.08	17.31	20.27	20.50
	**1**	1.12	1.30	1.76	2.02	2.26	2.42
	**2**	1.18	1.40	2.00	2.40	2.56	2.92
	**3**	1.02	1.52	2.04	2.66	3.12	3.56
**Purines**	**Mean**	1.11	1.41	1.93	2.36	2.65	2.97
	**1**	1.00	1.54	1.50	1.20	1.00	1.02
	**2**	0.98	1.24	1.38	1.42	1.36	0.96
	**3**	1.18	1.24	1.30	1.42	2.26	1.16
**Amino acids**	**Mean**	1.05	1.34	1.39	1.35	1.54	1.05
	**1**	1.54	1.30	1.42	1.10	0.90	0.74
	**2**	1.54	1.28	1.48	1.32	1.18	1.01
	**3**	1.20	1.84	1.52	1.18	0.68	0.56
**Glucose**	**Mean**	1.43	1.47	1.47	1.20	0.92	0.77

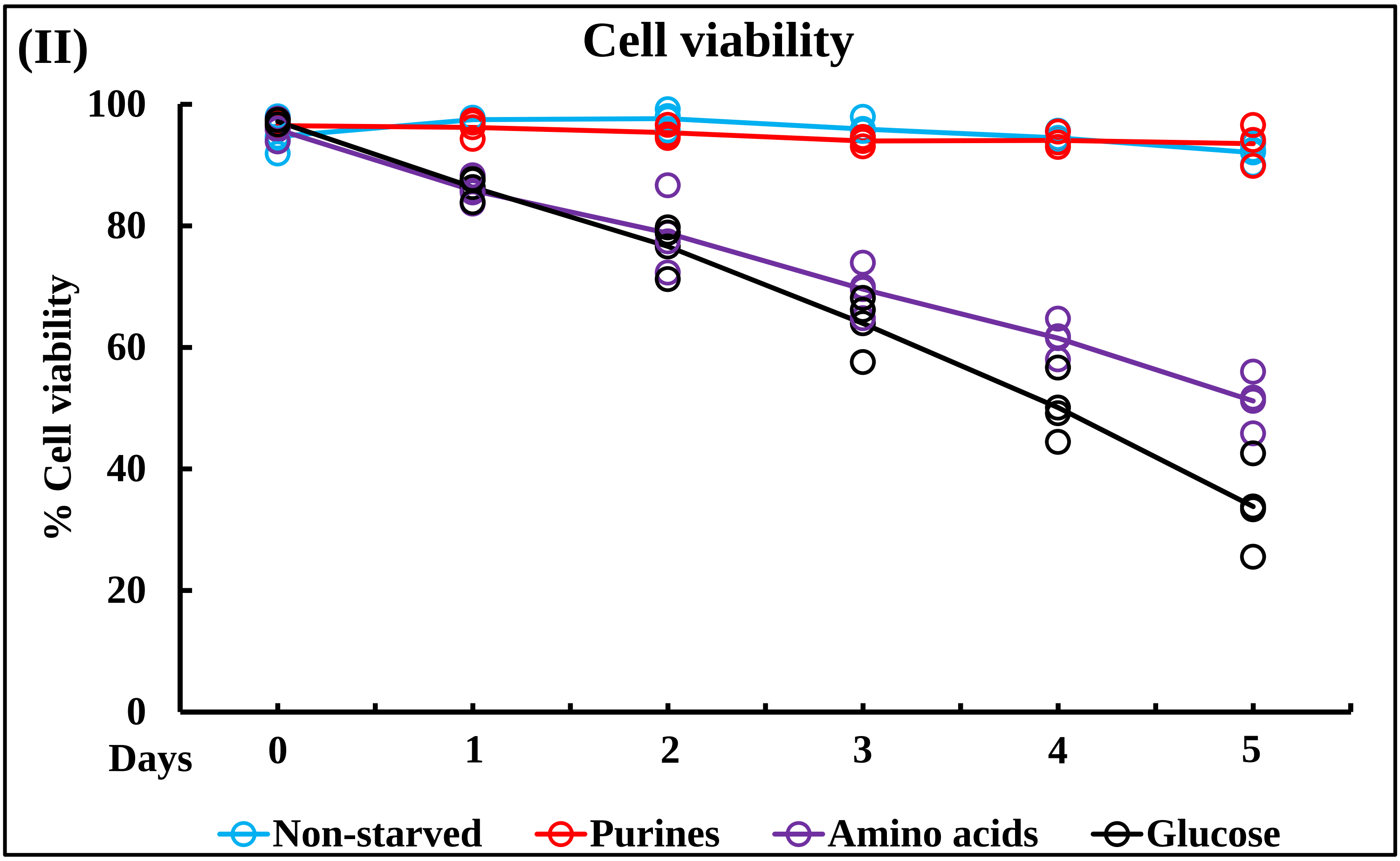

Days		Cell viability counts 0	1	2	3	4	5
Control	1	91.89	97.50	95.70	95.59	95.65	93.59
	2	98.00	97.78	99.19	97.92	94.00	90.07
	3	94.19	97.14	98.04	94.29	93.75	92.50
	Mean	94.69	97.47	97.64	95.93	94.47	92.05
Purines	1	96.97	97.33	96.59	93.07	95.49	94.12
	2	96.43	94.29	95.00	94.17	92.97	96.58
	3	96.08	96.97	94.44	94.62	93.71	89.86
	Mean	96.49	96.20	95.34	93.95	94.06	93.52
Amino acids	1	97.62	88.31	86.67	73.91	61.82	56.00
	2	96.00	85.48	72.31	64.79	58.00	45.83
	3	93.88	83.64	77.42	70.00	64.71	51.72
	Mean	95.83	85.81	78.80	69.57	61.51	51.18
Glucose	1	97.40	87.69	78.87	66.15	56.67	42.55
	2	97.40	87.50	79.73	57.58	49.15	25.53
	3	96.67	83.87	71.21	68.12	44.44	33.33
	Mean	97.16	86.35	76.60	63.95	50.09	33.80

S8 Fig. Growth and viability curves of cultures grown following depletion of specific nutrients. Cell growth [top panel: **(I)**] was monitored by cell count using a Neubauer hemocytometer. Cell viability [bottom panel: **(II)**] was monitored by cell count using the trypan blue exclusion assay. Non-starved cells are shown in blue, purine-starved cells are shown in red, amino acid-starved cells are shown in purple and glucose-starved cells are shown in black.
